# Discovery of natural non-circular permutations in non-coding RNAs

**DOI:** 10.1093/nar/gkad137

**Published:** 2023-03-13

**Authors:** Iris Eckert, Richard Friedrich, Christina E Weinberg, Zasha Weinberg

**Affiliations:** Bioinformatics Group, Department of Computer Science and Interdisciplinary Centre for Bioinformatics, Leipzig University, Härtelstraße 16–18, 04107 Leipzig, Germany; Institute of Biochemistry, Leipzig University, Brüderstraße 34, 04103 Leipzig, Germany; Institute of Biochemistry, Leipzig University, Brüderstraße 34, 04103 Leipzig, Germany; Bioinformatics Group, Department of Computer Science and Interdisciplinary Centre for Bioinformatics, Leipzig University, Härtelstraße 16–18, 04107 Leipzig, Germany

## Abstract

Research in the last two decades has increasingly demonstrated that RNA has capabilities comparable to those of proteins, for example the ability to form intricate 3D structures necessary for catalysis. Numerous protein domains are known in varied within-domain rearrangements, called permutations, that change the N- to C-terminal order of important amino acids inside the domain, but maintain their 3D locations. In RNAs, by contrast, only simple circular permutations are known, in which 5′ and 3′ portions of the molecule are merely swapped. Here, we computationally find and experimentally validate naturally occurring RNAs exhibiting non-circular permutations of previously established hammerhead ribozyme RNAs. In addition to the rearranged RNAs, a bioinformatics-based search uncovered many other new conserved RNA structures that likely play different biological roles. Our results further demonstrate the structural sophistication of RNA, indicate a need for more nuance in the analysis of pseudoknots, and could be exploited in RNA-based biotechnology applications.

## INTRODUCTION

The biochemical function of proteins and RNAs often depends on the precise placement of specific amino acids or nucleotides in 3D space. However, large parts of the molecules have a considerable amount of flexibility in their precise sequence and structure. One manifestation of this flexibility is permutations (1–3), where the order of amino acids or nucleotides is altered. As long as the essential structural elements are retained, the permuted sequence is also functional.

Proteins are often divided into domains, which are amino acid sequences whose folding does not greatly depend on other parts of the protein (4). Domains can thus easily be shuffled within proteins. Permutations can also occur within protein domains (3), although such permutations are much less common. The most common type of rearrangement is a circular permutation, which is a simple reordering in which the N- and C-terminal parts of a domain are effectively swapped (1,3,5). For example, the possible circular permutations of the sequence CYCLE are YCLEC (swapping C and YCLE), CLECY, LECYC and ECYCL. Non-circular permutations are also known in proteins, although these are even rarer. Non-circular swaps of stretches of amino acids in a protein have been documented that preserve the 3D positions of critical groups (3,5,6), and reversals of the N- to C-terminal order of amino acids might also preserve function (6). Multiple swaps and reversals could even be combined to form yet more complex within-domain rearrangements (6). In addition to the insights they enable into protein structure, protein rearrangements have been exploited in engineering applications (1).

Similarly to proteins, RNA can perform such structurally demanding tasks as catalysis (7,8) and highly specific recognition of small molecules and ions (9). Although proteins are used more often than RNA in such roles in modern organisms, the last two decades have shown an increased recognition of natural RNAs that go well beyond a simple coding function. Thus, one might suspect that RNAs could also be found in multiple permuted forms.

However, to date, only circular permutations have been identified in natural RNAs, specifically in the hammerhead and twister ribozymes (2,10,11), 5S rRNAs (12) and group-II introns (13). tmRNA genes in certain bacteria are present in a permuted form, although their transcripts are processed into two separate RNAs that together function as a tmRNA (14). A similar phenomenon occurs in rRNA genes in *Tetrahymena pyriformis* (15). rRNAs in some archaea are circularly permuted post-transcriptionally by a circularization reaction followed by cleavage at a new site, and these actively permuted rRNAs are functional (16). Some tRNA genes encode fragments of the tRNA in the reversed order, however their transcripts are circularized and cleaved so as to ultimately recover the standard tRNA form (17), and a broadly similar phenomenon is observed in a protozoan mitochondrial tmRNA (18). Circularly permuted SRP RNA genes give rise to a transcript that is then circularized and functions in its circular form (19). Importantly, no non-circular permutations are known in natural RNAs.

We set out to discover new forms of self-cleaving ribozymes, which are RNAs that catalyze the separation of a phosphodiester bond at a specific position in themselves (10,11,20). Such ribozymes show RNAs in one of the most structurally challenging tasks it is known to perform, and are also useful in various biotechnology applications (21).

The search strategy we adopted was based on our recent work on hairpin ribozymes. Although discovered in 1986 (22), only four unique hairpin ribozyme sequences were known for decades (23). However, using similarity searches, we found over 900 unique new hairpin ribozyme sequences in metatranscriptome data (23). Nearby to these hairpin ribozymes, but on the opposite strand, we often found examples of hammerhead and twister ribozymes, as well as other hairpin ribozymes (23). The fact that three unrelated structural classes of self-cleaving ribozymes occur on the opposite strand to hairpin ribozymes suggests that a self-cleaving ribozyme is required, but its structural class is unimportant. However, the sequences on the opposite strand of many hairpin ribozymes lacked a known self-cleaving ribozyme. These sequences, in which a ribozyme is expected, but absent, might contain unknown kinds of ribozymes awaiting discovery. In the current work, we therefore decided to collect non-coding sequences nearby to the metatranscriptomic hairpin ribozymes and to use a comparative method (24) to discover RNA structures conserved within these sequences. Such RNA structures could be new kinds of self-cleaving ribozymes. We call this a hairpin-ribozyme-based ribozyme discovery strategy.

The logic of this hairpin-ribozyme-based strategy is also mirrored by the role that self-cleaving ribozymes play in the replication of virus-like organisms whose genomes are single-stranded circular RNAs (10,11). Some such organisms use a self-cleaving ribozyme to facilitate the process of creating reverse-complement forms of the genome. A self-cleaving ribozyme encoded in the antisense direction then assists the creation of a sense genome from the antisense form. This process is known as symmetric rolling-circle replication, and requires ribozymes in both strands. Some organisms use only one self-cleaving ribozyme in total, and implement an asymmetric form of rolling-circle replication. We hypothesized that the recently discovered hairpin ribozymes, together with their opposite-strand ribozymes, use symmetric rolling-circle replication. If this hypothesis is true, then other kinds of self-cleaving ribozymes might occur in the antisense orientation to the hairpin ribozymes to enable symmetric rolling-circle replication.

The hairpin-ribozyme-based strategy is analogous to previous work in which we found that bacterial hammerhead and twister self-cleaving ribozyme classes are both enriched in the vicinity of specific genes (25,26). These ribozyme-enriching genes varied in function, but the strongest ribozyme associations were for genes related to Mu-like phages (25,26). Additionally, self-cleaving ribozymes were enriched in bacteria nearby to other self-cleaving ribozymes (25,26). Although the biological reason for these associations is still unknown, the presence of two ribozyme structural classes in similar locations suggested that any type of self-cleaving ribozyme could be found in such locations. Therefore, in this previous work, we collected non-coding regions nearby to the ribozyme-associated genes and investigated them for new RNA structures. Using this gene-based ribozyme discovery strategy, we previously found three RNAs that were new classes of self-cleaving ribozymes, namely pistol, hatchet and twister-sister ribozymes (26). The previously published gene-based strategy and the new hairpin-ribozyme-based strategy both exploit locations in which multiple classes of self-cleaving ribozymes are commonly present.

Nine well-studied structural classes of self-cleaving ribozymes are currently known (10,20), with an additional class recently proposed (27). Two, hammerhead and twister ribozymes, are naturally found in circular permutations. The three previously known circular permutations of the hammerhead ribozyme are called type I, type II and type III. Type-III (28) and type-I (29) permutations were discovered early. Experiments suggested the possibility of type-II hammerhead ribozymes (2,30). However, natural examples of this permutation were only identified decades after types I and III (2). The twister ribozyme was first found in a type-P1 permutation (25). Based on the precedent of hammerhead permutations, searches were previously conducted for expected circular permutations, leading to the identification of type-P3 and type-P5 twister forms (25). Given that examples of all possible circular permutations have been found and that non-circular permutations are unknown in natural RNAs, scientists (including us) have generally believed that all permuted forms of hammerhead and twister ribozymes have been discovered.

## MATERIALS AND METHODS

### Databases used

Metatranscriptomic and metagenomic sequences were downloaded predominantly from IMG/M (31) and GenBank (32). Additional searches were conducted on RefSeq (33) version 85. Environmental metadata were isolated from the relevant metagenomic or metatranscriptomic source. Genes were annotated as previously described (24). Known RNAs were annotated using Rfam (34) version 14.0.

### Bioinformatics analysis of novel conserved RNAs

Non-coding regions in the same sequence contigs as previously predicted hairpin ribozymes (23) were extracted, and subjected to a system to detect novel structured RNAs, essentially as described (24). Briefly, sequences were clustered using BLAST (35) version 2.2.26 and overcluster2 (24) version 1.0.1. Conserved sequences in clusters were predicted using CMfinder (36) and scored as described (24) with version 0.4.1.18 of the CMfinder package. Promising predictions were further evaluated with repeated rounds of homology searches using Infernal (37) version 1.1, and structural analysis with CMfinder, R-scape (38) and manual analysis. Such analysis can improve predictions beyond the initial CMfinder prediction, e.g. by finding additional sequences that provide covariation to support additional stems, including pseudoknots. Consensus features were determined and drawn using R2R (39), but as before (40), we used both R-scape and R2R to depict covariation.

### Searches for additional permutations

We searched for previously unknown permuted forms of hammerhead and twister ribozymes as well as for new examples of the type-SGC permutation using search patterns (Supplementary File S1) we defined with the DARN! (41) or RNAMotif (42) software. Due to the large number of sequences searched, we expected false positive matches. As before (23), we conducted additional homology searches using the Infernal software (37) on each candidate sequence discovered with DARN! or RNAMotif, and manually evaluated Infernal's predicted alignments for covariation. Only alignments that exhibited compelling covariation (40) were considered to be promising. We further improved these alignments with iterative use of Infernal searches, up to an *E*-value of 10, and manual adjustment of the resulting alignments, including rejection of sequences we manually judged as unlikely matches. The most common reasons for removal of a sequence arose when extremely few predicted base pairs were C–G, or when the predicted structure involved a very long insertion whose nucleotides did not fold into a hairpin that would plausibly allow the overall hammerhead or twister ribozyme structure to form (23). When we previously applied this protocol to the search of hairpin ribozymes, we found that five predicted sequences with the poorest *E*-values (ranging from 4.8 to 7) that passed our manual analysis functioned *in vitro* as self-cleaving ribozymes (23). We searched all metagenomic and metatranscriptomic sequences we collected as well as all RefSeq sequences.

### Ribozyme self-cleavage during *in vitro* transcription

Double-stranded DNA templates containing a T7 promoter were generated by elongating partly complementary oligonucleotides (Biomers, Supplementary Table S1) with Phusion DNA polymerase (ThermoScientific). *In vitro* transcriptions of the wildtype and mutated ribozymes were performed at 37°C using T7 RNA polymerase (50 U), TIPP (2 U/μl), 100 ng of DNA template in 80 mM HEPES (pH 7.5), 25 mM MgCl_2_, 10% DMSO and 5 mM DTT. The DNA template used for transcription was a phenol/chloroform extracted and ethanol precipitated PCR product. NTPs were present in the reaction mixture at a final concentration of 2.5 mM, supplemented with traces of [α-^32^P] ATP (Hartmann Analytik). Internally-^32^P-labeled products and 5′-labeled RNA size standards (see below) were separated using denaturing (urea) 20% polyacrylamide gel electrophoresis (PAGE) and respective bands detected using the Amersham Typhoon imaging system (GE Healthcare).

### Generation of RNA size standards

For production of size standards, cleavage-deficient ribozyme mutants were transcribed without the addition of [α-^32^P] ATP, then separated via 10% denaturing PAGE. Relevant RNA bands were isolated from the gel by soaking in a solution containing 10 mM Tris–HCl (pH 7.5), 200 mM NaCl and 1 mM EDTA (pH 8.0). After ethanol precipitation, cleavage-deficient ribozyme RNAs were dephosphorylated at their 5′ end using Antarctic phosphatase (NEB) according to manufacturer's instructions, followed by phenol/chloroform extraction and ethanol precipitation. The dephosphorylated ribozyme mutant was 5′-labeled with [γ-^32^P] ATP (Hartmann Analytik) and T4 polynucleotide kinase (NEB) according to the manufacturer's instructions. The isolated 5′ ^32^P-labeled cleavage deficient mutant was partially digested with RNase T1 (T1-ladder) or with alkali (^−^OH-ladder).

### Determining ribozyme cleavage kinetics *in cis*

We investigated ribozyme cleavage speeds during *in vitro* transcription (cleavage *in cis*). *In vitro* transcription reactions were set up including [α-^32^P] ATP as described above, except for using an altered rNTP mix (final concentration: 2.5 mM of each GTP, CTP, UTP, 0.25 mM ATP). Magnesium chloride was added last to initiate the reaction. Aliquots were withdrawn at designated time intervals and mixed with stop solution (90% formamide, 50 mM EDTA, 0.05% xylene cyanol and 0.05% bromophenol blue). Internally-^32^P-labeled products were separated using denaturing (urea) 10% polyacrylamide gel electrophoresis (PAGE) and respective bands detected using the Amersham Typhoon imaging system (GE Healthcare).

Observed cleavage rates were calculated as described previously (43) by plotting the fraction of L/(L + S) versus time (*t*), where L is the full-length transcript and S is the cleaved transcript. The data were fitted to a one-phase exponential decay using GraphPad Prism. The values for all mean rate constants reported are the averages of a minimum of two replicate assays.

## RESULTS

### Discovery of novel RNAs

To find novel self-cleaving ribozymes we applied a hairpin-ribozyme-based strategy using 941 previously identified hairpin ribozymes (23). These hairpin ribozymes occur in metatranscriptomes, which consist of fully or partially determined transcriptomic sequences called contigs. We collected all predicted non-coding regions present in the same contig as a hairpin ribozyme. These non-coding regions were subjected to a comparative analysis (24) that attempts to find conserved RNA secondary structures, and outputs structural alignments. We manually analyzed the predictions to find alignments that were likely to correspond to biological RNAs. This analysis focused on the concept of covariation, which describes compensatory mutations that conserve a proposed RNA secondary structure despite distant changes to the primary sequence. Such mutations are inevitable over sufficient evolutionary time with structured RNAs, but are not enriched in other types of genomic elements. Thus, covariation is a specific signal of an RNA that conserves a functionally important structure. We ultimately conducted these covariation analyses manually because of issues like the importance of ensuring that the sequences are correctly aligned in order to evaluate covariation meaningfully. Promising alignments were further analyzed by repeated homology searches and refinement of the proposed structure, as previously (24), and many were rejected based on this additional analysis. We searched for motif homologs within a broad set of metatranscriptomes, metagenomes and genomes of known organisms (data sources are listed in Materials and Methods). Alignments that we judged to be promising after this process were called ‘motifs’, and these are also candidate self-cleaving ribozymes due to their association with hairpin ribozymes.

### An unexpected hammerhead ribozyme permutation

**Figure 1.**
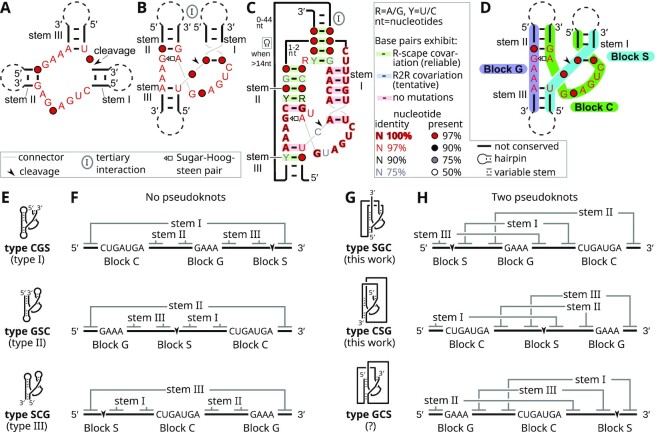
Classic and novel permuted forms of hammerhead ribozymes. (**A**) Schematic of the hammerhead ribozyme core in the traditional layout, with stems I, II and III and highly conserved nucleotides (red). Circles indicate nucleotide positions with low conservation. The arrowhead marks the cleavage site. Dashed lines represent loops that can be absent, as in previously published permutations: type I (5′ and 3′ ends occur instead of the stem I loop), type II (instead of stem II loop) and type III (instead of stem III loop). The inset describes annotations also used in subsequent RNA drawings. Note: the sub-figure's layout resembles a previous depiction (47). (**B**) The structure in part A drawn in the modern layout, which reflects an important interaction between stems I and II (circled ‘I’). (**C**) Conserved features of newly discovered hammerhead-ribozyme-like RNAs, also called the HRIMA-1 motif, in the layout of part B. All red nucleotides and stems from part B are also conserved in the newly discovered RNAs. The connectivity differs profoundly from previously known hammerhead ribozymes. The inset describes annotations also used in the remainder of this paper. (Not all annotations are used in this figure.) (**D**) The hammerhead ribozyme in the modern layout divided into three blocks (see text) that we believe can occur in any order. (**E**) Schematic diagrams of the three previously known hammerhead ribozyme permutations. Our proposed permutation names (bold font) indicate the 5′ to 3′ ordering of the blocks in part D. Traditional names (e.g. ‘type I’) are also given. The connectivity of stems I, II and III is shown for each permutation. (**F**) The core region of the classic permutations in part E are drawn from 5′ to 3′ on lines. Conserved features (from part A) and the three blocks (from part D) are marked. Stems I, II and III are shown as lines connecting base-pairing regions, and form a three-stem junction with no pseudoknots, as in part A. (**G**) Schematic depictions of the novel permutations, for comparison with part E. The molecule in part C corresponds to type SGC. Data support the existence of type-SGC and, to a lesser extent, type-CSG ribozymes (marked ‘this work’), but evidence for the biological relevance of type-GCS ribozymes is unclear (marked ‘?’). (**H**) Linear depictions of the permutations from part G. It is impossible to depict the stems without crossing lines, as they each contain at least two pseudoknots.

We found 26 candidate structured RNAs, which we termed Hairpin-Ribozyme In Metatranscriptome Associated (HRIMA) motifs (Supplementary Table S2, Supplementary Files S2 and S3, Supplementary Figures S1–S5). Our top candidate, initially named HRIMA-1, exhibited significant covariation and an intricate structure with multiple pseudoknots. We noticed a conserved CUGAnGA sequence (where ‘n’ is any nucleotide), which is typical of hammerhead ribozymes (2). However, it was not possible to fit the motif into any previously published forms of hammerhead ribozymes (Figure 1A, B). Rather, we ultimately hypothesized that the new HRIMA-1 motif presented all conserved elements of hammerhead ribozymes in an unexpected 5′-to-3′ order (Figure 1C, Supplementary Figure S6). In particular, all highly conserved (red) nucleotides and stems in the hammerhead ribozyme core (Figure 1A, B) are also conserved in the new motif (Figure 1C). Moreover, statistically significant covariation according to the R-scape software (38) was observed (Supplementary Table S2), which supports the biological relevance of stems II and III that we propose correspond to hammerhead ribozymes. Our subsequent analysis, described below, also revealed covariation in stem I. The apparently rearranged ribozyme is not an artefact caused by misassembly of metatranscriptome reads, as many ribozymes are entirely contained within a single raw read, and we have confirmed an example using reverse transcription and sequencing (Supplementary Note S1).

Given the hypothesis of an unusual ribozyme permutation, we analyzed the HRIMA-1 motif's relationship to hammerhead ribozymes and speculated on what other permuted forms would be possible. The conserved CUGAnGA sequence in hammerhead ribozymes is located adjacent to stems I and II (Figure 1A, B). In all currently known hammerhead ribozyme alignments, there are no insertions of more than one or two nucleotides that occur between the CUGAnGA sequence and the adjacent stems. Therefore, we assume that the CUGAnGA sequence and its adjacent paired nucleotides must occur directly after one another in the same 5′-to-3′ order. We call this ‘Block C’ (for CUGAnGA) (Figure 1D).

Similarly, ‘Block G’ consists of the conserved GAAA sequence and the adjacent parts of stems II and III, and ‘Block S’ consists of the site of cleavage and proximal sides of stems I and III (Figure 1D). Assuming that the components of these blocks cannot be interrupted, there are six orders in which the blocks can occur, thus six possible permutations of the ribozymes. In this paper, we call these permutations types SCG, SGC, CGS, CSG, GSC and GCS (Figure 1E–H). The previously discovered permutations, types I, II and III, correspond to types CGS, GSC and SCG, respectively, while the newly found non-circular permutation of hammerhead ribozymes (Figure 1C) is type SGC.

Hammerhead ribozymes generally have a tertiary interaction between the loops of stems I and II that accelerates cleavage by bringing these stems close to each other (2,44–46). This interaction can take the form of non-Watson-Crick interactions (46) or a helix (47). In the newly found type-SGC hammerhead ribozymes (corresponding to the HRIMA-1 motif), one side of stem I is immediately connected to one side of stem II via a linker region of typically three nucleotides (Figure 1C). Thus, the connectivity of type-SGC hammerhead ribozymes might help to bring stems I and II together. We also observe a stem that is expected to bind the other sides of stem I and II to one another (Figure 1C, circled ‘I’).

We noticed that stem I in the newly found type-SGC hammerhead ribozymes consists of five base pairs with perfectly conserved nucleotides. Such conservation is not observed in previously identified hammerhead ribozymes, and there is no established biochemical or structural need for fixed nucleotides in stem I. To determine if other nucleotides are permitted in type-SGC ribozymes, we performed a *de novo* search for sequences that exhibit the expected sequence and secondary-structure features theoretically expected of type-SGC ribozymes (see Methods). We removed likely false positive matches based on covariation, as in previous analogous searches for hairpin ribozymes (23). This search of RefSeq, metagenome and metatranscriptome sequences (see Methods) revealed additional type-SGC hammerhead ribozymes. We call these sequences type-SGC hammerhead ribozyme variants to distinguish them from the HRIMA-1 motif. The type-SGC variants have different sequences in stem I (Supplementary Figures S7 and S8), and their stem I sequences exhibit covariation. Thus, in keeping with previous work on hammerhead ribozymes, there is no requirement for specific nucleotides in stem I in type-SGC hammerhead ribozymes, and we observe covariation in stems I, II and III. We do notice that some nucleotides within stem I and outside of the ribozyme core (Figure 1A, B) are relatively conserved within the HRIMA-1 motif and, with distinct sequences, among the type-SGC variants (Figure 1C, Supplementary Figure S7). This conservation could reflect a short evolutionary distance between the ribozyme sequences, or it might correspond to tertiary structures that are structurally important for enabling these unusual permuted forms of hammerhead ribozymes.

### Experimental validation of type-SGC hammerhead ribozymes

To investigate our hypothesis that the proposed type-SGC hammerhead ribozymes are indeed a form of hammerhead ribozymes, we sought to experimentally validate the expected cleavage properties. Assuming that the sequences do indeed cleave themselves, the cleavage site could in principle be located anywhere within each sequence. However, based on the hypothesis that the new sequences correspond to hammerhead ribozymes, we can predict the precise cleavage position by structural analogy (Figure 1C). Only cleavage at this exact location is consistent with our hypothesis.

**Figure 2.**
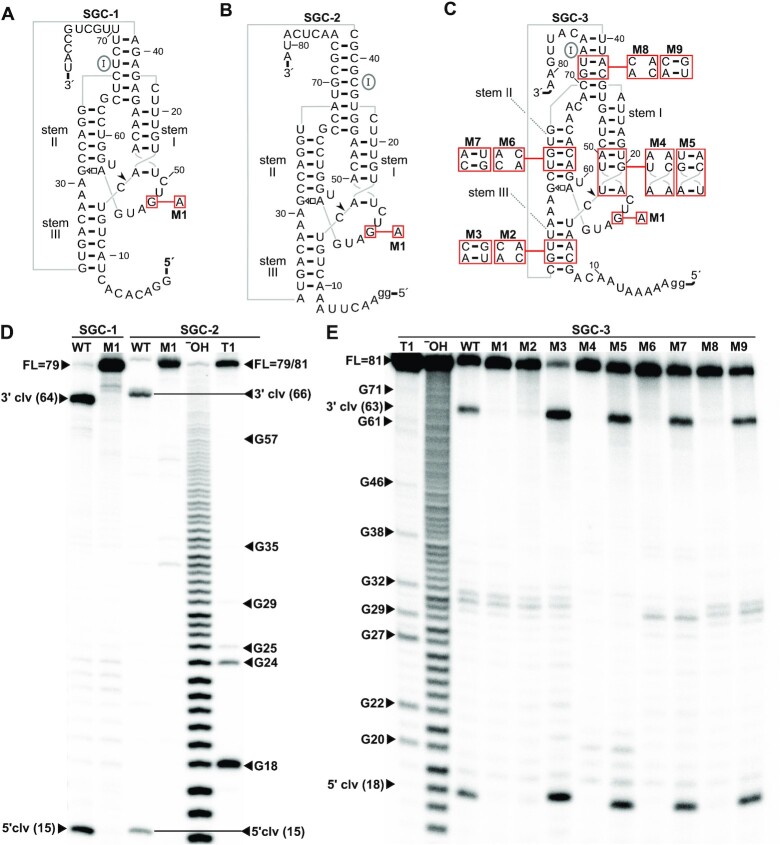
Co-transcriptional cleavage of type-SGC hammerhead ribozymes. (**A**) Secondary structure diagram of SGC-1, an example of the HRIMA-1 motif (Figure 1C). Annotations in parts A-C are as in Figure 1C. M1 indicates a ribozyme mutant expected to lead to a cleavage-deficient ribozyme (48). (**B**) Secondary structure diagram of SGC-2, an example of the HRIMA-1 motif. Lower-case g nucleotides were added to improve *in vitro* transcription. (**C**) Secondary structure diagram of SGC-3, a type-SGC ribozyme variant (Supplementary Figure S7). M2, M4, M6 and M8 denote mutations that disrupt stems I, II, III or the interaction between stems I and II. M3, M5, M7 and M9 designate compensatory mutations that restore the integrity of each stem. (**D**) PAGE image showing SGC-1 and SGC-2 candidate ribozymes after *in vitro* transcription in the presence of [α-^32^P] ATP for 2 h. 5′-radioactively labeled M1 RNA was used to create size standards by partial digestion with RNase T1 (‘T1’) or by alkaline hydrolysis (‘^−^OH’). Transcript bands for WT (wildtype) ribozyme RNA correspond to the 3′ cleavage products (64 and 66 nucleotides, respectively) and a 5′ cleavage product of 15 nucleotides for both candidates. The full-length (FL) transcripts of M1 ribozyme mutants are visible at 79 and 81 nucleotides, respectively. (**E**) PAGE analysis of SGC-3 after *in vitro* transcription with [α-^32^P] ATP for 2 h. M1 RNA was used to create ladders using partial digestion with (‘T1’) or alkaline hydrolysis (‘^−^OH’). The full-length molecule (‘FL’) is marked, as are selected G nucleotides and the expected location of the 5′ and 3′ cleavage products (18 and 63 nucleotides, respectively). Cleavage occurs robustly in the wildtype (‘WT’) SGC-3 molecule as well as M3, M5, M7 and M9 and is barely visible, if at all, for M1, M2, M4, M6 and M8.

To investigate the newly identified type-SGC hammerhead ribozymes’ cleavage properties, we monitored their co-transcriptional cleavage *in vitro*. For this, we chose three representative sequences. Two sequences, termed SGC-1 and SGC-2 (Figure 2A, B), conform well to the secondary structure consensus and contain all highly conserved nucleotides of the HRIMA-1 motif (Figure 1C, Supplementary Figure S6). The third sequence, designated SGC-3 (Figure 2C), fits the type-SGC hammerhead ribozyme variant alignment well (Supplementary Figures S7 and S8). After *in vitro* transcription in the presence of [α-^32^P] ATP, we separated the transcription products by PAGE and observed distinct bands corresponding to the expected cleavage products (Figure 2D, E, Materials and Methods). For all representatives, we also generated a cleavage-deficient mutant (M1) by changing the highly conserved G within the first part of the CUGAnGA sequence. This mutation was previously established to abolish cleavage in hammerhead ribozymes (46,48–51). The mutant ribozymes were used as size standard by partial alkaline digest or RNase T1 mediated cleavage and served as full-length ribozyme controls (Supplementary Table S1, Materials and Methods). Co-transcriptional cleavage of SGC-1 and SGC-2 wildtype sequences is nearly complete, as almost no full-length product can be observed (Figure 2D). Importantly, cleavage product lengths show that cleavage occurs precisely at the position predicted based on structural analogy with hammerhead ribozymes (Figure 1C). By contrast, the M1 mutant molecules do not exhibit visible cleavage, which is consistent with the effect of this mutation on previously studied hammerhead ribozymes. In analogous experiments, we also tested SGC-3 (Figure 2C), the type-SGC ribozyme variant sequence (Figure 2E). SGC-3 also cleaved site-specifically into the expected 5′ and 3′ products, while the M1 mutant version remained at full-length.

To confirm the computationally predicted secondary structure of type-SGC ribozymes, we mutated the SGC-3 sequence to individually disrupt each of its four conserved stems: stems I-III and the interaction between stems I and II (mutant sequences M2, M4, M6 and M8, Figure 2C, E). Additionally, we generated SGC-3 sequences where each disruptive mutation was compensated by a mutation at another location, thus restoring base-pairing in the affected stem (mutant sequences M3, M5, M7, M9, Figure 2C, E). As expected, when any of the stems is disturbed, only a full-length ribozyme band can be observed, showing that the ribozyme no longer functions (Figure 2E). In each stem, the function is restored by compensatory mutations (Figure 2E). These experiments further support the importance of all four proposed stems for catalytic activity.

Among the newly predicted type-SGC ribozymes (both from the HRIMA-1 motif and the type-SGC variant), we noticed three sequences that contain deviations in nucleotide positions that are generally conserved in hammerhead ribozymes. These are the only atypical sequences out of 583 predicted type-SGC ribozymes (comprised of 407 sequences in the HRIMA-1 motif and 176 type-SGC variant sequences). The first deviation we observed is an A to U mutation in the last nucleotide of the conserved CUGAnGA sequence (Supplementary Figure S9A), which previously reduced cleavage by at least 20-fold in an artificial sequence (48), but was subsequently found in a functional, natural ribozyme (52). The second is a mutation of the normally conserved A-U pair in stem III to U-A (Supplementary Figure S9B). This mutation reduced cleavage by at least 500-fold in an engineered sequence (48). However, this mutation was also found naturally present in a functional hammerhead ribozyme (47). These two RNA sequences among our predictions similarly cleave *in vitro* (Supplementary Figure S9D). The third predicted ribozyme appears to be missing the two nucleotides immediately 3′ to the cleavage site (Supplementary Figure S9C). This mutation is not expected to be functional, due to the disrupted cleavage site, and we did not observe *in vitro* cleavage (Supplementary Figure S9D). However, this deviant sequence is very rare in the metatranscriptomic RNA-seq sample, and might correspond to a deleterious mutation that has persisted for a short time. All other positions believed to be critical for hammerhead cleavage (2,48) are perfectly conserved in all predicted type-SGC hammerhead ribozymes.

In summary, five candidate type-SGC ribozymes (Figure 2, Supplementary Figure S9A, B) that were expected to be functional cleaved effectively during *in vitro* transcription, producing cleavage fragments of the predicted sizes. Thus, cleavage occurs at sites consistent with previous studies of hammerhead ribozymes. For another type-SGC-variant with a mutation that removes the cleavage site (Supplementary Figure S9C), no activity was expected or observed. Moreover, the M1 mutation of all five ribozymes reduces cleavage significantly, as previously observed for other types of hammerhead ribozymes. Furthermore, the four stems forming the proposed hammerhead-ribozyme-like structure were individually validated by experiments with disruptive mutations, which impede cleavage, and compensatory mutations, which restore ribozyme activity. These facts, and the similar patterns of sequence and structural conservation, are consistent with our hypothesis that the proposed hammerhead-like RNAs are indeed structurally related to hammerhead ribozymes, and that they are non-circular permutations of them.

### Do other permutations occur in nature?

At this point, four of the theoretical six permutations (Figure 1E–H) have been demonstrated to occur in nature: the previously established type CGS (type I), type GSC (type II) and type SCG (type III), and the new permutation, type SGC. We searched genome, metagenome and metatranscriptome sequences for the missing two permutations: type GCS and type CSG. The searches were conducted similarly to the searches resulting in the type-SGC variants, but we defined patterns that characterize the expected conserved nucleotides and secondary-structure elements for types GCS and CSG (see Materials and Methods). Candidates were again evaluated using covariation as described (23).

**Figure 3.**
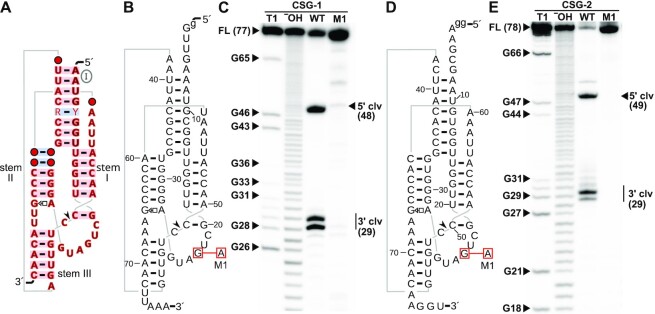
Consensus diagram and co-transcriptional cleavage of type-CSG hammerhead ribozymes. (**A**) Secondary structure and consensus sequence of type-CSG hammerhead ribozymes. Markings are as described in Figures 1 and 2. (**B**) Secondary structure diagram of candidate CSG-1, with annotations as in Figure 2. (**C**) PAGE image showing CSG-1 ribozyme after *in vitro* transcription in the presence of [α-^32^P] ATP for 2 h. The meanings of gel lanes are the same as in Figure 2. Transcription bands for WT (wildtype) ribozyme RNA correspond to the 3′ cleavage product (29 nucleotides) and a 5′ cleavage product of 48 nucleotides. The full-length (FL) transcript of the M1 ribozyme mutant is visible at 77 nucleotides. (**D**) Secondary structure diagram of CSG-2. Annotations are as described for part B. (**E**) PAGE image showing CSG-2 ribozyme after *in vitro* transcription in the presence of [α-^32^P] ATP for 2 h. Transcription bands for WT RNA correspond to the 3′ cleavage product (29 nucleotides) and a 5′ cleavage product of 49 nucleotides. The full-length (FL) transcript of the M1 ribozyme mutant is visible at 78 nt.

These searches revealed a promising alignment composed of two similar sequences that conformed to the theoretical type-CSG structure and that exhibited covariation (Figure 3A, Supplementary Figure S10). No deviations from highly conserved positions (Figure 1A, B) were observed. We validated both candidate sequences (CSG-1 and CSG-2, Figure 3B, D, Supplementary Table S1) in co-transcriptional cleavage assays as described above. Both representatives cleaved at the expected site, and the generated cleavage products correspond to the anticipated sizes of 29 nucleotides for the CSG-1 and CSG-2 3′ fragments and 48 and 49 nucleotides for the CSG-1 and CSG-2 5′ fragments, respectively (Figure 3C, E). The cleavage sites are thus exactly what is expected by structural analogy with hammerhead ribozymes. The addition of untemplated nucleotides during run-off transcription by T7 RNA polymerase results in inhomogeneous 3′ RNA ends in *in vitro* reactions (53,54). Therefore, several bands for 3′ cleavage products can be observed on the gel (Figure 3C, E). While for CSG-2 almost no full-length wildtype ribozyme remains after the 2 h incubation period, a full-length band can still be observed for CSG-1 in addition to strong cleavage bands.

In contrast to type-CSG hammerhead ribozymes, we did not find covariation support for candidate type-GCS hammerhead ribozymes, because we only found one individual sequence that exhibited all expected features (Supplementary Figures S11A and S12). This RNA is found in the Betaproteobacterium *Caballeronia peredens*, upstream of a gene predicted to encode a protein with very modest similarity to eukaryotic beta-2-glycoprotein. We considered this type-GCS sequence to be a weak ribozyme candidate because of the absence of supporting covariation (since only one example is available) and because of its association with a gene for which self-cleaving ribozymes have no known role. Nevertheless, we tested this candidate, termed GCS-1, for self-cleaving ability during *in vitro* transcription (Supplementary Figure S11B), and observed cleavage bands corresponding to the expected sizes of 5′ and 3′ GCS-1 ribozyme cleavage fragments. However, ribozyme cleavage did not approach completion during transcription. Instead, a clearly visible full-length band remained. There is no 5′ cleavage product visible for the cleavage-deficient ribozyme mutant M1. This rules out the possibility that bands generated by transcriptional pausing or premature transcription stop have generated a band pattern that imitates ribozyme cleavage. Thus, the observed cleavage products for the WT-ribozyme likely correspond to site-specific ribozyme cleavage. However, given the lack of covariation, the biological significance of the GCS-1 ribozyme is unclear.

The twister ribozyme is also known for having circularly permuted forms, in a similar manner to those of the hammerhead ribozyme. Twister ribozymes can also be divided into three blocks providing six possible permutations (Supplementary Figure S13). We conducted searches for the missing twister permutations (Supplementary File S1), but did not find promising candidates.

### Kinetic analysis of novel type-SGC, -CSG and -GCS hammerhead ribozymes

Next, we investigated the cleavage kinetics of these newly identified rearranged hammerhead ribozymes. For this, we monitored the formation of ribozyme cleavage products during transcription by withdrawing reaction aliquots at different times and separating the resulting RNA by denaturing PAGE. We used reaction conditions under which the transcription rate remains constant (55) (see Materials and Methods), in which case a simplified two-step reaction can be assumed that consists of ribozyme transcription followed by cleavage. Under these conditions, the fraction of full-length (uncleaved) transcript is independent of the transcription rate, while the rate of accumulation of cleaved transcript depends on both the transcription and cleavage rates (43). Therefore, given a constant transcription rate during the time of analysis, the observed cleavage rate (*k*_obs_) can be determined by plotting the fraction of full-length transcript remaining uncleaved versus time (Methods, Supplementary Figure S14).

We determined cleavage rates for several type-SGC and -CSG examples as well as the type-GCS sequence (Supplementary Figures S15 and S16). We included up to 13 natural nucleotides flanking the predicted ribozyme sequences into our construct design to account for any possible tertiary interactions not detected as part of the RNA motif (Figure 2A–C, Figure 3B, D, Supplementary Figure S11A, Supplementary Table S1). For the SGC-type hammerhead ribozymes, cleavage rates ranged from 0.007 min^−1^ (SGC-2) to 0.13 min^−1^ (SGC-3) (Table 1). The two type-CSG hammerhead ribozymes cleaved with rates of 0.004 and 0.03 min^−1^, while we obtained a *k*_obs_ of 0.01 min^−1^ for the prospective type-GCS hammerhead ribozyme (Table 1).

**Table 1.** Cleavage rates determined for type-SGC, -CSG and -GCS hammerhead ribozyme representatives during *in vitro* transcription (80 mM HEPES, pH 7.5, 25 mM Mg, 37°C, see Materials and Methods for details). ‘Construct’: describes the ribozyme tested. The prefix ‘short’ indicates that no flanking nucleotides were added (see text). ‘*k*_obs_ (min^−1^)’: Cleavage rate. Up to three independent experiments were used to obtain the observed rate constant *k*_obs_ in cleavages per minute (min^−1^). Where applicable, mean values and standard deviations are shown. Values are rounded to no more than two significant figures. ‘*R*^2^’: coefficient of determination (*R*^2^), which indicates the goodness-of-fit to the kinetic model

Construct	*k*_obs_ (min^−1^)	*R*²
SGC-1	0.026 ± 0.002	0.99
shortSGC-1	0.051	0.94
SGC-2	0.007 ± 0.001	0.89
shortSGC-2	0.06	0.99
SGC-3	0.13 ± 0.04	0.98
shortSGC-3	0.19 ± 0.03	0.99
SGC-4	0.032 ± 0.009	0.98
SGC-5	0.084 ± 0.015	0.99
CSG-1	0.004 ± 0.002	0.92
CSG-2	0.026 ± 0.01	0.98
GCS-1	0.014 ± 0.0017	0.95

The extra 13 flanking nucleotides could improve ribozyme cleavage if important structural elements were missed in the determination of the motif. However, if the predicted motif includes all important elements, the flanking nucleotides could compromise folding (56). To investigate this possibility, we also collected cleavage rates for type-SGC representatives that only comprised the conserved core of the motif, i.e. not including stretches of natural flanking sequence. These ‘short’ type-SGC hammerhead ribozymes (shortSGC-1–3) cleaved faster than their extended counterparts (Table 1). This result suggests that we have not missed vital parts of the ribozyme sequence and that the additional RNA sequences increase the chance for alternative ribozyme folds that do not cleave optimally.

To evaluate whether the speeds we determined were in a biologically plausible range, we gathered information on observed rate constants for previously studied natural hammerhead ribozyme sequences. We only considered examples that contain sequence stretches with the stem I–II interaction that stabilize the hammerhead ribozyme core (Figure 1B). Such interactions are important, but were missing from many early studies of hammerhead ribozymes (2,44–46). For these complete ribozymes, previously determined cleavage rates vary greatly (Supplementary Table S3). Due to the use of different reaction conditions in different studies, speed comparisons are not always feasible. For example, if one study used more magnesium (which tends to increase speed) but lower reaction temperatures (which tend to slow cleavage) than another study, it is difficult to compare their cleavage speeds. Despite this technical difficulty, the data summarized in Supplementary Table S3 indicate that a large portion of previously studied hammerhead ribozymes are faster than the type-SGC ribozymes we investigated. It is also clear that at least five previously investigated hammerhead ribozymes cleave more slowly than our newly found permuted ribozymes (57,58). Thus, type-SGC ribozymes cleave within the range of speeds determined for previously known hammerhead ribozyme permutations, and we therefore expect that their speed is sufficient for a biological function.

### Other candidate RNAs discovered

After finding type-SGC hammerhead ribozymes, we conducted a new analysis to find novel structured RNAs within contigs containing either hairpin or the new hammerhead ribozymes. This later analysis allowed us to find additional motifs that were not apparent when using only the hairpin ribozymes. In total, we discovered 26 motifs, including the type-SGC hammerhead ribozymes corresponding to the HRIMA-1 motif. No RNAs were discovered in genomes of known organisms, except for the candidate type-GCS hammerhead ribozyme in *C. peredens*. The previously identified hairpin ribozymes (23) were found exclusively in metatranscriptomes, and were not detected in any DNA form. They were especially common in association with spruce trees, but were also present in a variety of other environments. Almost all of the motifs found in the present work are also exclusive to metatranscriptomes, and most were found in similar environments to those of the hairpin ribozymes (Supplementary Table S2, Supplementary File S4). However, the type-CSG hammerhead ribozymes occur exclusively in metagenomes (i.e. in DNA), and the HRIMA-5 motif is sometimes found in metagenomes. We selected motifs to test experimentally based on a subjective evaluation of their likelihood to function as self-cleaving ribozymes. Previous work proposed that self-cleaving ribozymes are likely to have highly conserved nucleotide positions and elements of complex secondary structure, i.e. multi-stem junctions or pseudoknots (24). It is difficult to evaluate conservation, because the evolutionary distance between the metatranscriptomic sequences containing the various motifs is unknown. We also expected that self-cleaving ribozymes would be between about 50 and 80 nucleotides in length, and would exhibit significant covariation. See also Supplementary Note S2. Apart from hammerhead ribozymes, we ultimately tested ten motifs: HRIMA-2, -3, -4, -5, -6, -7, -8, -9, -15 and -26, and did not observe *in vitro* cleavage.

## DISCUSSION

Using a new, hairpin-ribozyme-based strategy for *de novo* discovery of self-cleaving ribozymes, we found an unexpected non-circular permutation of hammerhead ribozymes. Our bioinformatics and experimental analyses establish that these RNAs are non-circularly-permuted versions of hammerhead ribozymes and presumably exhibit highly similar 3D catalytic cores. First, the patterns of conservation are strikingly similar to those of established hammerhead ribozymes. In particular, all highly conserved nucleotides and stems in the hammerhead ribozyme core (Figure 1A, B) are also highly conserved in the non-circularly-permuted forms (e.g. Figure 1C). Second, mutational experiments establish that all four predicted helices (Figure 1C), including the interaction between stems I and II, are required for cleavage (Figure 2C, E). Third, the helices predicted to fit the hammerhead ribozyme structure (stems I, II and III and the interaction between stems I and II) exhibit copious covariation in type-SGC ribozymes, and modest covariation in type-CSG ribozymes (Figures 1C, 3A, Supplementary Figures S6, S7, S8, S10, Supplementary Table S2). Type-SGC ribozymes have covariation supported by the statistical test implemented in the R-scape software (38) (Figure 1C, Supplementary Figure S7, Supplementary Table S2). Crucially, covariation implies evolutionary pressure to conserve the permuted ribozyme's secondary structure, and thus supports the biological importance of these structures. Fourth, based on the apparent similarity to hammerhead ribozymes, we considered previous experimental work on these ribozymes, and precisely predicted the location of the cleavage site and that the M1 mutation would abolish cleavage. These predictions were confirmed experimentally. Fifth, kinetic studies show that type-SGC ribozymes enhance cleavage by >6 orders of magnitude over background. Although their reactions are slower than average compared to previously studied hammerhead ribozymes, at least several previously characterized ribozymes (57,58) were slower. So, the type-SGC ribozymes are likely fast enough to be biologically relevant. These five points provide strong evidence for the existence of natural non-circular permutations, specifically of hammerhead ribozymes.

The evidence for the biological relevance of type-CSG and -GCS permutations is weaker because of the lesser number of covariation events observed. We believe that the predicted type-CSG ribozymes are likely valid, but more skepticism of the proposed type-GCS ribozyme is warranted because of the absence of covariation support.

For decades after its discovery, the hammerhead ribozyme was thought to require only the region spanning stems I–III (e.g. as depicted in Figure 1A). However, later work demonstrated an important interaction between the loops of stems I and II (2,44–46) (Figure 1B, circled ‘I’), which sometimes takes the form of a helix (47). Predicted type-SGC and -CSG ribozymes appear to have a helix bringing together stems I and II. Additionally, due to the peculiar topology of type-GCS ribozymes, their stem III could potentially also function as a pseudoknot to bring stems I and II together (Supplementary Figure S17). Since the type-SGC and -CSG ribozymes’ cores are highly similar in conservation to those of previously studied hammerhead ribozymes, these cores are compatible with the established hammerhead ribozyme crystal structure (46) (Supplementary Figure S18). Outside the core, the connectivity of type-SGC ribozymes is distinct from those of previously studied hammerhead ribozymes, but in most respects they are clearly compatible (Supplementary Figure S18). The one aspect where compatibility is not obvious arises in a few type-SGC ribozymes, in which the distal end of the interaction between stems I and II (Figure 1C, circled ‘I’) and the distal end of stem III are directly connected, with no intervening nucleotides (Figure 1C, ‘0–44 nt’, Supplementary Figure S18). These two locations are far from each other in the crystal structure, and stems II and III form a coaxial stack (46). Perhaps the two helices’ lengths and relative angles allow these locations to be proximal in 3D. An atomic-resolution structure is likely to reveal important structural insights into this and other questions about how the non-circular permutations are accommodated and we are currently working on solving the crystal structure of the new hammerhead ribozyme permutations.

As noted above, the cleavage rates of type-SGC ribozymes are on the slower end of previously studied hammerhead ribozymes. It is possible that the type-SGC hammerhead ribozymes are naturally in reaction conditions that are significantly more favorable than those we tested, and these conditions could include the presence of a helper protein. It is also conceivable that the permuted structure is inherently not conducive to highly efficient cleavage, which could explain why these permutations are rarer than the previously known types I, II and III. In a biological context, there might not be an advantage to more speed beyond a certain point, and there is often a need to disable the ribozyme (e.g. while rolling-circle replication is not in process). We also cannot rule out the possibility that other type-SGC ribozymes are significantly faster, and we happened to choose slower examples. More research will be needed to address this question, and it would be especially interesting if folding into the non-circular permutation is somehow inherently more difficult.

The newly found permutations raise the question of how these hammerhead ribozymes evolved. Previous studies showed that hammerhead ribozymes are a frequent result of *in vitro* evolution experiments (59). Thus, hammerhead ribozymes may evolve easily, and currently extant natural hammerhead ribozymes might result from convergent evolution (59). A system of non-circular permutations within DNA methyltransferase proteins is broadly similar to what we observe with hammerhead ribozymes, and highly plausible models for the evolution of these permutations were analyzed (5). One model of circular permutations proposed a tandem duplication of the enzyme's gene followed by a new start codon that skips part of the first copy. An analogous model could apply to ribozymes, but would not explain how a non-circular permutation could evolve. Other possibilities would be rearrangements of sub-gene fragments that could result in homologous non-circular permutations (60). Thus, there is potential for non-circularly permuted ribozymes to arise via rearrangements or by convergent evolution.

Apart from hammerhead ribozymes, none of the HRIMA RNA candidates we tested cleaved themselves in our experiments. We see two likely explanations for this result. First, we proposed that the recently discovered hairpin ribozymes occur in virus-like organisms with single-stranded circular RNA genomes (23). Such RNAs presumably contain structural features unrelated to self-cleavage, e.g. to bind host proteins or to inhibit cleavage when not desired. The non-cleaving candidate RNAs we found might thus perform other functions. Second, some candidates might be self-cleaving ribozymes that fail to function under our experimental conditions. Additionally, it is possible that some RNA molecules we tested are too long or short. A lack of variation between some sequences results in a lack of potential covariation, which can mean that it is not always possible to detect the complete structure of a given motif. On the other hand, excess flanking sequence could interfere with ribozyme function. Further study will be needed to determine the functions of the remaining motifs we uncovered in this work, and how they impact the biology of their poorly understood host organisms.

Ribozymes and small-molecule-binding RNAs called aptamers have been extensively used in various biotechnology applications, e.g. as RNA-based environmental sensors or for artificial gene regulation (21,61,62). In many cases, different RNAs are combined, leading to more complex structures. For example, aptamers and ribozymes are often combined to make gene control units (62). In protein-based biotechnology, permuted forms have been exploited to increase the protein's half-life, accelerate enzymatic activity, promote thermostability as well as to study the protein itself (1,63). Circular permutations have also been applied to RNA, and improved binding affinity and signal strength (64). Non-circularly permuted RNAs could thus provide additional flexibility to RNAs used in engineering, leading to improvements along the lines of those in protein engineering, as well as possibly optimizing the order in which components are transcribed.

In RNA structural biology, pseudoknots are considered fundamental blocks of RNA structures (65,66). Accordingly, multiple groups have independently proposed using pseudoknots to classify self-cleaving ribozymes into two structural groups: those formed by helical junctions and those with two or more pseudoknots (67,68). Hammerhead ribozymes have previously been classified as helical junction structures, since the minimal ribozyme consisting of stems I, II and III (Figure 1A, B) does not include a pseudoknot. By contrast, the newly found type-SGC, -GCS and -CSG permuted forms have two pseudoknots in the ribozyme core and do not contain a multi-stem junction (Figure 1G, H). Non-circular permutations can thus create or remove pseudoknots in structurally related RNA molecules. This phenomenon implies that the presence of a pseudoknot might not be an essential characteristic of the structure or function of any particular RNA. Therefore, while pseudoknots clearly remain an important element of RNA structure, a more nuanced understanding of them might be necessary.

Our results raise the question of what RNAs other than hammerhead ribozymes might naturally occur in non-circular permuted forms. Although we did not find strong candidates for twister ribozymes, it seems likely that additional sequence data or alternate search strategies will find unusual natural permutations for these or other RNA structural classes.

## DATA AVAILABILITY

All relevant data are available in supplementary materials, which are also available on Zenodo at https://doi.org/10.5281/zenodo.7382572. Alignments from papers of the Z.W. group accepted for publication are also freely available in the ZWD repository (https://bitbucket.org/zashaw/zashaweinbergdata/src/master).

## Supplementary Material

gkad137_Supplemental_Files

## Notes

Present address: Richard Friedrich, AlphaOmega Labor, Messeallee 23, 04158 Leipzig, Germany.
