# Supplementary material for: Discovery of natural non-circular permutations in non-coding RNAs
Source: Nucleic Acids Res. 2023 Mar 13;51(6):2850–61. doi: 10.1093/nar/gkad137 (PMC10085705; doi:10.1093/nar/gkad137)
Supplement: gkad137_Supplemental_Files [file gkad137_supplemental_files.zip › 2023-02-07-supplementary.pdf]

**Supplementary File descriptions, Notes, Tables and Figures:
Discovery of natural non-circular permutations in non-coding RNAs**

Iris Eckert[1], Richard Friedrich[2,3], Christina E. Weinberg[2,*], Zasha Weinberg[1,*]

Supplementary File descriptions

Supplementary File 1. RNAMotif / DARN! search patterns for the new permutations of the hammerhead ribozyme and analogous twister ribozyme permutations.

Supplementary File 2. Alignments of novel RNA motifs detected in this work (Supplementary Table 2). The alignments are stored in Stockholm format, which is a text format that can be viewed with a fixed-width font and can also be interpreted by several computer programs. Only unique sequences are supplied.

Supplementary File 3. Printable alignments of novel RNA motifs detected in this work (Supplementary Table 2), in PDF format. Only unique sequences are shown.

Supplementary File 4. Frequencies of RNA motifs in different environmental datasets. This data is a basis for relevant columns in Supplementary Table 2. The first worksheet describes the data format.

Supplementary Notes

Supplementary Note 1: Since all of our data on rearranged hammerhead ribozymes are derived from assembled metatranscriptomes, one might wonder if the apparent rearrangements are caused by falsely assembled sequences. We can rule this possibility out. First, most of our sequences come from spruce-associated metatranscriptomes with read lengths of 151 nucleotides. The longest rearranged hammerhead ribozyme we predicted was 104 nucleotides, and many fit entirely within a single read. Moreover, we previously used RT-PCR and sequencing to analyze an RNA molecule based on a metatranscriptome that was predicted to contain a hairpin ribozyme (1). The sequence we determined, based on Sanger sequencing, perfectly matched the assembled metatranscriptome contig (1). This contig, denoted Ga0247519_111615, also contains a type-SGC hammerhead ribozyme on its reverse-complement strand. Thus, this type-SGC ribozyme was also validated by Sanger sequencing.

Supplementary Note 2: We also used strand information to select motifs to test for self-cleavage. Our hypothesis assumes organisms present in metatranscriptomes use self-cleaving ribozymes for rolling-circle replication. So, there is no clear need for more than one self-cleaving ribozyme in the same strand orientation, at least for motifs occurring in metatranscriptomes. Therefore, we generally did not test motifs that are predicted to be in the same strand as another known self-cleaving ribozyme (Supplementary Table 2). We also did not work on motifs when it was immediately clear that they had this property. We predicted the strand on which the putative RNA structure functions mainly using the ratio of A-C or C-A versus G-U or U-G base pairs and using RNAstrand (2) version 2.0.0, although both automated and manual strand prediction is not always reliable.

Supplementary Tables

Supplementary Table 1: Primers and oligonucleotides used in this study. Notation like "Ga0247515_120448/486-401" indicates a specific region of nucleotides on a sequence contig. In this example, Ga0247515_120448 is the sequence accession, 486 is the location of the 5′ nucleotide in the range and 401 is the location of the 3′ nucleotide. Because 486>401, the region is on the reverse-complement of the sequence contig. The sequence accession NZ_FCOH02000029.1 refers to the RefSeq nucleotide database, available on the NCBI website. All other accessions are available from IMG/M.

Name	Sequence 5′ to 3′	Purpose
**CEW164**	GAAATTAATACGACTCACTATAggAACTTAAACTGTCATGTTTCTGGACCGAAACAGTAC	Oligo to create an in vitro transcription template of SGC-2 from Ga0247515_120448/486-401 by overlap extension with a partly complementary reverse oligo. This forward oligo is compatible with the wt and RB1 reverse oligo and it contains a T7 promotor followed by two G nucleotides that are not part of the natural sequence (gg).
**CEW165**	TATGAGTTGCGCATGGCGGACCATCATCAGATGTTCCACGCGCGTACTGTTTCGGTCCAG	Oligo to create an in vitro transcription template of SGC-2 from Ga0247515_120448/486-401 by overlap extension with a partly complementary forward oligo.
**CEW166**	TATGAGTTGCGCATGGCGGACCATCATTAGATGTTCCACGCGCGTACTGTTTCGGTCCAG	Oligo to create an in vitro transcription template of SGC-2 RB1 from by overlap extension with a partly complementary forward oligo.
**CEW221**	GAAATTAATACGACTCACTATAggACACACTACTGTCATGTTTCAGGACCGAAACAGTGG	Oligo to create an in vitro transcription template of SGC-1 from Ga0153880_1354682/189-107 by overlap extension with a partly complementary reverse oligo. This forward oligo is compatible with the wt and RB1 reverse oligo and it contains a T7 promotor followed by two G nucleotides that are not part of the natural sequence (gg).
**CEW222**	ATGGCAGCAAAGAGAGCGGACCATCATCAGATGTTCTCTCTCCACTGTTTCGGTCCTG	Oligo to create an in vitro transcription template of SGC-1 from Ga0153880_1354682/189-107 by overlap extension with a partly complementary forward oligo.
**CEW223**	ATGGCAGCAAAGAGAGCGGACCATCATTAGATGTTCTCTCTCCACTGTTTCGGTCCTG	Oligo to create an in vitro transcription template of SGC-1 RB1 from Ga0153880_1354682/189-107 by overlap extension with a partly complementary forward oligo.
**CEW256**	CTATGAGGCGGTTCGGTAACCCAAGGGCCAACCGAATAACCAAAAAGGGGGTGTATGGGG	Forward oligo to create PCR template of HRIMA 26 from Ga0247520_118066/451-360 by overlap extension with a partly complementary reverse oligo CEW257. Forward primer with T7 promoter and reverse primers are needed to create transcription template.
**CEW257**	ATCACGGACGGCGGGGTTAGTGTCTCGCTCCTCTAGAAGGAGCCCCATACACCCCCTT	Reverse oligo to create a PCR template of HRIMA 26 from Ga0247520_118066/451-360 by overlap extension with a partly complementary forward oligo CEW256. Forward primer with T7 promoter and reverse primers are needed to create transcription template.

**CEW258**	CTATGAGGCGGTTCGGTAAC	Forward primer to create in vitro transcription template of HRIMA 26 from Ga0247520_118066/451-360 by PCR with reverse primers (dependent on length of template, CEW259-261). Forward primer with T7 promoter and reverse primers are needed to create transcription template.
**CEW259**	ATCACGGACGGCGGG	Reverse primer to create short in vitro transcription template of HRIMA 26 from Ga0247520_118066/451-360 by PCR with forward primer CEW258.
**CEW260**	CCTCGGAGAATCTTCGCTCTGTATCACGGACGGCGGG	Reverse primer to create extended in vitro transcription template of HRIMA 26 from Ga0247520_118066/451-360 by PCR with forward primer CEW258.
**CEW261**	GGATTTCGAACGAGATGTTTCTGCCTCGGAGAATCTTCGCTCTGTATCACGGACGGCGGG	Reverse primer to create very extended in vitro transcription template of HRIMA 26 from Ga0247520_118066/451-360 by PCR with forward primer CEW258.
**CEW262**	CTCTAAAGTGGAATGTCGCTATACTGCGGGCTATCCCCTCTGTTGAGGGGGAGTAAC	Forward oligo to create PCR template of HRIMA 3 from Ga0247519_120287/30-114 by overlap extension with a partly complementary reverse oligo CEW263. Forward primer with T7 promoter and reverse primers are needed to create transcription template.
**CEW263**	TCTACAAGTGGAGCCATACTGCTTTCCAGGCTTGAGTTGTTACTCCCCCTCAACAGAG	Reverse oligo to create a PCR template of HRIMA 3 from Ga0247519_120287/30-114 by overlap extension with a partly complementary forward oligo CEW262. Forward primer with T7 promoter and reverse primers are needed to create transcription template.
**CEW264**	GAAATTAATACGACTCACTATAgCTCTAAAGTGGAATGTCGC	Forward primer to create in vitro transcription template of HRIMA 3 from Ga0247519_120287/30-114 by PCR with reverse primer CEW265. Only one additional g is added to initiate T7 transcription, because otherwise a complementary sequence to 3'P5 is created.
**CEW265**	TCTACAAGTGGAGCCATAC	Reverse primer to create in vitro transcription template of HRIMA 3 from Ga0247519_120287/30-114 by PCR with forward primer CEW264.
**CEW266**	TCTACAAGTGGAGCCATAC	Reverse primer to create in vitro transcription template of HRIMA 3 from Ga0247519_120287/30-114 by PCR with forward primer CEW264.
**CEW266**	GAAATTAATACGACTCACTATAggTAAACCTGCATGCTTAAGTGCATCACGTTCTCGAAC	Forward oligo to create in vitro transcription template of HRIMA 15 from Ga0257136_1059838/150-206 by overlap extension with a partly complementary reverse oligo CEW267.
**CEW267**	GCCACCCGCCCGCACATATACGGCGTAGAACGTTCGAGAACGTGATGCAC	Reverse oligo to create in vitro transcription template of HRIMA 15 from Ga0257136_1059838/150-206 by overlap extension with a partly complementary forward oligo CEW266.

**CEW268**	GAGCAAAGGAGTCTTCAGGGACTGCCTGTTCAGTATCTGGTTATTTCGGGAAAACTTAGC	Forward oligo to create PCR template of HRIMA 2 from Ga0179955_1089777/325-432 by overlap extension with a partly complementary reverse oligo CEW269. Forward primer with T7 promoter and reverse primers are needed to create transcription template.
**CEW269**	GAATGGAGCGATGATCAGATAGGCCTACGGGTGAGAGGCTAAGTTTTCCCGAAATAACC	Reverse oligo to create PCR template of HRIMA 2 from Ga0179955_1089777/325-432 by overlap extension with a partly complementary forward oligo CEW268. Forward primer with T7 promoter and reverse primers are needed to create transcription template.
**CEW270**	GAAATTAATACGACTCACTATAGGAGCAAAGGAGTCTTCAGG	Forward primer to create in vitro transcription template of HRIMA 2 from Ga0179955_1089777/325-432 by PCR with reverse primer CEW271.
**CEW271**	GAATGGAGCGATGATCAGATAG	Reverse primer to create in vitro transcription template of HRIMA 2 from Ga0179955_1089777/325-432 by PCR with forward primer CEW270.
**CEW298**	GAAATTAATACGACTCACTATAGGACGCGGAGACCGAAACGCACTGTTGCGCGATC	Oligo to create an *in vitro* transcription template of GCS-1 from NZ_FCOH02000029.1/62359-62449 by overlap extension with a partly complementary reverse oligo. This forward oligo contains a T7 promotor followed by two natural G nucleotides.
**CEW299**	CGCGGCGCACACAGCGCAACGACGCACTGGACCTCATCAGATCGCGCAACAGTGCG	Oligo to create an in vitro transcription template of GCS-1 from NZ_FCOH02000029.1/62359-62449 by overlap extension with a partly complementary forward oligo.
**CEW300**	CGCGGCGCACACAGCGCAACGACGCACTGGACCTCATTAGATCGCGCAACAGTGCG	Oligo to create an in vitro transcription template of GCS-1 Mutant RB1 from NZ_FCOH02000029.1/62359-62449 by overlap extension with a partly complementary forward oligo.
**CEW306**	GAAATTAATACGACTCACTATAgGTTGAAATGCGGTTGGTTGCTGATGAGGGGTGCCGC	Oligo to create an *in vitro* transcription template of CSG-1 from Ga0102961_1534289/214-370 by overlap extension with a partly complementary reverse oligo. This forward oligo contains a T7 promotor followed by one additional g nucleotide for enhanced transcription initiation.
**CEW307**	TTTAAGTTGTTTCGGGGTATTAATGGTTGGACAACTTAATGCGGCACCCCTCATC	Oligo to create an in vitro transcription template of CGS-1 from Ga0102961_1534289/214-370 by overlap extension with a partly complementary forward oligo.
**CEW308**	TTGAAATGCGGTTGGTTGCTAATGAGGGGTGCCGC	Oligo to create an in vitro transcription template of CSG-1 Mutant RB1 from Ga0102961_1534289/214-370 by overlap extension with a partly complementary reverse oligo. This forward oligo contains a T7 promotor followed by one additional g nucleotide for enhanced transcription initiation.
**CEW309**	TTTAAGTTGTTTCGGGGTATTAATGGTTGGACAACTTAATGCGGCACCCCTCATT	Oligo to create an in vitro transcription template of CSG-1 Mutant RB1 from Ga0102961_1534289/214-370 by overlap extension with a partly complementary forward oligo.

**CEW310**	GAAATTAATACGACTCACTATAggAAGCGAATGTGGTTGGTTGCTGATGAGGGTGGCCAC	Oligo to create an in vitro transcription template of CSG-2 from Ga0102955_1556782/362-197 by overlap extension with a partly complementary reverse oligo. This forward oligo contains a T7 promotor followed by two synthetic g nucleotides.
**CEW311**	ACCTTGTTGTTTCGGGTGTTTAATGGTTGGACAACTGAATGTGGCCACCCTCAT	Oligo to create an in vitro transcription template of CSG-2 from Ga0102955_1556782/362-197 by overlap extension with a partly complementary forward oligo.
**CEW312**	GAAATTAATACGACTCACTATAggAAGCGAATGTGGTTGGTTGCTAATGAGGGTGGCCAC	Oligo to create an in vitro transcription template of CSG-2 Mutant RB1 from Ga0102955_1556782/362-197 by overlap extension with a partly complementary forward oligo.
**CEW333**	AAATTAATACGACTCACTATAggAAAAATAACAGCAATCTGTGATTAGTGTCGAAATTGC	Oligo to create an *in vitro* transcription template of SGC-3 from Ga0247524_116441/281-201 by overlap extension with a partly complementary reverse oligo. This forward oligo is compatible with the wt and RB1 reverse oligo and it contains a T7 promotor followed by two G nucleotides that are not part of the natural sequence (gg).
**CEW334**	TTCAATGTTACGTGTTGTGTCATCATCAGTATGATCACGTAAGCAATTTCGACACTAATC	Oligo to create an in vitro transcription template of SGC-3 from Ga0247524_116441/281-201 by overlap extension with a partly complementary forward oligo.
**CEW335**	TTCAATGTTACGTGTTGTGTCATCATTAGTATGATCACGTAAGCAATTTCGACACTAATC	Oligo to create an in vitro transcription template of SGC-3 RB1 from Ga0247524_116441/281-201 from by overlap extension with a partly complementary forward oligo.
**CEW348**	GCTTCTCCGAGCCATGGATACGGCCAGAGACTGCATAGCAGTCTGGACAAATCCTG	De novo generation of transcription template of HRIMA-4 sequence Ga0247530_109035/125-228 for ribozyme discovery analysis. Use with CEW349 for template generation.
**CEW349**	CAGAGCACCACTGAATTACTCCAGTGACACCTGCAGGATTTGTCCAGACTGC	De novo generation of transcription template of HRIMA-4 sequence Ga0247530_109035/125-228 for ribozyme discovery analysis. Use with CEW348 for template generation.
**CEW350**	GAAATTAATACGACTCACTATAgggTCATGCGCTTCTCCGAGCCATGG	De novo generation of transcription template of HRIMA-4 sequence Ga0247530_109035/125-228 for ribozyme discovery analysis. T7 promotor addition of product from overlap extension with CEW348/349. Use with CEW351 for template generation.
**CEW351**	TCGGCTGGACACAGGTCGTTGTACGACTCCAGAGCACCACTGAATTACTCC	De novo generation of transcription template of HRIMA-4 sequence Ga0247530_109035/125-228 for ribozyme discovery analysis. 3´elongation of product from overlap extension with CEW348/349. Use with CEW350 for template generation.
**CEW353**	CGCTTCAACTTTCCGGAAGTAATGCACCGGATTGAGTAAAGTTTCTGCGTTGGATGTAA	De novo generation of transcription template of HRIMA-5 sequence Ga0247551_100244/228-18 for ribozyme discovery analysis. Use with CEW354 for template generation.

**CEW354**	CGCGGGGTTTAAACTACATTGCTTACATCCAACGCAGAAACTTTACTC	De novo generation of transcription template of HRIMA-5 sequence Ga0247551_100244/228-18 for ribozyme discovery analysis. Use with CEW353 for template generation.
**CEW355**	GAAATTAATACGACTCACTATAgggTGTGCCACAAGACGCTTCAACTTTCCGGAAGTAA	De novo generation of transcription template of HRIMA-5 sequence Ga0247551_100244/228-18 for ribozyme discovery analysis. Use with CEW356 for template generation by PCR.
**CEW356**	CAGCCAAACAGACGGTACTTGTTTCTTGGACATGCTGACGCGGGGTTTAAACTACATTG	De novo generation of transcription template of HRIMA-5 sequence Ga0247551_100244/228-18 for ribozyme discovery analysis. Use with CEW355 on 948-945_core for template generation by PCR.
**CEW357**	CGCGTTGATACTAAGTTAGGAAAGTAGCTCATGGAGCTCAGACGCCTCTAAGGCGAACC	De novo generation of transcription template of HRIMA-6 sequence Ga0247512_108804/831-934 for ribozyme discovery analysis. Use with CEW358 for template generation.
**CEW358**	TGAGTACGTTAGGGAGTGCTCCGTGTCTCGGTTCGCCTTAGAGGCG	De novo generation of transcription template of HRIMA-6 sequence Ga0247512_108804/831-934 for ribozyme discovery analysis. Use with CEW357 for template generation.
**CEW359**	GAAATTAATACGACTCACTATAgggCCTATCGCGTTGATACTAAGTTAGGAAAG	De novo generation of transcription template of HRIMA-6 sequence Ga0247512_108804/831-934 for ribozyme discovery analysis. Use with CEW360 on 948-2675_core for template generation by PCR.
**CEW360**	GACGTCAGCAGGAGAGTTGACACGTGAGTACGTTAGGGAGTGCTCC	De novo generation of transcription template of HRIMA-6 sequence Ga0247512_108804/831-934 for ribozyme discovery analysis. Use with CEW359 on 948-2675_core for template generation by PCR.
**CEW361**	GAAATTAATACGACTCACTATAgggCCCTGGAACGGCAACGAGTTGCGCGCCCCGAAAGG	De novo generation of transcription template of HRIMA-7 sequence Ga0265745_1000512/608-688 for ribozyme discovery analysis. Use with CEW362 for template generation by PCR.
**CEW362**	ACCCAGACACACAACTCATGGCTTTCGCCTTCTGTGCGCGACAAACCCTTTCGGGGCGCG	De novo generation of transcription template of HRIMA-7 sequence Ga0265745_1000512/608-688 for ribozyme discovery analysis. Use with CEW361 for template generation by PCR.
**CEW363**	GAAATTAATACGACTCACTATAgggTTTAAAGTAAGCCCTCCCAGTTACG	De novo generation of transcription template of HRIMA-8 sequence Ga0247524_101842/1050-1110 for ribozyme discovery analysis. Use with CEW364 for template generation.
**CEW364**	TGATTGGGACGCCCGCCTATGAAAAACCAGTCAATGCGTAACTGGGAGGGCTTAC	De novo generation of transcription template of HRIMA-8 sequence Ga0247524_101842/1050-1110 for ribozyme discovery analysis. Use with CEW363 for template generation.
**CEW365**	GTGTCTTCTTGTTCTTGTTCCCGGTCCGAAAGGATGCAGGGAGGATGAGATCTCACCTA	De novo generation of transcription template of HRIMA-9 sequence Ga0265745_1000046/926-1015 for ribozyme

		discovery analysis. Use with CEW366 for template generation.
**CEW366**	CGCTACCCATTCCTAGGTGA GATCTCATCCTCCCT	De novo generation of transcription template of HRIMA-9 sequence Ga0265745_1000046/926-1015 for ribozyme discovery analysis. Use with CEW365 for template generation.
**CEW367**	GAAATTAATACGACTCACTA TAgggTTCTTCGGACGGTGT CTTCTTGTTCTTGTTCCCG	De novo generation of transcription template of HRIMA-9 sequence Ga0265745_1000046/926-1015 for ribozyme discovery analysis. Use with CEW368 on 948-134_core for template generation by PCR.
**CEW368**	AACCCTCTTGATAGCAGTCG GCTGACCGCTACCCATTCCT AGGTG	De novo generation of transcription template of HRIMA-9 sequence Ga0265745_1000046/926-1015 for ribozyme discovery analysis. Use with CEW367 on 948-134_core for template generation by PCR.
**CEW659**	GAAATTAATACGACTCACTA TAgggTACTGTCATGTTTCAG GACCGAAAC	Oligo to create an in vitro transcription template for hammerhead ribozyme construct shortSGC-1 by overlap extension with a partly complementary reverse oligo (CEW660). This forward oligo contains a T7 promotor followed by three G nucleotides that are not part of the natural sequence (ggg). This construct lacks additional sequence at 5′ and 3′ end in comparison to full-length construct SGC-1.
**CEW660**	AGAGAGCGGACCATCATCA GATGTTCTCTCTCCACTGTTT CGGTCCTGAAA	Oligo to create an in vitro transcription template for hammerhead ribozyme construct shortSGC-1 by overlap extension with a partly complementary forward oligo (CEW659). This construct lacks additional sequence at 5′ and 3′ end in comparison to full-length construct.
**CEW670**	GAAATTAATACGACTCACTA TAgggACTGTCATGTTTCTGG ACCGAAAC	Oligo to create an in vitro transcription template for hammerhead ribozyme construct shortSGC-2 by overlap extension with a partly complementary reverse oligo (CEW671). This forward oligo contains a T7 promotor followed by three G nucleotides that are not part of the natural sequence (ggg). This construct lacks additional sequence at 5′ and 3′ end in comparison to full-length construct SGC-2.
**CEW671**	GCGCATGGCGGACCATCAT CAGATGTTCCACGCGCGTAC TGTTTCGGTCCAGAAA	Oligo to create an in vitro transcription template for hammerhead ribozyme construct shortSGC-2 by overlap extension with a partly complementary forward oligo (CEW670). This construct lacks additional sequence at 5′ and 3′ end in comparison to full-length construct.
**CEW672**	GCGCATGGCGGACCATCATT AGATGTTCCACGCGCGTACT GTTTCGGTCCAGAAA	Oligo to create an in vitro transcription template for red base mutant 1 of hammerhead ribozyme construct shortSGC-2 by overlap extension with a partly complementary forward oligo (CEW670). This construct lacks additional sequence at 5′ and 3′ end in comparison to full-length construct.

**CEW673**	GAAATTAATACGACTCACTATAggGCAATCTGTGATTAGTGTCGAAATTGCTTACG	Oligo to create an in vitro transcription template for hammerhead ribozyme construct shortSGC-3 by overlap extension with a partly complementary reverse oligo (CEW674). This forward oligo contains a T7 promotor followed by two G nucleotides that are not part of the natural sequence (gg). This construct lacks additional sequence at 5' and 3' end in comparison to full-length construct SGC-3.
**CEW674**	TTACGTGTTGTGTCATCATCAGTATGATCACGTAAGCAATTTCGAC	Oligo to create an in vitro transcription template for hammerhead ribozyme variant shortSGC-3 by overlap extension with a partly complementary forward oligo (CEW673). This construct lacks additional sequence at 5' and 3' end in comparison to full-length construct.
**CEW702**	GAAATTAATACGACTCACTATAggATTCAAACTGAACTGTCATGTTTCATTCCCGAAACAGTTACGTACGCATTCCAAG	Oligo to create an in vitro transcription template for hammerhead candidate SGC-4 from Ga0316047_103780/340-253 by overlap extension with a partly complementary reverse oligo (CEW703). This forward oligo contains a T7 promotor followed by two G nucleotides that are not part of the natural sequence (g).
**CEW703**	ACTACAATACCAAGCTTCCCATCATCAGATGTTCTTGGAATGCGTACGTAACTG	Oligo to create an in vitro transcription template for hammerhead candidate SGC-4 from Ga0316047_103780/340-253 by overlap extension with a partly complementary foward oligo (CEW702).
**CEW706**	GAAATTAATACGACTCACTATAggATTCACACACTACTGTCATGTTTCAAGGATCGAAAC	Oligo to create an in vitro transcription template for hammerhead candidate SGC-5 from Ga0184593_130281/355-276 by overlap extension with a partly complementary reverse oligo (CEW707). This forward oligo contains a T7 promotor followed by two G nucleotides that are not part of the natural sequence (g).
**CEW707**	CACAGCAGACAGAGGCGGATCATCATCAGATGTTTCCTCTTACTGTTTCGATCCTTGAAACATG	Oligo to create an in vitro transcription template for hammerhead candidate SGC-5 from Ga0184593_130281/355-276 by overlap extension with a partly complementary foward oligo (CEW706).
**CEW928**	AAATTAATACGACTCACTATAggAAAAATAACAGCAATCTGTGATTAGTGTCGAAATCAC	Oligo to create in vitro transcription template of SGC-3_M2 from Ga0247524_116441/281-201 by overlap extension with a partly complementary reverse oligo. This forward oligo is compatible with the M2 reverse oligo and it contains a T7 promotor followed by two G nucleotides that are not part of the natural sequence (gg). The M2 mutation disrupts stem III.
**CEW929**	TTCAATGTTACGTGTTGTGTCATCATCAGTATGATCACGTAAGTGATTTCGACACTAATC	Oligo to create in vitro transcription template of SGC-3_M2 from Ga0247524_116441/281-201 by overlap extension with a partly complementary foward oligo (CEW928). This oligo is compatible with the CEW928 and CEW930.
**CEW930**	AAATTAATACGACTCACTATAggAAAAATAACAGTGATCTGTGATTAGTGTCGAAATCAC	Oligo to create in vitro transcription template of SGC-3_M3 from Ga0247524_116441/281-201 by overlap extension with a partly complementary reverse oligo (CEW929). This

		forward oligo is compatible with the M2 reverse oligo and it contains a T7 promotor followed by two G nucleotides that are not part of the natural sequence (gg). The M3 mutation is a compensatory mutation to reconstruct stem III.
**CEW931**	AAATTAATACGACTCACTAT AggAAAAATAACAGCAATCA CAGATTAGTGTCGAAATTGC	Oligo to create in vitro transcription template of SGC-3_M4 from Ga0247524_116441/281-201 by overlap extension with a partly complementary reverse oligo. This forward oligo is compatible with the M5 reverse oligo (CEW932) and CEW334 and it contains a T7 promotor followed by two G nucleotides that are not part of the natural sequence (gg). The M4 mutation disrupts stem I.
**CEW932**	TTCAATGTTACGTGTTGTGT CATCATCAGACAGATCACGT AAGCAATTTCGACACTAATC	Oligo to create in vitro transcription template of SGC-3_M5 from Ga0247524_116441/281-201 by overlap extension with a partly complementary foward oligo (CEW931). The M5 mutant is a compensatory mutation to reconstruct stem I.
**CEW933**	AAATTAATACGACTCACTAT AggAAAAATAACAGCAATCT GTGATTAGTACCGAAATTGC	Oligo to create in vitro transcription template of SGC-3_M6 from Ga0247524_116441/281-201 by overlap extension with a partly complementary reverse oligo. This forward oligo is compatible with the M6 (CEW934) and M7 (CEW935) reverse oligo and it contains a T7 promotor followed by two G nucleotides that are not part of the natural sequence (gg). The M6 mutation disrupts stem II.
**CEW934**	TTCAATGTTACGTGTTGTGT CATCATCAGTATGATCACGT AAGCAATTTCGGTACTAATC	Oligo to create in vitro transcription template of SGC-3_M6 from Ga0247524_116441/281-201 by overlap extension with a partly complementary foward oligo (CEW933).
**CEW935**	TTCAATGTTACGTGTTGTAC CATCATCAGTATGATCACGT AAGCAATTTCGGTACTAATC	Oligo to create in vitro transcription template of SGC-3_M7 from Ga0247524_116441/281-201 by overlap extension with a partly complementary foward oligo (CEW933). M7 mutation is a compensatory mutation of stem II.
**CEW936**	TTCAATGTTGTGTGTTGTGT CATCATCAGTATGATCACGT AAGCAATTTCGACACTAATC	Oligo to create in vitro transcription template of SGC-3_M8 from Ga0247524_116441/281-201 by overlap extension with a partly complementary foward oligo CEW333. M8 mutant disrupts the pseudoknot between stem I and II.
**CEW937**	TTCAATGTTGTGTGTTGTGT CATCATCAGTATGATCACAC AAGCAATTTCGACACTAATC	Oligo to create in vitro transcription template of SGC-3_M9 from Ga0247524_116441/281-201 by overlap extension with a partly complementary foward oligo CEW333. M9 mutant compensates for the M8 mutation in the pseudoknot between stem I and II.

Supplementary Table 2. Summary of novel candidate RNA structures determined in this work. “Name”: our name for the motif. Names starting with “Type” refer to novel hammerhead ribozyme permutations. “Type-SGC / HRIMA-1” refers to the type-SGC ribozymes originally detected as the HRIMA-1 motif (Figure 1C). “Type-SGC variant” refers to the second version of type-SGC hammerhead ribozymes that incorporate different nucleotides in stem I (Figure 2C, Supplementary Figure 7, 8). “Motif rating”: a subjective evaluation of the likelihood that the RNA corresponds to a biological RNA (not necessarily a self-cleaving ribozyme). ‘Y’: stronger evidence (including experimental evidence), ‘y’: less strong evidence. “?”: only one active type-GCS hammerhead ribozyme was found, so covariation analysis is not possible. “Cleaves?” : indicates results of experiments. “Y” : experimental evidence of cleavage. “N” : the motif was tested, but no cleavage was observed. Blank cells indicate that no experiment was performed yet. “Number of sequences”: number of sequences that we predict belong to each motif (including identical subsequences from different contigs). “Average length”: average length of all sequences contained in the motif alignment. “*”: HRIMA-10 and -18 often occur in the same sequence contigs as each other, as do HRIMA-14 and -16. It is possible that these pairs of motifs belong to the same larger structure. However, the average distance between the motifs is ~1400 nucleotides for HRIMA-14/-16 and ~500 nucleotides for HRIMA-10/-18. “Number of base pairs”: statistics related to the number of alignment columns predicted to contain base pairs. Note that only some of these proposed base pairs are shown in the consensus diagrams, because not all positions are shown. “Total” (first sub-column): total number of consensus base pairs. “R2R” (second sub-column): number of base pairs annotated as covarying by R2R, but not by R-scape. R2R annotations are, unlike R-scape, not statistically well-founded. However, they suggest possible locations of covariation. Multiple base pairs satisfying R2R’s covariation could suggest support for a structure, even if no individual base pair exhibits statistically significant covariation (i.e., is not annotated by R-scape). “R-scape” (third sub-column): number of base pairs classified by R-scape as covarying, at its standard threshold for statistical significance. “Known ribozyme on”: indicates known ribozymes predicted within the same contig on the same or reverse-complement strand, in at least some cases. The type-CSG and -GCS sequences were discovered by pattern searches and are not associated with hairpin ribozymes or other ribozymes. “HP”: hairpin ribozyme. “HH”: hammerhead ribozyme. “twister”: twister ribozyme. “RNAStrand disagrees?”: indicates motifs for which RNAstrand predicted that the relevant RNA sequence is the reverse-complement of our prediction. For these motifs, there is extra reason for uncertainty about the correct orientation of the RNA. “Habitat”: most-common environments in which the motif occurs. “Spruce” refers to any spruce-tree-associated environment (e.g., spruce tree litter or spruce rhizosphere). “Molecule”: indicates whether the motif is present in sequences derived from RNA (metatranscriptomic), DNA (genomic or metagenomic) or both.

**Name**	**Motif rating**	**Cleaves?**	**Number of sequences**	**Average length**	**Number of base pairs**			**Known ribozyme on**		**RNAstrand disagrees?**	**Habitat**	**Molecule**
					**Total**	**R2R**	**R-scape**	**same strand**	**opposite strand**			
Type-SGC/ HRIMA-1	Y	Y	407	65	47	7	16		HP		soil<br>spruce<br>sludge	RNA

HRIMA-2	Y	N	20	101	22	6	5	HH			sludge	RNA
HRIMA-3	Y	N	47	96	32	6	19	HP	HH		soil<br>spruce	RNA
HRIMA-4	Y	N	78	104	34	14	0		HP		soil<br>spruce	RNA
HRIMA-5	Y	N	35	109	22	10	1		HP		soil<br>spruce<br>freshwater	RNA (soil)<br>DNA (fresh water)
HRIMA-6	Y	N	78	85	26	7	4		HP	yes	soil<br>spruce	RNA
HRIMA-7	Y	N	36	60	17	4	3		HP		soil	RNA
HRIMA-8	Y	N	171	40	11	2	5		HP		soil<br>spruce	RNA
HRIMA-9	Y	N	29	87	32	13	3	HP			soil	RNA
HRIMA-10	Y		59	*70	18	3	7		HP		soil<br>spruce	RNA
HRIMA-11	Y		70	83	30	10	9	HP	HH		soil<br>spruce	RNA
HRIMA-12	Y		138	102	24	6	0	HP			soil<br>spruce	RNA
HRIMA-13	Y		70	104	26	9	3		HP		soil<br>spruce	RNA
HRIMA-14	Y		33	*113	40	19	0		HP		soil	RNA
HRIMA-15	y	N	12	66	17	4	2	twister	HP		saline water<br>fresh water	RNA
HRIMA-16	y		14	*59	18	2	0	HP			soil	RNA
HRIMA-17	y		5	90	35	3	1		HP		sludge	RNA
HRIMA-18	y		120	*51	12	3	5		HP		soil<br>spruce	RNA
HRIMA-19	y		53	74	15	0	13	HP, HH			freshwater<br>sludge<br>sediment<br>soil	RNA
HRIMA-20	y		11	111	37	10	3		HP		spruce<br>soil	RNA

HRIMA-21	y		69	104	10	3	0		HP		soil<br>spruce	RNA
HRIMA-22	y		104	75	19	7	1	HP		yes	soil<br>spruce	RNA
HRIMA-23	y		32	108	33	7	2		HP		soil	RNA
HRIMA-24	y		20	88	16	2	2	HP			fresh water	RNA
HRIMA-25	y		95	74	24	5	0		HP		soil<br>spruce	RNA
HRIMA-26	Y	N	74	85	21	6	1		HP		soil<br>spruce	RNA
Type-SGC-variant	Y	Y	176	61	51	10	20		HP		soil<br>spruce<br>freshwater	RNA
Type-CSG	Y	Y	5	66	25	3	0				soil	DNA
Type-GCS	?	Y	1	51	19	0	0				soil (Beta-proteo-bacterium *Caballeronia peredens*)	DNA

Supplementary Table 3: Literature summary of natural hammerhead ribozyme cleavage rates. Ribozymes listed here are capable of forming auxiliary tertiary interactions (3–5) for proper folding and cleavage. “Reference”: publication from which the ribozyme rates are taken. “Format”: the assay format that was used to determine the cleavage rates. There are three different formats listed: “cis (protein-free)” refers to an assay in which the full-length uncleaved ribozyme RNA is isolated from a denaturing polyacrylamide-gel after transcription. If ribozymes cleaved extensively during transcription, the reactions were performed in the presence of a “blocking oligo” that inhibited self-cleavage. After PAGE-purification, the full-length RNA is available for cleavage assays under controlled, protein-free conditions. “cis (co-tr)” refers to the determination of ribozyme cleavage rates during *in vitro* transcription (6). “trans” refers to the determination of ribozyme cleavage rates using a bimolecular construct. Here, the ribozyme is divided into an enzyme strand that comprises the majority of the ribozyme sequence and a substrate strand that harbors the cleavage site. Only when both strands hybridize does cleavage occur. “Conditions” column lists the parameters under which the cleavage assays were performed, always in the order temperature, magnesium concentration, buffer concentration with pH, other components. If multiple conditions were used, these are vertically aligned with the corresponding cleavage rates (described below) or separated by the word “or”. “Name HHR” lists the names of tested hammerhead ribozyme representatives, often by the species they originated from. “Type”: refers to the investigated hammerhead circular ribozyme permutation, type I, II or III (see main text). “Cleavage rate” refers to the ribozyme cleavage rate (k_{obs}) determined for the hammerhead representatives in cleavages per minute. The cleavage rate is dependent on the used assay conditions. For hammerhead ribozymes, it can be assumed that the cleavage rate increases with increasing reaction temperature, magnesium concentration and/or pH. The cleavage rates listed are collected under diverse assay conditions and it can be difficult to confidently estimate the cleavage rate with other conditions, e.g. of 25°C 10mM $MgCl_2$ and a pH of 7.5. “Comparison”: the word “slower” indicates previously studied hammerhead ribozymes that were timed under comparable or more favorable conditions compared to our experiments, and are slower than at least one of our non-circularly permuted ribozymes. “maybe same speed” indicates previously studied hammerhead ribozymes where the distinct conditions do not permit a clear comparison, but could plausibly be roughly as fast as the non-circularly permuted ribozymes. Our test conditions were 37°C, 25 mM $MgCl_2$, 80 mM HEPES, pH 7.5. The rows are sorted such that the “slower” rows and “maybe same speed” rows are adjacent, and otherwise sorted in increasing order of the highest speed per each paper. Note that interpretation of the speeds also requires considerations of the experimental conditions.

Reference	Format	Conditions	Name HHR	Type	Cleavage rate [min^{-1}]	Comparison
(7)	cis (protein-free)	42°C, 30 mM $MgCl_2$, MES, pH 6.9, 10 mM NaCl, 0.3 mM EDTA	*N. viridescens*<br>*Amb. talpoideum*<br>*Amp. tridactylum*	I	0.007<br>0.01<br>0.007	slower<br>slower<br>slower

(8)	cis (protein-free)	55°C, 10 mM $MgCl_2$, 50 mM Tris-HCl, pH 8.0, 0.5 mM EDTA	newt<br>newt-like<br>env1c	I	0.33<br>0.012<br>0.0024	 slower<br>slower
(9)	cis (protein-free)	30°C, 10 mM $MgCl_2$, 40 mM Tris, pH 8	*S. mansoni*	I	0.15	maybe same speed
(10)	cis (protein-free)	37°C, [1 mM or 10 mM $MgCl_2$], 10 mM Tris-HCl, pH 7.4, 140 mM KCl, 10 mM NaCl	see table 1 (different environmental seqs. such as bat guano, raw sewage)	I<br>II<br>III	0.15-1.7 or 7-28<br>0.04-15 or 11-37<br>0.008-0.74 or 0.15-21	 <br>maybe same speed
(11)	trans	25°C, 0.1mM $MgCl_2$, 50 mM HEPES, pH 7.0, 500 mM NaCl	*Schistosoma mansoni*	I/III	0.05	
(12)	cis (protein-free)	37°C, 2 mM $MgCl_2$, 10 mM Tris-HCl, pH 7.5	Xetr8 of *Xenopus tropicalis*	III	0.28	
(13)	trans	25°C, 0.1 mM $MgCl_2$, 50 mM Tris-HCl, pH 7, 100mM NaCl	*Schistosoma mansoni*	I/III	0.3	
(14)	cis (co-tr)	37°C, 0.1 mM $MgCl_2$, 50 mM Tris-HCl, pH 7.0	Smalpha1<br>PLMVd		0.93<br>0.36	
(4)	cis (protein-free)	37°C, 0.1 mM $MgCl_2$, 50 mM Tris-HCl, pH 7	sTRSV<br>vLTSV<br>PLMVd<br>HH2	III	1.2<br>1.4<br>0.71<br>0.02	
(15)	cis (protein-free)	25°C, [1 mM or 10 $MgCl_2$], 50 mM Mes, pH 6.0	HH16-T2 (derived from sTRSV natural sequence combined with minimal HHR HH16 (16))	III	0.54 or 1.67	
(17)	cis (protein-free)	25°C, [10 or 1 mM $MgCl_2$], 50 mM Tris-HCl, pH 7.5	*Serpula lacrymans*	I	1.97 or 0.16	
(3)	cis (co-tr)	37°C, 6mM $MgCl_2$, 40 mM Tris-HCl, pH 7	CChMVd-A10	III	1.95<br><br>1.58	

	cis (protein-free)<br>cis (protein-free)	25°C, 0.5 mM $MgCl_2$, 40 mM Tris-HCl, pH 7<br>25°C, 10 mM $MgCl_2$, 50mM PIPES-NaOH, pH 6.5,			2.43	
(18)	cis (protein-free)	25°C, 1 mM $MgCl_2$, 50mM MES, pH 6.0	rCSCVd+(nHH1-chimera);<br>PLMVd- (nHH2-chimera);<br>satArMV+ (nHH2-chimera);<br>satLTSV- (nHH4-chimera);<br>CChMVd+ (nHH5-chimera);<br>satSCMoV+ (nHH6-chimera);<br>satCYMoV+ (nHH7-chimera);<br>satLTSV+ (nHH8-chimera);<br>satTRsV (nHH9-chimera)	III	0.08 to >2.5	
(19)	cis (co-tr)	37°C, 6mM $MgCl_2$, 40 mM Tris-HCl, pH7	PLMVd +	III	3	
(20)	cis (protein-free)	25°C, 0.2 mM $MgCl_2$, 10 mM Tris-HCl, pH 7.5, 25mM NaCl, 0.1 mM EDTA,	*A. thaliana* Ara1<br>PLMVd	III	2.5<br>5.2	
(21)	cis (protein-free)<br><br><br>cis (co-tr)	25°C, 0.05 mM $MgCl_2$, 50 mM Tris-HCl, pH7.5<br><br>37°C, 6 mM $MgCl_2$, 40 mM Tris-HCl, pH 8, 10 mM NaCl	ELVd +<br>PLMVd +<br>CChMVd +<br>sTRSV +<br>ELVd +	III<br><br><br><br>III	0.3<br>0.57<br>0.96<br>0.08<br>7.95	
(22)	trans	25°C,<br>[0.5 mM $MgCl_2$,<br>1 mM $MgCl_2$,<br>5 mM $MgCl_2$]<br>50 mM Tris-HCl, pH 7.0, 100 mM NaCl, 0.1 mM EDTA	*S. mansoni* 26<br>*Dolichopodo baccettii* &<br>*Dolichopoda schiavazzii*	III	*Dolichopoda* vs. *S. ma.* 26<br>0.15 versus 3<br>0.4 versus 5.9<br>3 versus 30	

Supplementary Figures

Supplementary Figure 1. HRIMA-2 to HRIMA-6 motif consensus features. The annotations are the same as in Figure 1C, except that the 100% conservation level is not depicted. The HRIMA-1 motif corresponds to type-SGC hammerhead ribozymes, and is shown in Figure 1C.

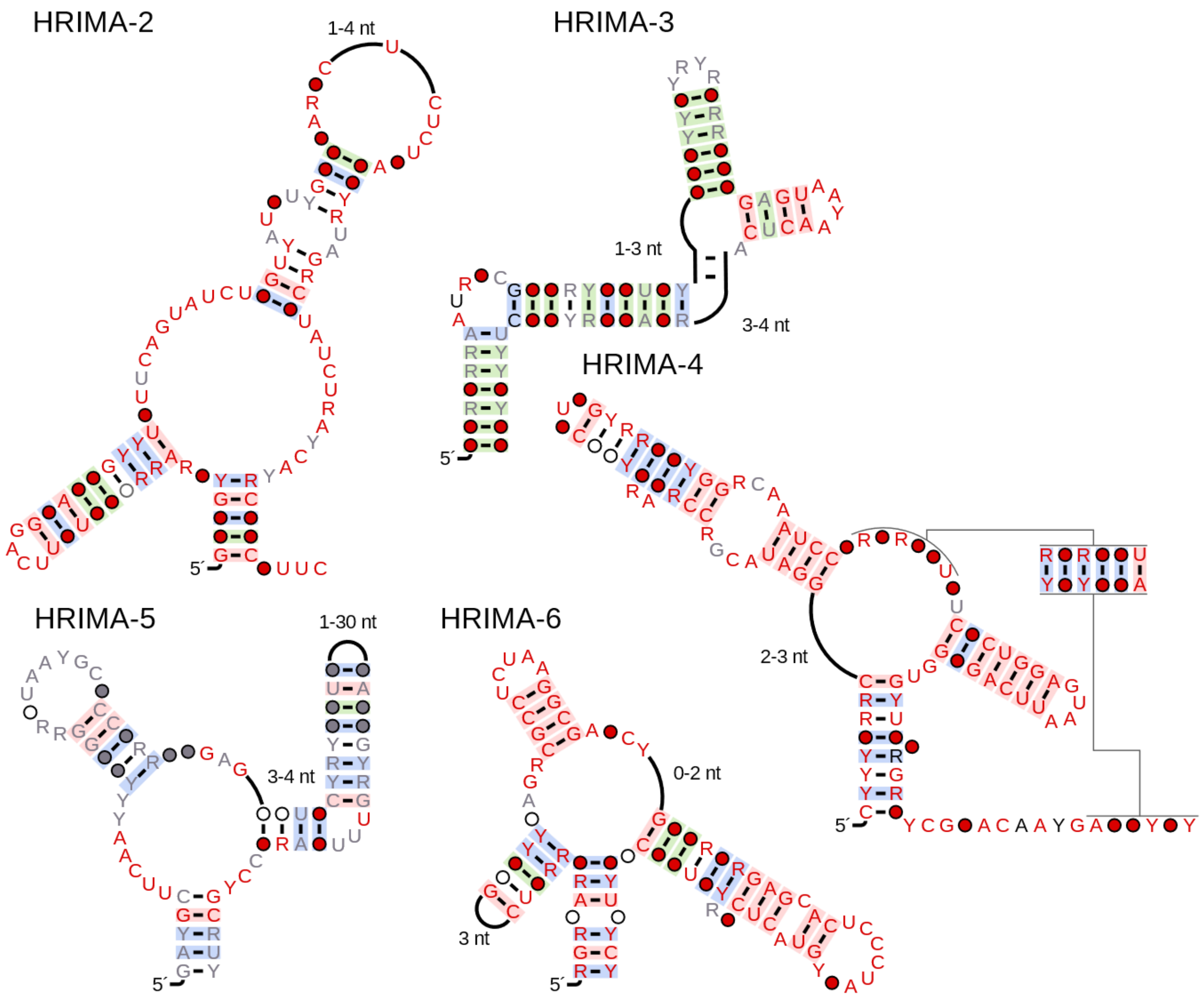

Supplementary Figure 2. HRIMA-7 to HRIMA-10 motif consensus features. The annotations are the same as in Figure 1C, except that the 100% conservation level is not depicted.

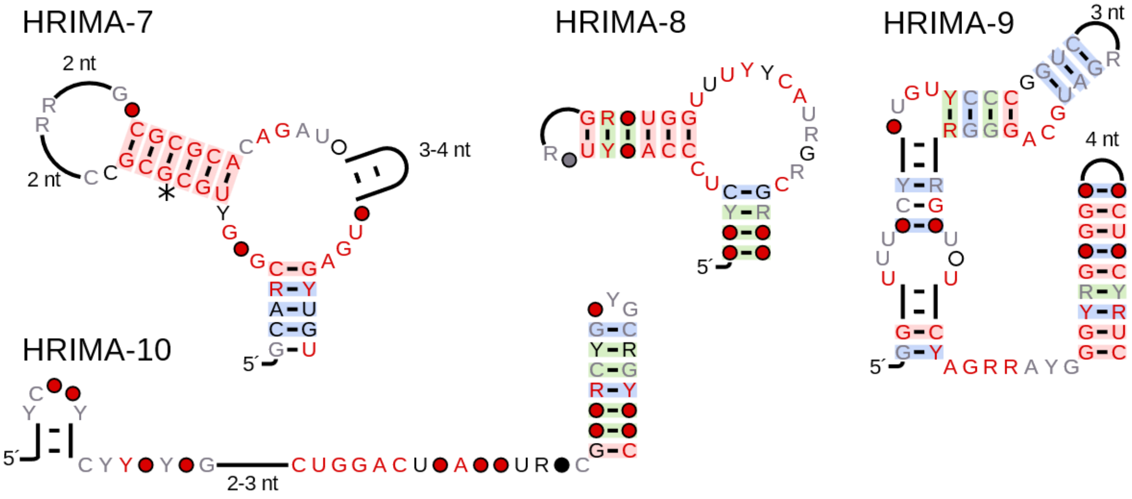

Supplementary Figure 3. HRIMA-11 to HRIMA-14 motif consensus features. The annotations are the same as in Figure 1C, except that the 100% conservation level is not depicted.

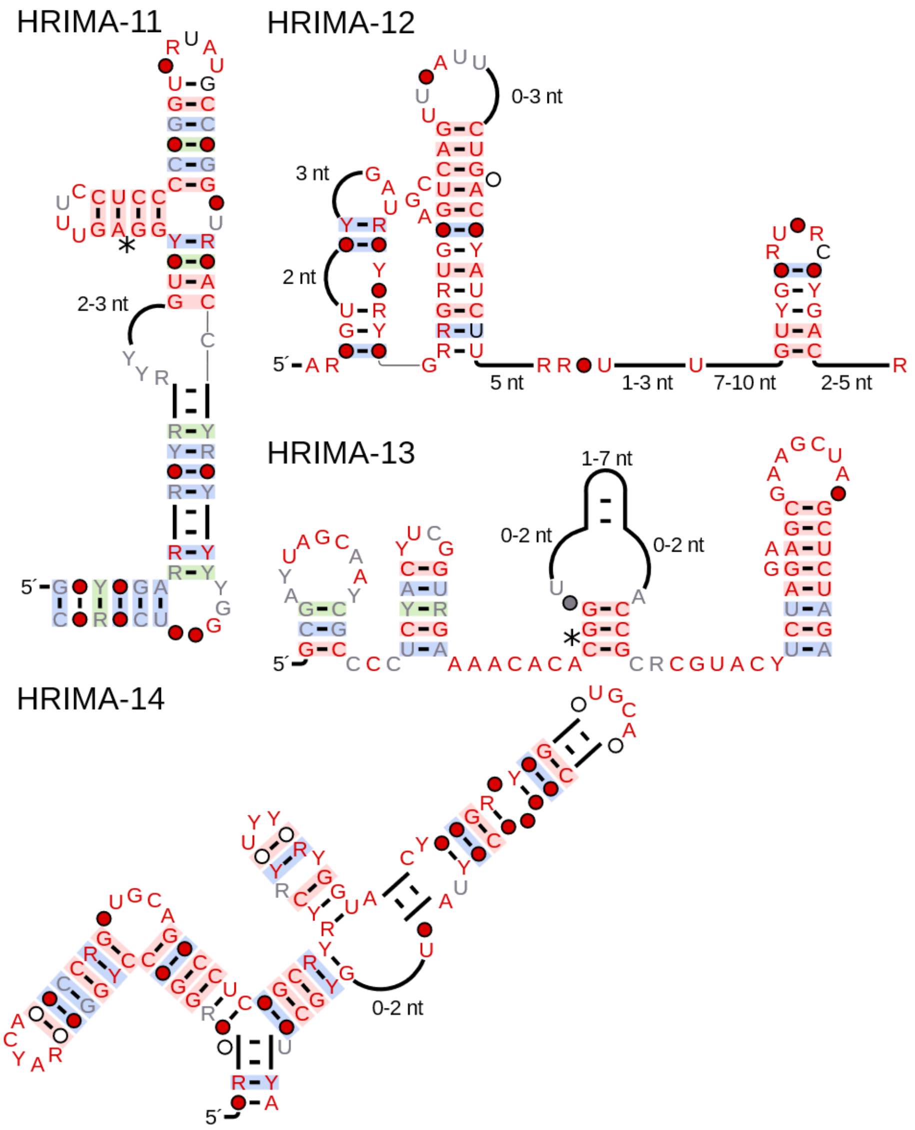

Supplementary Figure 4. HRIMA-15 to HRIMA-20 motif consensus features. The annotations are the same as in Figure 1C, except that the 100% conservation level is not depicted. Asterisks mark helices whose existence is especially uncertain, based on our subjective evaluation of covariation. The HRIMA-19 motif has high levels of covariation, but little sequence conservation. This lack of sequence conservation could suggest that the aligned sequences are not related to each other, and therefore that the covariation is misleading. However, we notice that there is covariation even within groups of sequences that are highly similar to one another in sequence, which suggests that the covariation is biologically significant.

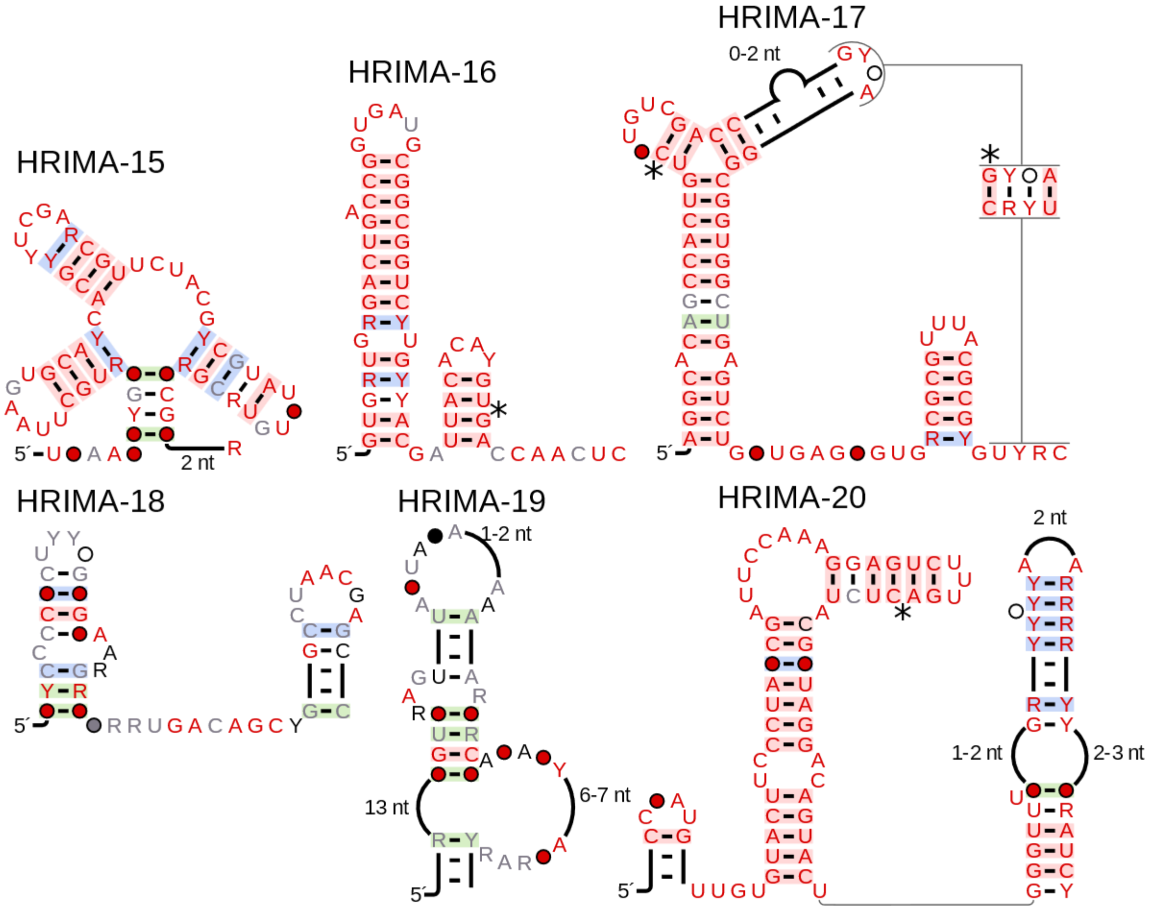

Supplementary Figure 5. HRIMA-21 to HRIMA-26 motif consensus features. The annotations are the same as in Figure 1C, except that the 100% conservation level is not depicted. Asterisks mark helices whose existence is especially uncertain, based on our subjective evaluation of covariation.

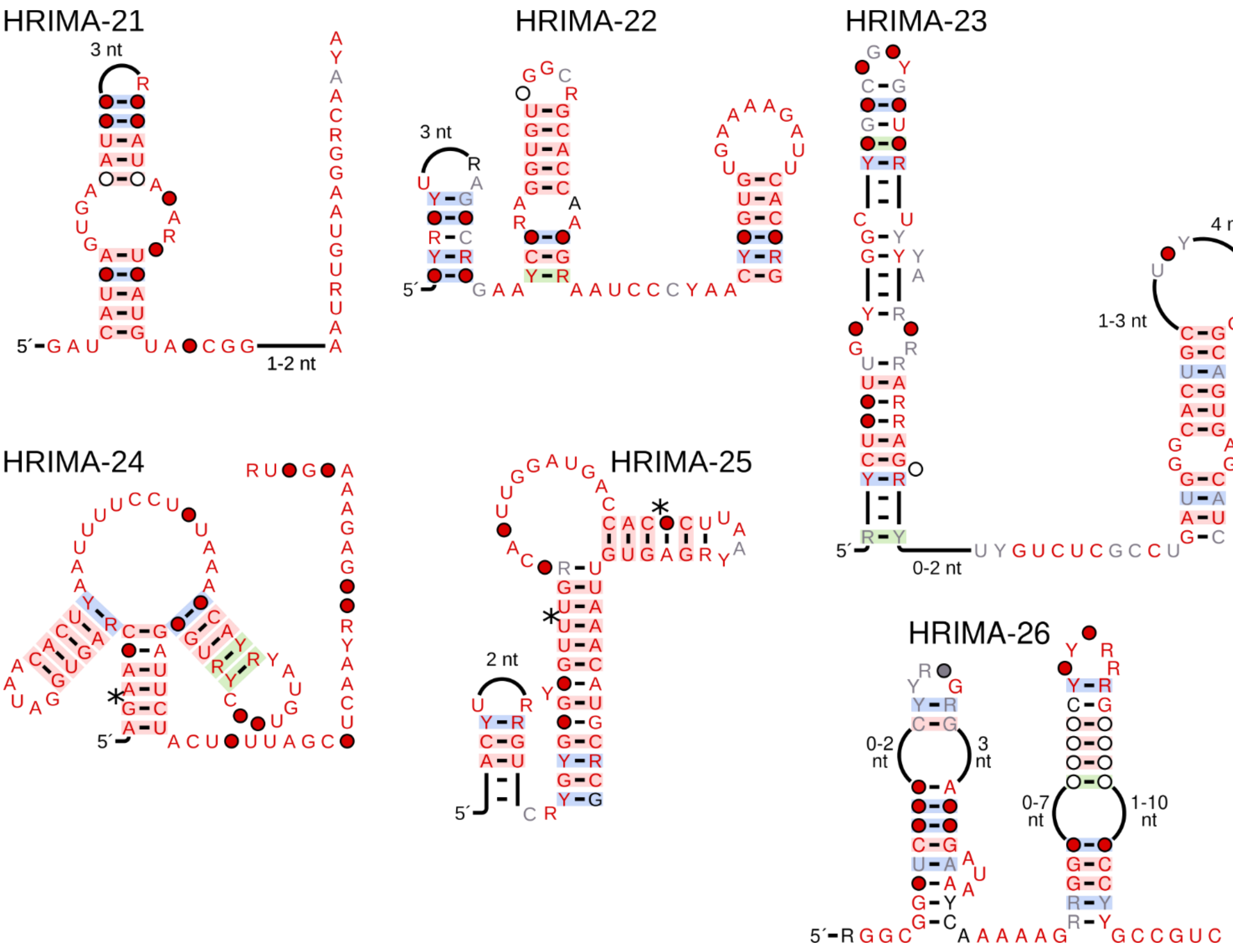

Supplementary Figure 6. Multiple-sequence alignment of type-SGC hammerhead ribozymes corresponding to the HRIMA-1 motif, unique sequences only. Shading indicates stems: stem I, stem II, stem III, helix joining stems I and II, optional hairpin. Non-canonical base pairs in these positions are shaded in gray. Base pairs are also indicated in the 2nd-last through 4th-last lines. Pairs are indicated by less than and greater than symbols (< and >), which are used like nested brackets. The last line is the consensus sequence. Nucleotides that are at least 97% conserved are labeled in this line in red. The caret (^) in this line indicates the cleavage site, for orientation. The conserved sequences CUGA.GA and GAAA and the cleavage site in the hammerhead ribozyme core are underlined, also for orientation. Numbers on the right side mark sequences that were experimentally tested (1=SGC-1, 2=SGC-2, etc.). Also tested were two sequences marked with an asterisk (*), which have deviations in otherwise conserved nucleotides (Supplementary Figure 9). Note: the 5′ and 3′ ends of tested sequences differ from those shown in the alignment.

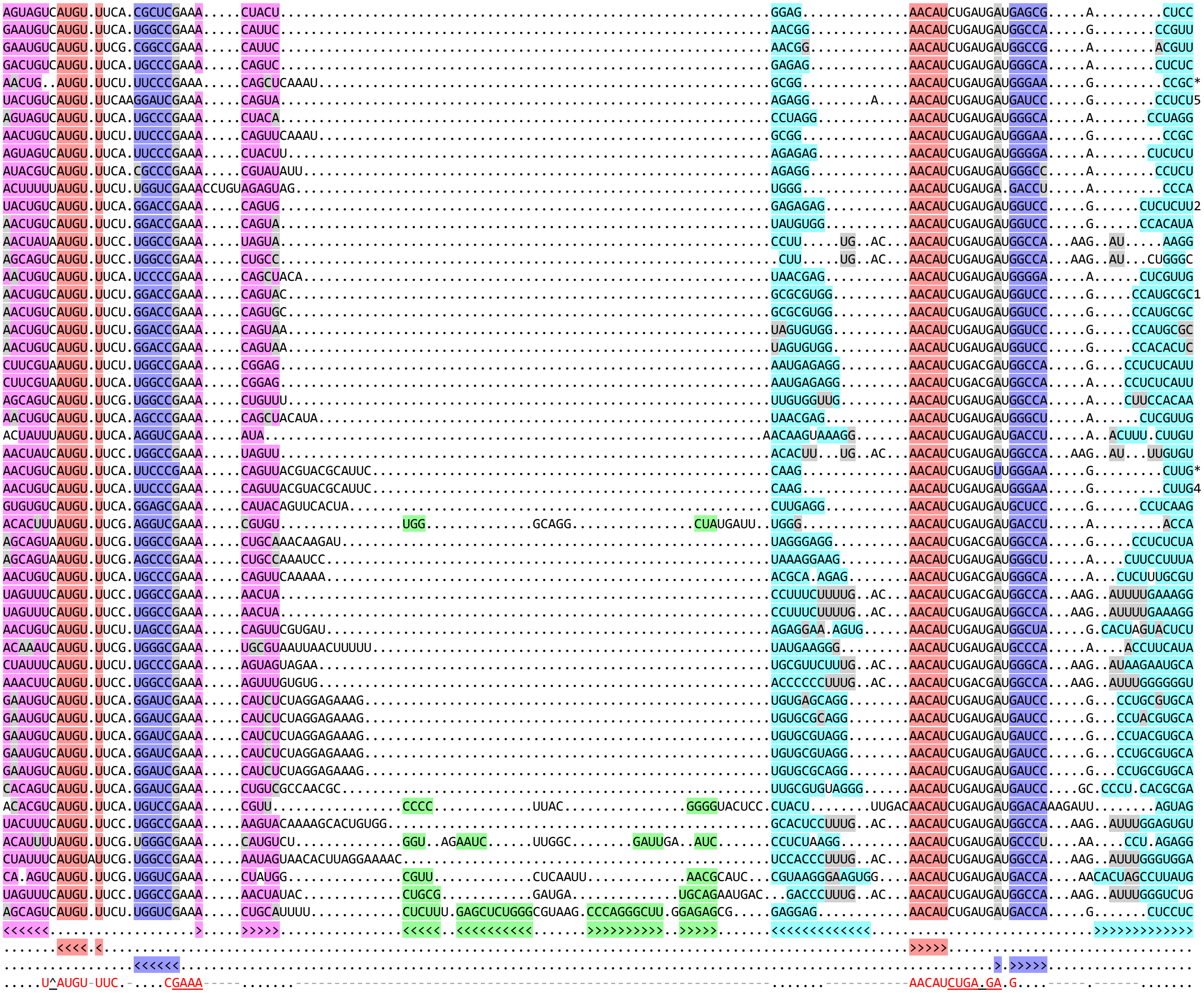

Supplementary Figure 7. Consensus diagram of the type-SGC hammerhead ribozyme variants. Annotations are the same as in Figure 1C.

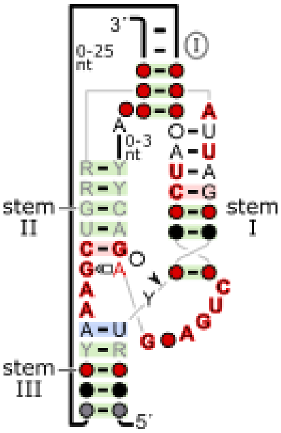

Supplementary Figure 8. Multiple-sequence alignment of type-SGC hammerhead ribozyme variants, unique sequences only. Annotations are the same as in Supplementary Figure 6. As in that figure, shading indicates stems: stem I, stem II, stem III, helix joining stems I and II, optional hairpin. In the consensus sequence, the last nucleotide of the conserved GAAA sequence is black, because one sequence (with asterisk on the right side) has an A-U base pair instead of the normal U-A. This sequence was tested and is marked with an asterisk on the right side. The SGC-3 sequence is marked with a ‘3’. Note: the 5′ and 3′ ends of tested sequences differ from those shown in the alignment.

```
..AUUAUAGUCUAGAAUUAUGGACGAAACUAUAAU...........................CUAUAUUCUAUCCUC.........CUAUUUAUAUCAUCCUACUGAUGA.GUCCA..AAUAUAUAAAUAG.........
..CACUAUGUUUUAGAUUAUGGUCGAAACAUAUUG...........................AUC....................UCGUAGAUCCC.AUCUAACUGAUGA.GACCU..AAUACGAUCUACGA........
....CUAUGUUUUAGAUUAAGGUCGAAACAUA..............................ACUUCC...........GAAUC..GUGACUUCAUUAUCUAACUGAUGAUGACCU..AAAAUGAAGUCUAGAGAUUC..
.....UAUGUUUUAGAUUAAGGUCGAAACAUA..............................ACCUCC............GAAUCGUGACUUCAUU.AUCUAACUGAUGAUGACCU..AAAAAUGAAGACACUAUUC...
...AAACGAACUACGAUUAGAGUCGAAUUCGUUU............................U............................CCCAGGAUCGUACUGAUGAUGACUC..AACCUGGG..............*
.....AGUAUCUUGGAUAAAGGACGAAAUACU...........................................................UGCUC.AUCCAACUGAUGA.GUCCUAAAGCGAGUA..............
.....AGAGUUUUAGAUUAGGGUCGAAACUC...............................AUAUCC.........GACAUUUU.AUAAAUGCUC.AUCUAACUGAUGAUGACCU...AAGAACAUUUAUCAAGAUGUU
.....UACGUCUAGGUUUAUGGUCGAAACGUG..............................AAACCUUAAA..................ACUUGGGGUCCUACUGAGGAUGACCA..AACCCAAGU.............
.....CACGUCUAGGUUUAUGGUCGAAACGUG..............................AAACCUAAAA..................ACUUGGGGUCCUACUGAGGAUGACCA..AACCCAAGU.............
.....CACGUCUAGGUUUAUGGUCGAAACGUG..............................AAACCUUAAA..................ACUUGGGGUCCUACUGAGGAUGACCA..AACCCAAGU.............
....ACCAUUCUGAGAUUAGAGUCGAAAAUGGU.....................................................ACA.AGUUAAGAUCUCACUGAUGA.GACUC.UAACUUGACUGUGU.........
...AA.GCGUCU.AGAUUAGAGUCGAAACGCAUU.......................................................UAGGUACGAUCU.ACUGAUGAUGACUC...ACGUACCUG............
.AUCAAAACUUAAUGAUUAGGGUCGAAAGUUUUGAU.............................................UAAUUCAAUUUGCGU.AUCAUUCUGAUGA.GACCU...AAACGUA..UUGAAUUA....
.......CAUCAUGGAUUAGGAUCGAAAUG................................AU.......................AGGUUUGUGGAUCCAUCUGAUGAUGAUCC..AACCACGAAUCU..........
.UUUCAAAAUCUAAGAUUAGGAUCGAAAUUUUGAAA.....................................................AUAGGAAGAUCUUACUGAAGAUGAUCC..AACUUCCUAU............
....UCCUUUCUGUGGUUAGAGUCGAAAAAGGA.............................UUA........................UAAGAUCAAUCACACUGAUGAUGACUC..AAUGAUCUUA............
.....GCUAUCUGUGAUCAGAGUCGAAAUAGC..............................C............................AGUCA.AUCACACUGAUGAUGACUCACAACUGGCU..............
....UCCAAUCAGAGAUUAGAAACGAAAUUGGA..................................................UGUCAAC..CCGGUAUCUCUCUGAAGAUGUUUC..AAACCGGCGGUUGACA......
.....CCAAUCAGAGAUUAGAAACGAAAUUGG..............................AAU...................GUUAAC.CCGGU.AUCUCUCUGAAGAUGUUUC...GAGCCGCGGUUAAC.......
.....AAUAUAAAUGAUUAUGGUCGAAAUAUU..............................CAACAACU..........UGCUAACCACUUUUCUUAUCAUUCUGAUGA.GACCA..AAAAGAAAU.UGG..AGCA...
.....AAUAUAAAUGAUUAUGGUCGAAAUAUU..............................CAACAACU..........UGCUAACCGCUUUUCUUGUCAUUCUGAUGA.GACCA..AAAAGGAAA.CGG..AGCA...
.....AAUAUAAAUGAUUAUGGUCGAAAUAUU..............................CAACAUCU..........UGCUAACCGCUUUUCUUGUCAUUCUGAUGA.GACCA..AAAAGGAAA.CGG..AGCA...
.....AAUAUAAAUGAUUAUGGUCGAAAUAUU..............................CACGCUUCU...........UGCUAACCACUUUUUAUCAUUCUGAUGA.GACCA..AAGAAAAUUGGU..GCA.....
.....AAUAUAAAUGAUUAUGGUCGAAAUAUU..............................CACGCUUCUCGC............UACCACUUUUUAUCAUUCUGAUGA.GACCA..AAGAAACUUGGUG.........
.....AACUUCUGUGAUUAUCGUCGAAAAGUU........................................................UCACUUGU.AUCACACUGAUGAUGACGA...AAACAAGUGA...........
.....GAACUCUGUGAUUAACGUCGAAAGUU............................................................CAACC.AUCACACUGAUGAUGACGU...AAGGCUG..............
GUAACAAAAUCAGUGAUUAGGGUCGAAAUUUUGUUGC.........................GCA.....................GUAG.AAUCGGAUCACUCUGAUGUUGACCC..AACCGGUUUCUGC.........
GUAACAAAAUCAGUGAUUAGGGUCGAAAUUUUGUUGC.........................GCA.....................GUAG.AAUCGGAUCACUCUGAUGAUGACCC..AACCGGUUUCUGC.........
..ACCAGUGUCACGGAUUAGGACCGAAACAUUGGU..GCCACGGCAUUGUCGUUGCCGUGGC........................AGUGCCUUGGGAUCCGUCUGAGGAUGGCCC..AAACCUUGGCAUU.........
.....GCAAUCUGUGAUUAGUGUCGAAAUUGC..........................................................UUACGUGAUCAUACUGAUGAUGACAC..AACACGUAA.............3
.....GCCAUCAGAGAUUAGGAUCGAAAUGGU........................................................GCUAUUGUGAUCUUUCUGAAGAUGAUCC..AACACAUUAGU...........
.....GCCAUCAGAGAUUAGGGUCGAAAUGGC.......................................................GGUUUGGGUGAUCUUUCUGAAGAUGACCU..AACACCCAUUCC..........
....UCUCGUCAGCGAUUAGGGUCGAAACG...............................................................AGG.AUCACUCUGAAGA.GACCC...AACCU................
...................<<<<<<....................................................................................>.>>>>>........................
<<<<<<<<<<.................>>>>>>>>>><<<<<<<<<.......>>>>>>>>>...............<<<<<<<<<<<<<<<<<<<.........................>>>>>>>>>>>>>>>>>>>
...........<<<<<<<..............................................................................>>>>>>>.....................................
-----....U^.....U.A....CGAAA....------------------------------.------------------------...........UC...CUGA.GA.G....---...........----------
```

Supplementary Figure 9. Structure and *in vitro* experiments for predicted type-SGC hammerhead ribozymes with aberrant nucleotides. (The graphic is on the next page.) (**A**) Projected secondary structure of a predicted type-SGC hammerhead ribozyme with an A to U mutation, indicated by the red circle. The M1 mutation is predicted, based on previous hammerhead ribozyme research, to essentially inactivate the ribozyme. A lower-case 'g' represents a non-biological G nucleotide added to facilitate transcription by T7 polymerase. (**B**) Secondary structure of predicted type-SGC hammerhead ribozyme with an A-U to U-A base-pair mutation (red outline). (**C**) Secondary structure of a type-SGC predicted hammerhead ribozyme missing two nucleotides that would normally be found immediately 3′ to the cleavage site. The red outline indicates where the missing nucleotides would be located by analogy with the other hammerhead ribozyme drawings. (**D**) PAGE image showing results of *in vitro* transcription of RNA molecules with [α-^{32}P] ATP for 2 h. The M1 mutation of the predicted ribozyme with an A to U mutation in part A was used to create ladders by partial digestion with ("T1") or alkaline hydrolysis ("$^{-}$OH"). The full-length molecules ("FL") are marked, based on their names in parts A-C. The wildtype and M1 molecules in part A have the same length of 81 nucleotides. The locations of anticipated 5′ and 3′ cleavage products ("5′-clv" or "3′-clv", respectively) are also marked. All tested molecules in this gel have predicted 5′ cleavage products of 14 nucleotides.

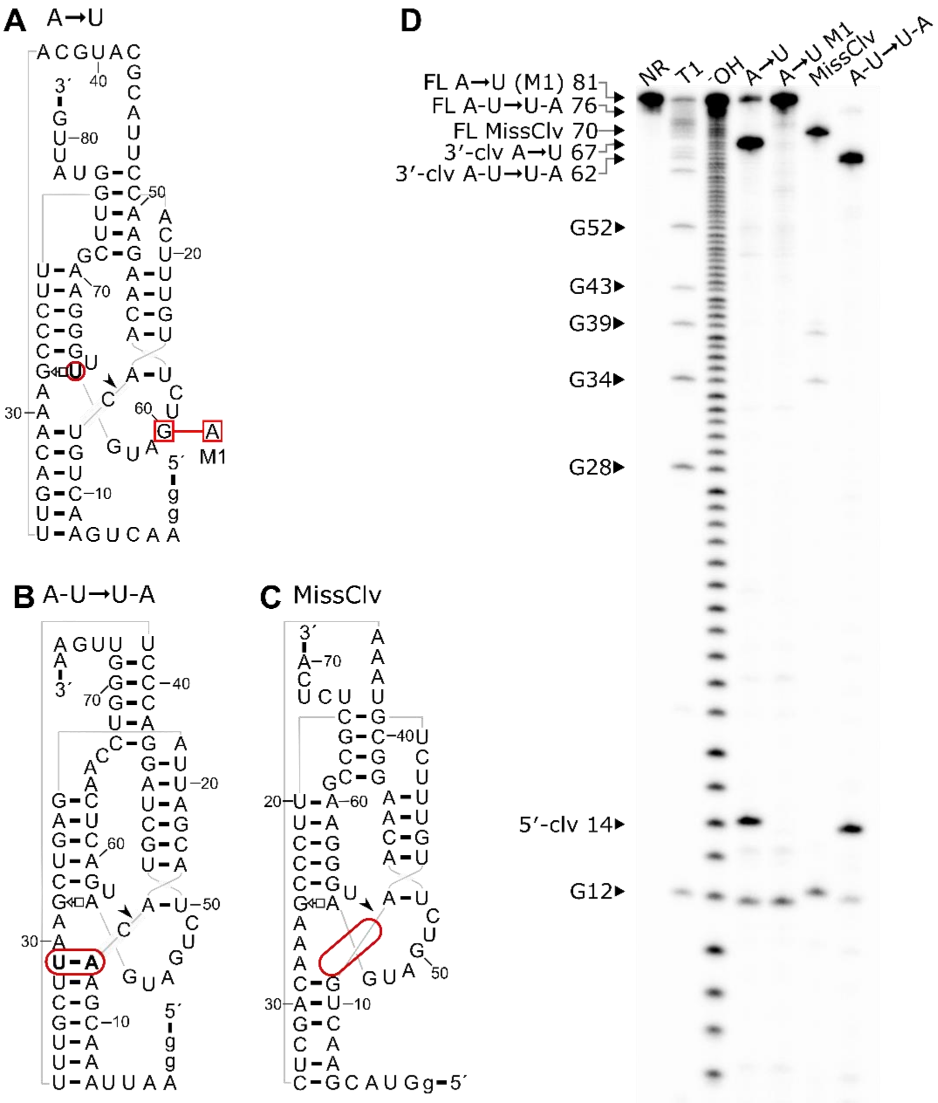
A A→U
M1
B A-U→U-A
C MissClv
D
NR
T1
-OH
A→U
A→U M1
MissClv
A-U→U-A
FL A→U (M1) 81
FL A-U→U-A 76
FL MissClv 70
3′-clv A→U 67
3′-clv A-U→U-A 62
G52
G43
G39
G34
G28
5′-clv 14
G12

Supplementary Figure 10. Multiple-sequence alignment of type-CSG hammerhead ribozymes, unique sequences only. Annotations are the same as in Supplementary Figure 6. As in that figure, shading indicates stems: stem I, stem II, stem III, helix joining stems I and II. Black dots in the last row indicate the eight positions where the nucleotides in the two sequences differ. Both sequences were tested and are marked on the right side (1=CSG-1, 2=CSG-2). Note: the 5′ and 3′ ends of tested sequences differ from those shown in the alignment.

```
AAUGCGGUUGGUUGCUGAUGAGGGGUGCCGCAUUAAGUUGUCCAACCAUUAAUACCCCGAAACAAC1
AAUGUGGUUGGUUGCUGAUGAGGGUGGCCACAUUCAGUUGUCCAACCAUUAAACACCCGAAACAAC2
........<<<<<<............................>>>>>>..................
....................<<<<<<...........................>>>>>>.......
<<<<<<<<..................>>>>>>>>..<<<<<....................>>>>>
AAUG.GGUUGGUUGCUGAUGAGGG..GCC.CAUU.AGUUGU^CAACCAUUAA...CCCGAAACAAC
```

Supplementary Figure 11. Identification and co-transcriptional cleavage of a type-GCS hammerhead ribozyme. (**A**) Secondary structure diagram of candidate GCS-1 depicting the ribozyme layout with its three stems. As we only found one sequence, there is no diagram showing conserved features. The M1 mutant and other annotations are as described in Figure 1 and 2. (**B**) PAGE image showing GCS-1 ribozyme after *in vitro* transcription in the presence of [α-^{32}P] ATP for 2 h. See description of Figure 2. Transcription bands for WT (wildtype) ribozyme RNA correspond to the 3′ cleavage products (20 nucleotides) and a 5′ cleavage product of 53 nucleotides. The full-length (FL) transcript of the M1 ribozyme mutant is visible at 73 nucleotides.

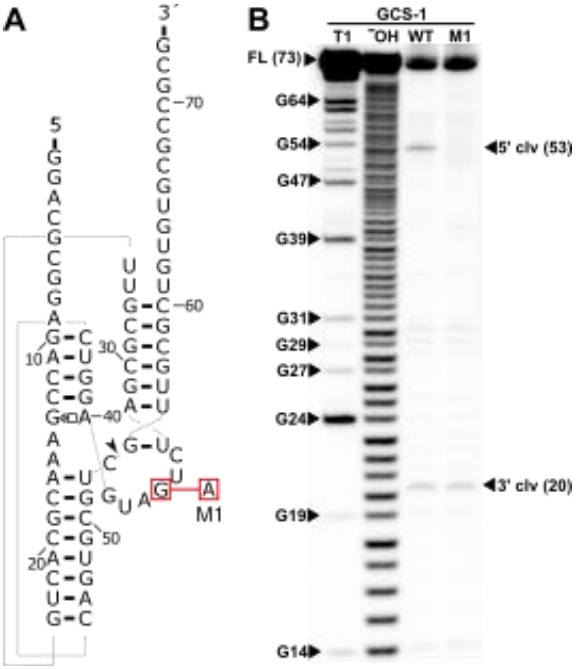

Supplementary Figure 12. Sequence of type-GCS hammerhead ribozyme. The tested sequence GCS-1 has additional nucleotides on the 5′ and 3′ ends. The helix annotated as belonging to stem III could equally be characterized as corresponding to the interaction that brings stem I and stem II in proximity. Annotations are the same as in Supplementary Figure 6. As in that figure, shading indicates stems: stem I, stem II, stem III.

```
GACCGAAACGCACUGUUGCGCGAUCUGAUGAGGUCCAGUGCGUCGUUGCGC
.......<<<<<<<<....................>>>>>>>>........
<<<<...........................>>>>................
.................<<<<<<<....................>>>>>>>
GACCGAAACGCACUGUUGCGCGAUCUGAUGAGGUCCAGUGCGU^GUUGCGC
```

Supplementary Figure 13. Theoretical non-circular permutations of twister ribozymes. (**A**) The previously established type-P1 twister ribozyme (23) is decomposed into blocks A, B and C, based on previously established multiple-sequence alignments. Type-P1 twister ribozymes correspond to type ABC in the proposed nomenclature. Conservation patterns use the same annotations as in Figure 1C, but not all conserved nucleotides or features are depicted here or in part B. ‘pk1’ and ‘pk2’ indicate the two pseudoknot interactions (23). The other previously established circular permutations of type P1, called type P3 (type BCA) and type P5 (type CAB) are not depicted. (**B**) Three non-circular permutations predicted by rearranging blocks A, B and C that were not previously proposed. Annotations are as in part A. Note: we did not find any biological sequences that we believe correspond to these proposed permutations. Therefore, the conservation patterns (i.e. coloring of nucleotides) are copied from part A and not based on any biological sequences.

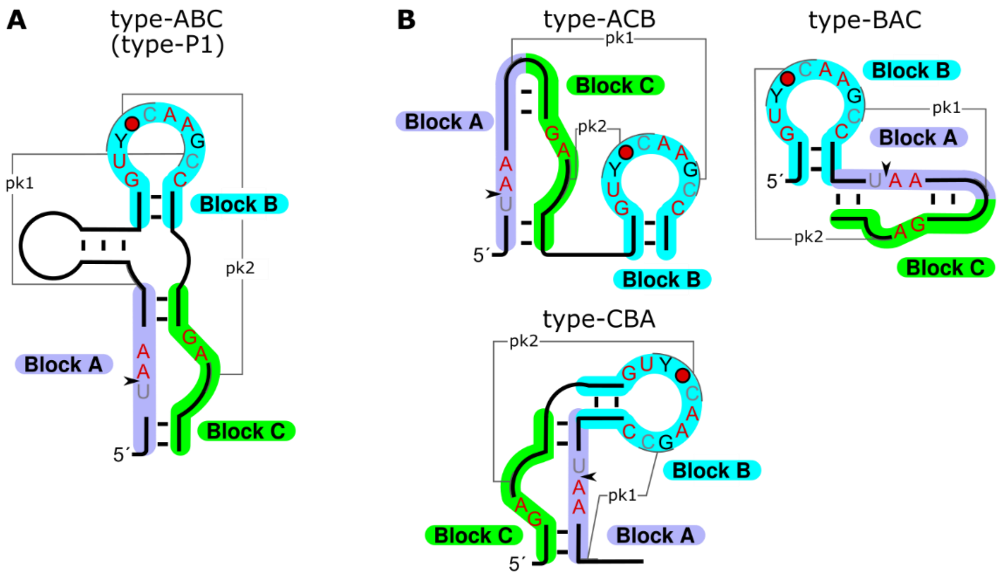

Supplementary Figure 14. Determination of observed rate constants for ribozyme cleavage *in cis*. (**A**) PAGE image showing SGC-1 ribozyme cleavage at different times of an *in vitro* transcription reaction in the presence of [α-^{32}P] ATP. Transcript bands for full-length ribozyme RNA are visible at 79 nucleotides and for the 3′ and 5′ cleavage product at 64 nucleotides and 15 nucleotides, respectively. Band intensity for the cleavage products increases over time. (**B**) FL and ribozyme cleavage product bands were quantified using ImageQuant software and used to calculate the fraction remaining by using the following calculation: fraction remaining = 1 – (sum of the intensities of 3′ + 5′ cleavage products)/(sum of intensities of FL + 3′ + 5′ cleavage products). (**C**) Corresponding plot of the fraction remaining versus time in minutes drawn using GraphPad Prism software. Line shows the fit according to a one-phase exponential decay. The observed rate constant k_{obs} is given in cleavages per minute (min^{-1}) and R^2, the coefficient of determination, which indicates the goodness-of-fit to the kinetic model, is shown.

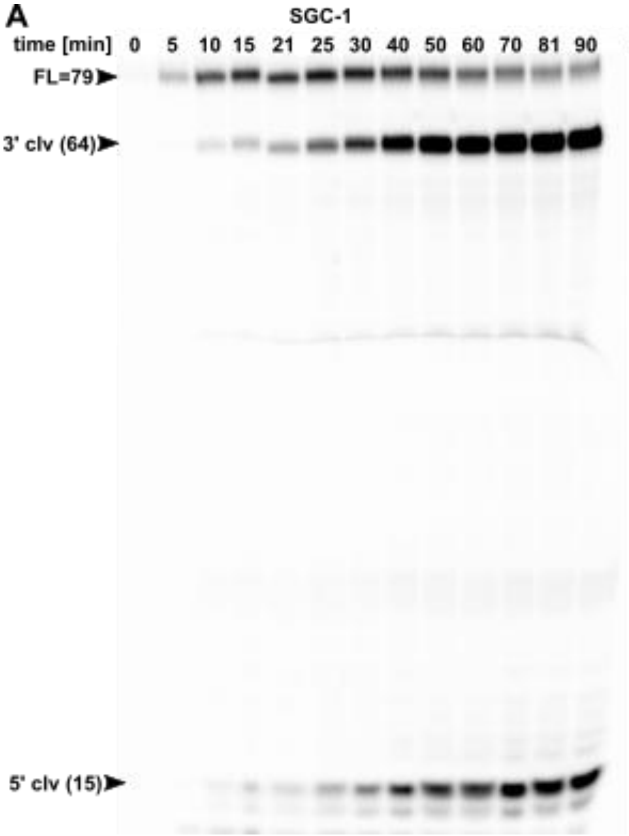

B

time [min]	band intensity FL	3' clv	5' clv	fraction remaining
0	47	-1	1	1.0
5	354	16	12	0.92
10	967	153	74	0.81
15	1189	304	152	0.72
21	1108	468	190	0.63
25	1372	924	403	0.51
30	1366	1298	591	0.42
40	1291	2147	937	0.30
50	1189	3025	1226	0.22
60	978	3377	1338	0.17
70	913	3930	1796	0.14
81	795	4200	1746	0.12
90	746	4297	1822	0.11

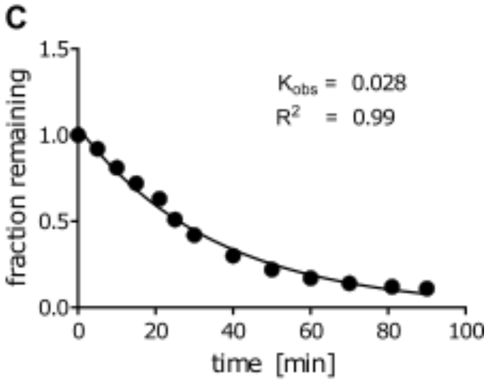

Supplementary Figure 15. Plots of the fraction remaining versus time to determine observed rate constants. Up to four time-course experiments are depicted per plot. To determine the observed rate constant (k_{obs}) data were fit to a one-phase exponential decay as described in methods and as illustrated in Supplementary Figure 14. To simplify the plot only one fit is shown as curve. K_{obs} and goodness of fit are given below each plot for hammerhead ribozyme types SGC-1 (**A**), shortSGC-1 (**B**), SGC-2 (**C**), shortSGC-2 (**D**), SGC-3 (**E**), shortSGC-3 (**F**). Where applicable, mean values and standard deviations were calculated and are shown in Table 1. Experiment 2 (part A) was also shown in Supplementary Figure 14C.

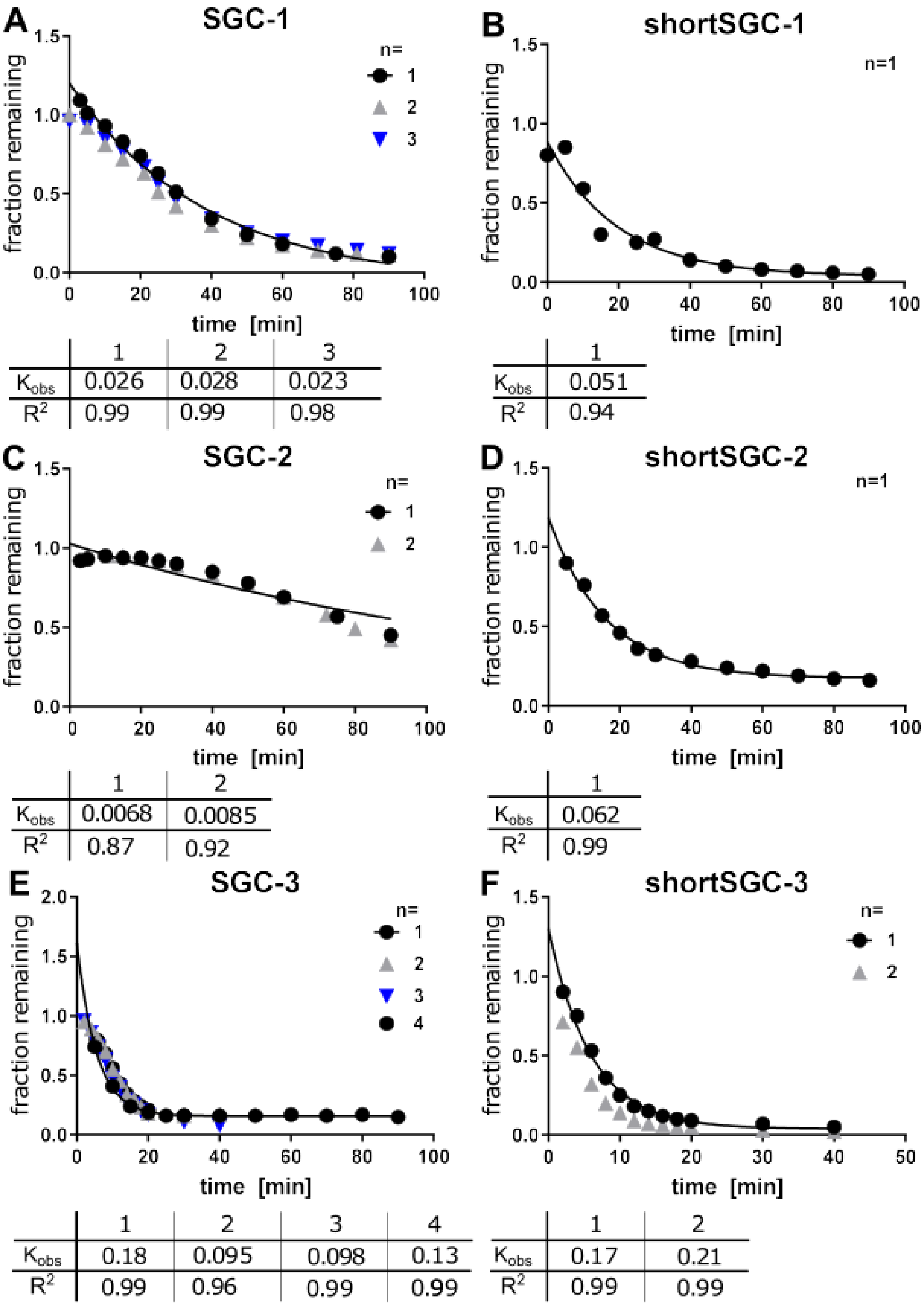

A

	1	2	3
K_{obs}	0.026	0.028	0.023
R^2	0.99	0.99	0.98

B

	1
K_{obs}	0.051
R^2	0.94

C

	1	2
K_{obs}	0.0068	0.0085
R^2	0.87	0.92

D

	1
K_{obs}	0.062
R^2	0.99

E

	1	2	3	4
K_{obs}	0.18	0.095	0.098	0.13
R^2	0.99	0.96	0.99	0.99

F

	1	2
K_{obs}	0.17	0.21
R^2	0.99	0.99

Supplementary Figure 16. Plots of the fraction remaining versus time to determine observed rate constants. Up to three time-course experiments are depicted per plot. To determine the observed rate constant (k_{obs}) data were fit to a one-phase exponential decay as described in methods and as illustrated in Supplementary Figure 14. To simplify the plot only one fit is shown as curve. K_{obs} and goodness of fit are given below each plot for hammerhead ribozyme types SGC-4 (**A**), SGC-5 (**B**), CSG-1 (**C**), CSG-2 (**D**), GCS-1 (**E**). Where applicable, mean values and standard deviations were calculated and are shown in Table 1.

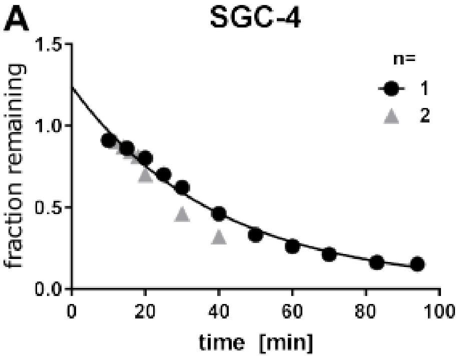

	1	2
K_{obs}	0.025	0.038
R^2	0.99	0.98

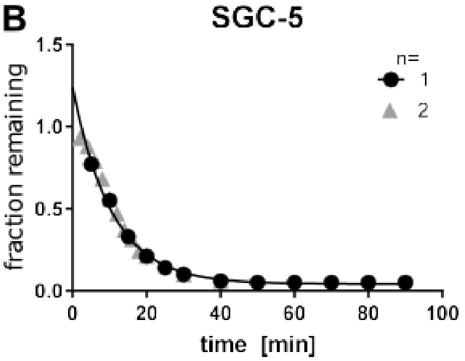

	1	2
K_{obs}	0.095	0.073
R^2	0.99	0.98

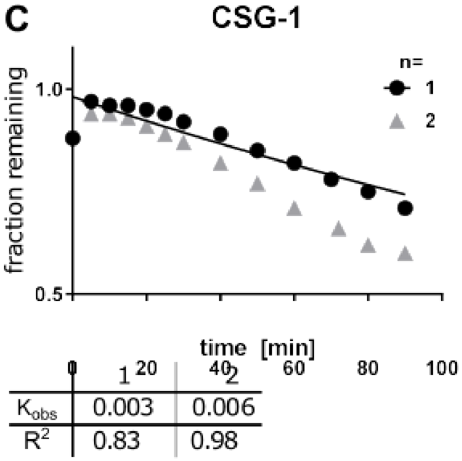

	1	2
K_{obs}	0.003	0.006
R^2	0.83	0.98

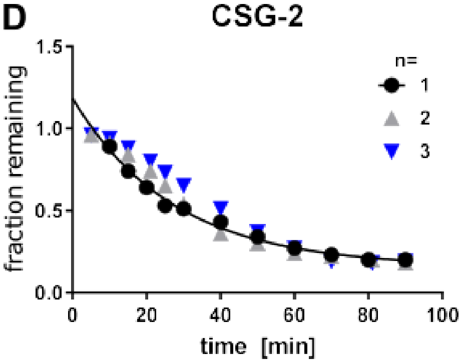

	1	2	3
K_{obs}	0.037	0.027	0.015
R^2	0.99	0.98	0.98

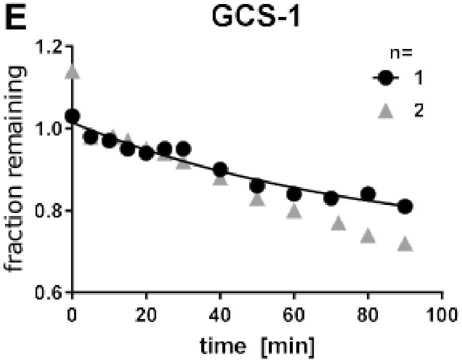

	1	2
K_{obs}	0.012	0.015
R^2	0.95	0.94

Supplementary Figure 17. Stem III in type-GCS hammerhead ribozymes could also function as a stem I/stem II interaction. (**A**) Drawing of the putative type-GCS hammerhead ribozyme, emphasizing the location of stem III, which encompasses nucleotides 20 and 50, among others. (**B**) A drawing of the exact sequence and secondary structure depicted in part A, but with a different layout on the page. In this case, stem III is in the position of the interaction that brings stems I and II together. As before, it includes nucleotides 20 and 50. This is enabled by the connectivity of the type-GCS permutation. Note that in order to simultaneously function as stem III and the stem I/stem II interaction, the helix might require some flexibility in its geometry, and its length might be highly constrained so that both of its ends are positioned correctly. It is not clear if these constraints can be satisfied in 3D.

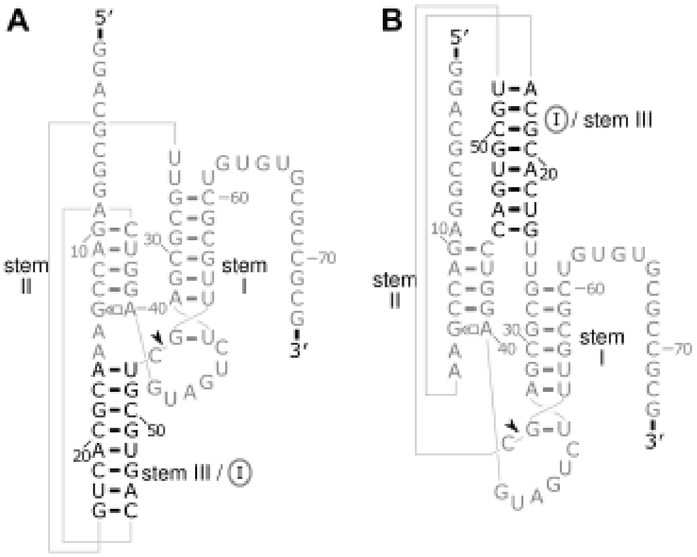

Supplementary Figure 18. Comparison of type-SGC hammerhead ribozymes and a previously crystallized hammerhead ribozyme (24) (PDB accession: 3ZP8). (The crystallized ribozyme was built out of two RNA molecules, so two 5′ and two 3′ ends are shown.) The consensus of type-SGC ribozymes corresponding to the HRIMA-1 motif is shown (similar to Figure 1C). The core regions in both structures are shaded in pale yellow. The core region of the type-SGC ribozymes contains all key nucleotides present in the crystallized ribozyme, and presumably adopts a similar 3D structure. Circled symbols indicate a helical interaction (circled ‘I’) and two linker regions (circled ‘+’ and ‘?’) in type-SGC hammerhead ribozymes. The linkers are sometimes short and they are unique to the non-circular permutations of hammerhead ribozymes. The table at the bottom of the figure shows correspondences between type-SGC ribozymes and the crystallized molecule. Both types of hammerhead ribozymes have interactions between stems I and II (circled ‘I’), though the crystallized molecule uses non-Watson-Crick interactions, where type-SGC ribozymes predominantly or exclusively use a helix. The linker between stems I and II in type-SGC ribozymes is analogous to a single base pair in the crystallized ribozyme (circled ‘+’), and both would cause the relevant sides of stems I and II to be in close proximity. A further linker in type-SGC ribozymes (circled ‘?’) is sometimes as short as zero nucleotides, and the adjacent nucleotides are not located near to each other in the crystal structure. These nucleotides, however, must be nearby to each other in the type-SGC ribozyme as they are covalently bonded. A similar short linker connecting the ends of the same stems is also present in type-CSG hammerhead ribozymes. The text suggests how these short linkers might be accommodated in the hammerhead ribozyme structure.

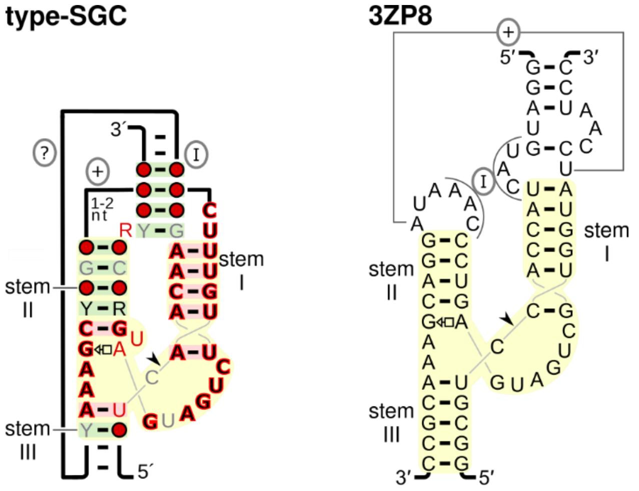

Comparison of interactions/linkers

Symbol	type-SGC	3ZP8
(I)	Helix	Non-WC pairs
(+)	Linker (3-4 nt)	Non-WC pair
(?)	Linker (0-44 nt)	?
	Core regions are compatible	

REFERENCES

1. Weinberg, C.E., Olzog, V.J., Eckert, I. and Weinberg, Z. (2021) Identification of over 200-fold more hairpin ribozymes than previously known in diverse circular RNAs, *Nucleic Acids Res.,* **49,** 6375-6388.
2. Reiche, K. and Stadler, P.F. (2007) RNAstrand: reading direction of structured RNAs in multiple sequence alignments, *Algorithms Mol. Biol.,* **2,** 6.
3. de la Peña, M., Gago, S. and Flores, R. (2003) Peripheral regions of natural hammerhead ribozymes greatly increase their self-cleavage activity, *EMBO J.,* **22,** 5561–5570.
4. Khvorova, A., Lescoute, A., Westhof, E. and Jayasena, S.D. (2003) Sequence elements outside the hammerhead ribozyme catalytic core enable intracellular activity, *Nat. Struct. Biol.,* **10,** 708–712.
5. Martick, M. and Scott, W.G. (2006) Tertiary contacts distant from the active site prime a ribozyme for catalysis, *Cell,* **126,** 309–320.
6. Long, D.M. and Uhlenbeck, O.C. (1994) Kinetic characterization of intramolecular and intermolecular hammerhead RNAs with stem II deletions, *Proc. Natl. Acad. Sci. USA,* **91,** 6977–6981.
7. Garrett, T.A., Pabón-Peña, L.M., Gokaldas, N. and Epstein, L.M. (1996) Novel requirements in peripheral structures of the extended satellite 2 hammerhead, *RNA,* **2,** 699–706.
8. Lünse, C.E., Weinberg, Z. and Breaker, R.R. (2017) Numerous small hammerhead ribozyme variants associated with Penelope-like retrotransposons cleave RNA as dimers, *RNA Biol.,* **14,** 1499-1507.
9. Ferbeyre, G., Smith, J.M. and Cedergren, R. (1998) Schistosome satellite DNA encodes active hammerhead ribozymes, *Mol. Cell Biol.,* **18,** 3880–3888.
10. Jimenez, R.M., Delwart, E. and Lupták, A. (2011) Structure-based search reveals hammerhead ribozymes in the human microbiome, *J. Biol. Chem.,* **286,** 7737–7743.
11. Penedo, J.C., Wilson, T.J., Jayasena, S.D., Khvorova, A. and Lilley, D.M.J. (2004) Folding of the natural hammerhead ribozyme is enhanced by interaction of auxiliary elements, *RNA,* **10,** 880–888.
12. Seehafer, C., Kalweit, A., Steger, G., Gräf, S. and Hammann, C. (2011) From alpaca to zebrafish. Hammerhead ribozymes wherever you look, *RNA,* **17,** 21–26.
13. Canny, M.D., Jucker, F.M., Kellogg, E., Khvorova, A., Jayasena, S.D. and Pardi, A. (2004) Fast cleavage kinetics of a natural hammerhead ribozyme, *J. Am. Chem. Soc.,* **126,** 10848–10849.
14. Saksmerprome, V., Roychowdhury-Saha, M., Jayasena, S., Khvorova, A. and Burke, D.H. (2004) Artificial tertiary motifs stabilize trans-cleaving hammerhead ribozymes under conditions of submillimolar divalent ions and high temperatures, *RNA,* **10,** 1916–1924.
15. Nelson, J.A., Shepotinovskaya, I. and Uhlenbeck, O.C. (2005) Hammerheads derived from sTRSV show enhanced cleavage and ligation rate constants, *Biochemistry,* **44,** 14577–14585.
16. Hertel, K.J., Herschlag, D. and Uhlenbeck, O.C. (1994) A kinetic and thermodynamic framework for the hammerhead ribozyme reaction, *Biochemistry,* **33,** 3374–3385.
17. Cervera, A. and de la Peña, M. (2014) Eukaryotic penelope-like retroelements encode hammerhead ribozyme motifs, *Mol. Biol. Evol.,* **31,** 2941–2947.

18. Shepotinovskaya, I.V. and Uhlenbeck, O.C. (2008) Catalytic diversity of extended hammerhead ribozymes, *Biochemistry,* **47,** 7034–7042.

19. Ambrós, S. and Flores, R. (1998) In vitro and in vivo self-cleavage of a viroid RNA with a mutation in the hammerhead catalytic pocket, *Nucleic Acids Res,* **26,** 1877–1883.

20. Przybilski, R. and Hammann, C. (2007) The tolerance to exchanges of the Watson Crick base pair in the hammerhead ribozyme core is determined by surrounding elements, *RNA,* **13,** 1625–1630.

21. Carbonell, A., de la Peña, M., Flores, R. and Gago, S. (2006) Effects of the trinucleotide preceding the self-cleavage site on eggplant latent viroid hammerheads: differences in co- and post-transcriptional self-cleavage may explain the lack of trinucleotide AUC in most natural hammerheads, *Nucleic Acids Res.,* **34,** 5613–5622.

22. Canny, M.D., Jucker, F.M. and Pardi, A. (2007) Efficient ligation of the Schistosoma hammerhead ribozyme, *Biochemistry,* **46,** 3826–3834.

23. Roth, A., Weinberg, Z., Chen, A.G., Kim, P.B., Ames, T.D. and Breaker, R.R. (2014) A widespread self-cleaving ribozyme class is revealed by bioinformatics, *Nat. Chem. Biol.,* **10,** 56–60.

24. Anderson, M., Schultz, E.P., Martick, M. and Scott, W.G. (2013) Active-site monovalent cations revealed in a 1.55-Å-resolution hammerhead ribozyme structure, *J. Mol. Biol.,* **425,** 3790–3798.
