# Supplementary material for: Discovery of natural non-circular permutations in non-coding RNAs
Source: Nucleic Acids Res. 2023 Mar 13;51(6):2850–61. doi: 10.1093/nar/gkad137 (PMC10085705; doi:10.1093/nar/gkad137)
Supplement: gkad137_Supplemental_Files [file gkad137_supplemental_files.zip › Supplementary-File-3.pdf]

Supplementary File 3
(printable multiple-sequence alignments of motifs found)
Discovery of natural non-circular permutations in non-coding RNAs

Iris Eckert, Richard Friedrich, Christina E. Weinberg, Zasha Weinberg

Note:

- The various alignments presented here are explained in Supplementary Table 2.
- Machine-readable versions of these alignments are available in Stockholm format in Supplementary File 2.
- The presentation and explanatory text of this supplementary data on novel RNA motifs follows the pattern of a presentation of previously found conserved RNA motifs (Weinberg, *et al.*, 2010) and (Weinberg, *et al.*, 2021).

Contents

1 Type-SGC-hammerhead **3**
1.1 Multiple-sequence alignment . . . 3

2 Type-SGC-variant-hammerhead **5**
2.1 Multiple-sequence alignment . . . 5

3 Type-CSG-hammerhead **7**
3.1 Multiple-sequence alignment . . . 7

4 Type-GCS-hammerhead **8**
4.1 Multiple-sequence alignment . . . 8

5 HRIMA-2 **9**
5.1 Multiple-sequence alignment . . . 9

6 HRIMA-3 **10**
6.1 Multiple-sequence alignment . . . 10

7 HRIMA-4 **11**
7.1 Multiple-sequence alignment . . . 11

8 HRIMA-5 **12**
8.1 Multiple-sequence alignment . . . 12

9 HRIMA-6 **14**
9.1 Multiple-sequence alignment . . . 14

10 HRIMA-7 **15**
10.1 Multiple-sequence alignment . . . 15

11 HRIMA-8 **16**
11.1 Multiple-sequence alignment . . . 16

12 HRIMA-9 **17**
12.1 Multiple-sequence alignment . . . 17

13 HRIMA-10 **18**
13.1 Multiple-sequence alignment . . . 18

14 HRIMA-11 **19**
14.1 Multiple-sequence alignment . . . 19

15 HRIMA-12 **20**
15.1 Multiple-sequence alignment . . . 20

16 HRIMA-13 **21**
16.1 Multiple-sequence alignment . . . 21

17 HRIMA-14 **22**
17.1 Multiple-sequence alignment 22

18 HRIMA-15 **23**
18.1 Multiple-sequence alignment 23

19 HRIMA-16 **25**
19.1 Multiple-sequence alignment 25

20 HRIMA-17 **26**
20.1 Multiple-sequence alignment 26

21 HRIMA-18 **27**
21.1 Multiple-sequence alignment 27

22 HRIMA-19 **28**
22.1 Multiple-sequence alignment 28

23 HRIMA-20 **29**
23.1 Multiple-sequence alignment 29

24 HRIMA-21 **30**
24.1 Multiple-sequence alignment 30

25 HRIMA-22 **31**
25.1 Multiple-sequence alignment 31

26 HRIMA-23 **32**
26.1 Multiple-sequence alignment 32

27 HRIMA-24 **33**
27.1 Multiple-sequence alignment 33

28 HRIMA-25 **34**
28.1 Multiple-sequence alignment 34

29 HRIMA-26 **35**
29.1 Multiple-sequence alignment 35

1 Type-SGC-hammerhead

1.1 Multiple-sequence alignment

For a description of this alignment, see Supplementary Table 2.

Each ribozyme in this alignment is identified by its genomic location in the form SEQID/START-END. SEQID (the sequence accession) is derived from sources such as IMG/M or GenBank. START is the coordinate of the 5′ nucleotide of the hairpin ribozyme, and END corresponds to the 3′ nucleotide. If START>END, then the ribozyme is on the reverse complement strand.

Nucleotides proposed to basepair as part of the consensus structure are shaded in color when they comprise Watson-Crick or G-U pairs. Otherwise they are shaded

gray. Conserved stems are also indicated at the bottom of the alignment by angle brackets, where matching < and > denote base-paired columns. Below these angle brackets, the symbol “2” denotes base pairs exhibiting covariation according to the statistically well-founded R-scape method. “1” denotes base pairs exhibiting covariation according to R2R’s simplistic method. “0” denotes base pairs that are not observed to mutate and “?” denotes base pairs that have a significant frequency of non-canonical nucleotides for Watson-Crick or G-U pairs (> 5%). Below these base pair annotation is the consensus sequence: “R” = “A” or “G”, “Y” = “C” or “U”, red nucleotides: nucleotide identity conserved more than 97% of the time, black nucleotides: 90%, gray nucleotides: 75%, red circle (•): nucleotide is present 97% of the time, black circle (•): 90%, gray circle (•): 75%, white circle (∘): 50%. All percentages of sequences just described (e.g. 97% conserved) assume that sequences have been weighted by the GSC algorithm implemented by the Infernal software package.

The alignment begins on the next page.

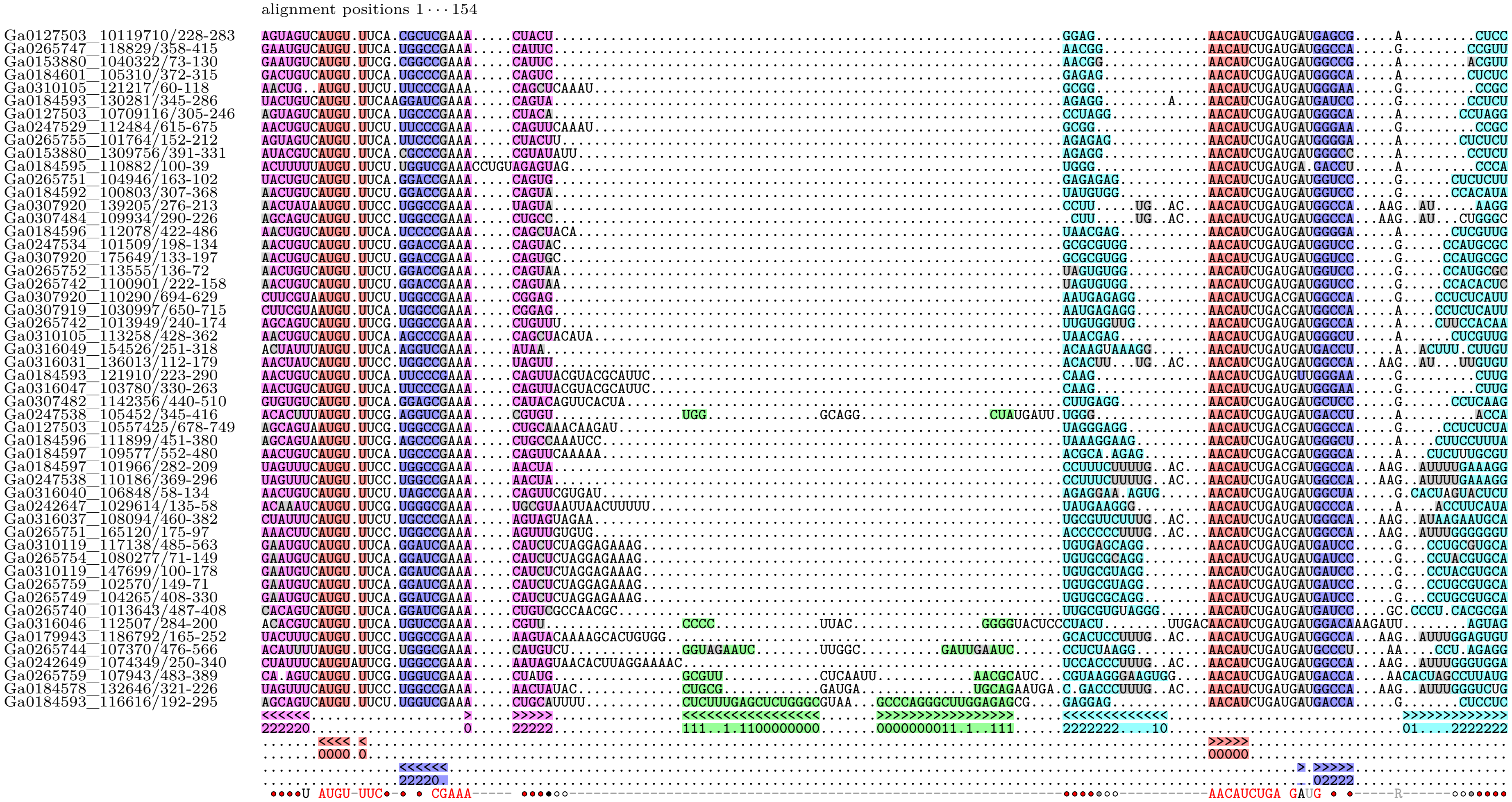

2 Type-SGC-variant-hammerhead

2.1 Multiple-sequence alignment

For a description of this alignment, see Supplementary Table 2.

Each ribozyme in this alignment is identified by its genomic location in the form SEQID/START-END. SEQID (the sequence accession) is derived from sources such as IMG/M or GenBank. START is the coordinate of the 5′ nucleotide of the hairpin ribozyme, and END corresponds to the 3′ nucleotide. If START>END, then the ribozyme is on the reverse complement strand.

Nucleotides proposed to basepair as part of the consensus structure are shaded in color when they comprise Watson-Crick or G-U pairs. Otherwise they are shaded gray. Conserved stems are also indicated at the bottom of the alignment by angle brackets, where matching < and > denote base-paired columns. Below these angle brackets, the symbol "2" denotes base pairs exhibiting covariation according to the statistically well-founded R-scape method. "1" denotes base pairs exhibiting covariation according to R2R's simplistic method. "0" denotes base pairs that are not observed to mutate and "?" denotes base pairs that have a significant frequency of non-canonical nucleotides for Watson-Crick or G-U pairs (> 5%). Below these base pair annotation is the consensus sequence: "R" = "A" or "G", "Y" = "C" or "U", red nucleotides: nucleotide identity conserved more than 97% of the time, black nucleotides: 90%, gray nucleotides: 75%, red circle (•): nucleotide is present 97% of the

time, black circle (•): 90%, gray circle (◦): 75%, white circle (◦): 50%. All percentages of sequences just described (e.g. 97% conserved) assume that sequences have been weighted by the GSC algorithm implemented by the Infernal software package.

The alignment begins on the next page.

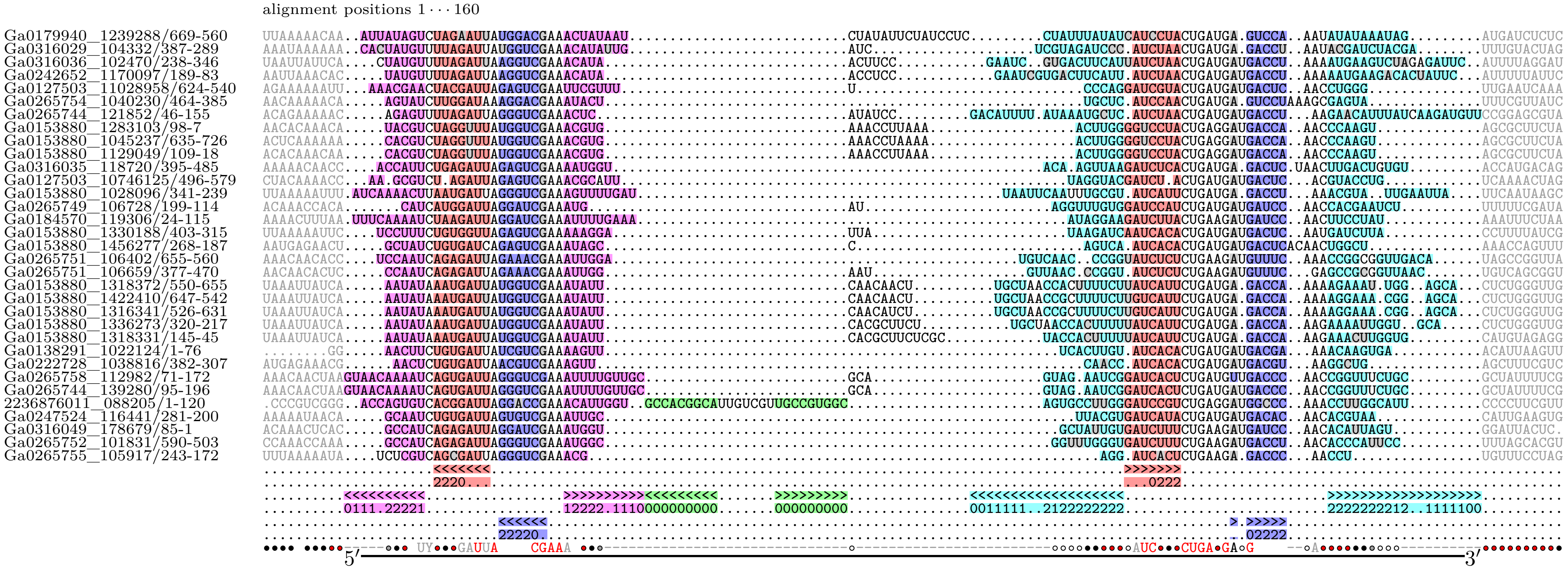

3 Type-CSG-hammerhead

3.1 Multiple-sequence alignment

For a description of this alignment, see Supplementary Table 2.

Each ribozyme in this alignment is identified by its genomic location in the form SEQID/START-END. SEQID (the sequence accession) is derived from sources such as IMG/M or GenBank. START is the coordinate of the 5′ nucleotide of the hairpin ribozyme, and END corresponds to the 3′ nucleotide. If START>END, then the ribozyme is on the reverse complement strand.

Nucleotides proposed to basepair as part of the consensus structure are shaded in color when they comprise Watson-Crick or G-U pairs. Otherwise they are shaded gray. Conserved stems are also indicated at the bottom of the alignment by angle brackets, where matching < and > denote base-paired columns. Below these angle brackets, the symbol "2" denotes base pairs exhibiting covariation according to the statistically well-founded R-scape method. "1" denotes base pairs exhibiting covariation according to R2R's simplistic method. "0" denotes base pairs that are not observed to mutate and "?" denotes base pairs that have a significant frequency of non-canonical nucleotides for Watson-Crick or G-U pairs (> 5%). Below these base pair annotation is the consensus sequence: "R" = "A" or "G", "Y" = "C" or "U", red nucleotides: nucleotide identity conserved more than 97% of the time, black nucleotides: 90%, gray nucleotides: 75%, red circle (•): nucleotide is present 97% of the time, black circle (•): 90%, gray circle (•): 75%, white circle (◦): 50%. All percentages of sequences just described (e.g. 97% conserved) assume that sequences have been weighted by the GSC algorithm implemented by the Infernal software package.

The alignment begins on the next page.

alignment positions 1 · · · 66

```
Ga0102961_1534289/264-329  AAUGCGGUUGGUUGCUGAUGAGGGGUGCCGCAUUAAGUUGUCCAACCAUUAAUACCCCGAAACAAC
Ga0102955_1556782/71-6     AAUGUGGUUGGUUGCUGAUGAGGGUGGCCACAUUCAGUUGUCCAACCAUUAAACACCCGAAACAAC
                           ........<<<<<<............................>>>>>>..................
                           ........000000............................000000..................
                           <<<<<<<<..................>>>>>>>>..<<<<<....................>>>>>
                           00001000..................00010000..00000....................00000
                           ....................<<<<<<...........................>>>>>>.......
                           ....................00011...........................11000........
                           AAUGYGGUUGGUUGCUGAUGAGGG••GCCRCAUU•AGUUGUCCAACCAUUAA•••CCCGAAACAAC
                           5′
```

4 Type-GCS-hammerhead

4.1 Multiple-sequence alignment

For a description of this alignment, see Supplementary Table 2.

Each ribozyme in this alignment is identified by its genomic location in the form SEQID/START-END. SEQID (the sequence accession) is derived from sources such as IMG/M or GenBank. START is the coordinate of the 5′ nucleotide of the hairpin ribozyme, and END corresponds to the 3′ nucleotide. If START>END, then the ribozyme is on the reverse complement strand.

Nucleotides proposed to basepair as part of the consensus structure are shaded in color when they comprise Watson-Crick or G-U pairs. Otherwise they are shaded gray. Conserved stems are also indicated at the bottom of the alignment by angle brackets, where matching $<$ and $>$ denote base-paired columns. Below these angle brackets, the symbol “2” denotes base pairs exhibiting covariation according to the statistically well-founded R-scape method. “1” denotes base pairs exhibiting covariation according to R2R’s simplistic method. “0” denotes base pairs that are not observed to mutate and “?” denotes base pairs that have a significant frequency of non-canonical nucleotides for Watson-Crick or G-U pairs ($>5\%$). Below these base pair annotation is the consensus sequence: “R” = “A” or “G”, “Y” = “C” or “U”, red nucleotides: nucleotide identity conserved more than 97% of the time, black nucleotides: 90%, gray nucleotides: 75%, red circle (•): nucleotide is present 97% of the time, black circle (•): 90%, gray circle (•): 75%, white circle (◦): 50%. All percentages of sequences just described (e.g. 97% conserved) assume that sequences have been weighted by the GSC algorithm implemented by the Infernal software package.

The alignment begins on the next page.

alignment positions $1 \cdots 51$

```
NZ_FCOH02000029.1/62379-62429   GACCGAAACGCACUGUUGCGCGAUCUGAUGAGGUCCAGUGCGUCGUUGCGC
                                <<<<...........................>>>>................
                                0000...........................0000................
                                .......<<<<<<<<....................>>>>>>>>........
                                .......00000000....................00000000........
                                .................<<<<<<<....................>>>>>>>
                                .................0000000....................0000000
                                GACCGAAACGCACUGUUGCGCGAUCUGAUGAGGUCCAGUGCGUCGUUGCGC
```

5 HRIMA-2

5.1 Multiple-sequence alignment

For a description of this alignment, see Supplementary Table 2.

Each ribozyme in this alignment is identified by its genomic location in the form SEQID/START-END. SEQID (the sequence accession) is derived from sources such as IMG/M or GenBank. START is the coordinate of the 5′ nucleotide of the hairpin ribozyme, and END corresponds to the 3′ nucleotide. If START>END, then the ribozyme is on the reverse complement strand.

Nucleotides proposed to basepair as part of the consensus structure are shaded in color when they comprise Watson-Crick or G-U pairs. Otherwise they are shaded gray. Conserved stems are also indicated at the bottom of the alignment by angle brackets, where matching $<$ and $>$ denote base-paired columns. Below these angle brackets, the symbol “2” denotes base pairs exhibiting covariation according to the statistically well-founded R-scape method. “1” denotes base pairs exhibiting covariation according to R2R’s simplistic method. “0” denotes base pairs that are not observed to mutate and “?” denotes base pairs that have a significant frequency of non-canonical nucleotides for Watson-Crick or G-U pairs ($> 5\%$). Below these base pair annotation is the consensus sequence: “R” = “A” or “G”, “Y” = “C” or “U”, red nucleotides: nucleotide identity conserved more than 97% of the time, black nucleotides: 90%, gray nucleotides: 75%, red circle (•): nucleotide is present 97% of the time, black circle (•): 90%, gray circle (•): 75%, white circle (◦): 50%. All percentages of sequences just described (e.g. 97% conserved) assume that sequences have been weighted by the GSC algorithm implemented by the Infernal software package.

The alignment begins on the next page.

alignment positions 1 · · · 106

```
Ga0179943_1203640/527-425  GUCGCAGAAA.UAUGUUCAGGCAUAG.UUUCUACAGUAUCUGGUCUUAUGGCACAGCCAAACUUGGCUCUCACCCUGUGUCGACCUAUCUAACCAC.GCGACUUUC
Ga0179947_1048335/302-403  GUCGCAGAAA.UAUGUUCAGGCAUAG.UUUCUUCAGUAUCUGGUCAUAAUGCAUAGCCAAACUUGGCUCUCAC..UGUGCAGACCUAUCUAAACAAAGCGACUUUC
Ga0179955_1089777/330-427  GGAGCAAAGG.AGUCUUCAGGGACUG.CCUGUUCAGUAUCUGGUUAUUUCGGGAAAACU...UAGCCUCUCA...CCCGUAGGCCUAUCUGAUCAUCGCUCCAUUC
Ga0179954_1173557/438-338  GUAGUCAAGACGCUCUUCAGGGAGCGGUCUGUUCAGUAUCUUGUUAUUUUGGCGAAACUA.UUACUCUCUAA...GCCAUAGGCAUAUCUGAUCAU.ACUACAUUC
Ga0179955_1202528/758-658  GUAGUUAAGACGCUCUUCAGGGAGCGGUCUGUUCAGUAUCUUGUUAUUUUGGCGAAACUA.UUACUCUCUAA...GCCAUAGGCAUAUCUGAUCAU.ACUACAUUC

                           <<<<<..<<<<<<<<<....>>>>>>.>>>...........<<<<....<<<<.....................>>>>..>>>>............>>>>>....
                           02101..011.22010....01022..110...........20......12.....................21......02............10120....
                           G••GY•RARR-••U•UUCAGG•A••G-YYU•U CAGUAUCU•GUY U•  G•••AR•C•○-○U•••CUCU•A---••YR  GRC•UAUCURA CA -RC••C•UUC
                           5′
```

6 HRIMA-3

6.1 Multiple-sequence alignment

For a description of this alignment, see Supplementary Table 2.

Each ribozyme in this alignment is identified by its genomic location in the form SEQID/START-END. SEQID (the sequence accession) is derived from sources such as IMG/M or GenBank. START is the coordinate of the 5′ nucleotide of the hairpin ribozyme, and END corresponds to the 3′ nucleotide. If START>END, then the ribozyme is on the reverse complement strand.

Nucleotides proposed to basepair as part of the consensus structure are shaded in color when they comprise Watson-Crick or G-U pairs. Otherwise they are shaded gray. Conserved stems are also indicated at the bottom of the alignment by angle brackets, where matching < and > denote base-paired columns. Below these angle brackets, the symbol "2" denotes base pairs exhibiting covariation according to the statistically well-founded R-scape method. "1" denotes base pairs exhibiting covariation according to R2R's simplistic method. "0" denotes base pairs that are not observed to mutate and "?" denotes base pairs that have a significant frequency of non-canonical nucleotides for Watson-Crick or G-U pairs (> 5%). Below these base pair annotation is the consensus sequence: "R" = "A" or "G", "Y" = "C" or "U", red nucleotides: nucleotide identity conserved more than 97% of the time, black nucleotides: 90%, gray nucleotides: 75%, red circle (•): nucleotide is present 97% of the time, black circle (•): 90%, gray circle (•): 75%, white circle (○): 50%. All percentages of sequences just described (e.g. 97% conserved) assume that sequences have been weighted by the GSC algorithm implemented by the Infernal software package.

The alignment begins on the next page.

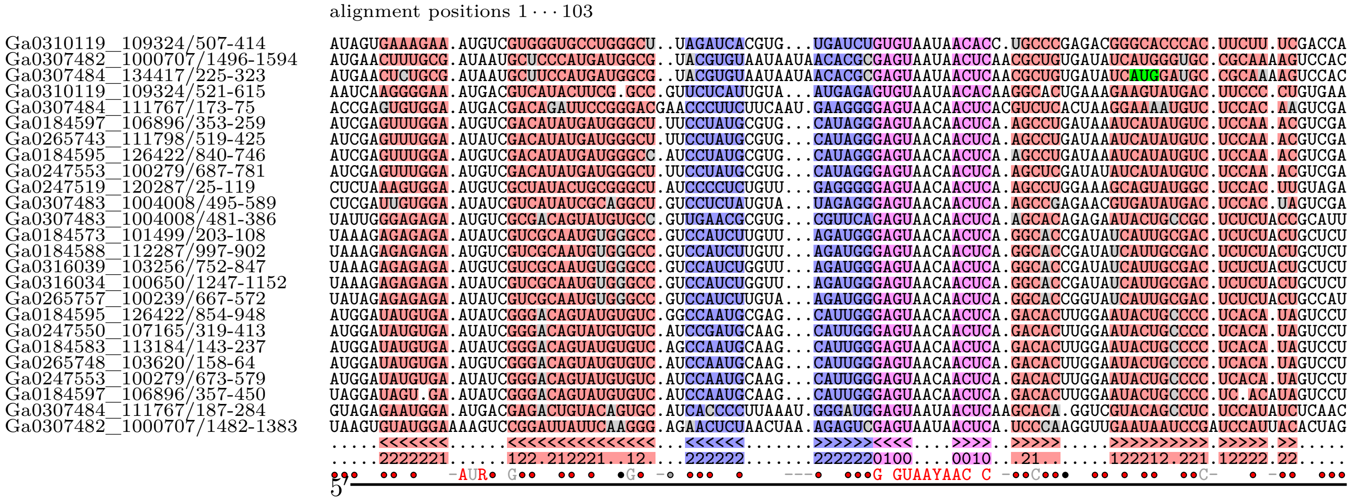

7 HRIMA-4

7.1 Multiple-sequence alignment

For a description of this alignment, see Supplementary Table 2.

Each ribozyme in this alignment is identified by its genomic location in the form SEQID/START-END. SEQID (the sequence accession) is derived from sources such as IMG/M or GenBank. START is the coordinate of the 5′ nucleotide of the hairpin ribozyme, and END corresponds to the 3′ nucleotide. If START>END, then the ribozyme is on the reverse complement strand.

Nucleotides proposed to basepair as part of the consensus structure are shaded in color when they comprise Watson-Crick or G-U pairs. Otherwise they are shaded gray. Conserved stems are also indicated at the bottom of the alignment by angle brackets, where matching < and > denote base-paired columns. Below these angle brackets, the symbol "2" denotes base pairs exhibiting covariation according to the statistically well-founded R-scape method. "1" denotes base pairs exhibiting covariation according to R2R's simplistic method. "0" denotes base pairs that are not observed to mutate and "?" denotes base pairs that have a significant frequency of non-canonical nucleotides for Watson-Crick or G-U pairs (> 5%). Below these base pair annotation is the consensus sequence: "R" = "A" or "G", "Y" = "C" or "U", red nucleotides: nucleotide identity conserved more than 97% of the time, black nucleotides: 90%, gray nucleotides: 75%, red circle (•): nucleotide is present 97% of the time, black circle (•): 90%, gray circle (•): 75%, white circle (◦): 50%. All percentages of sequences just described (e.g. 97% conserved) assume that sequences have been weighted by the GSC algorithm implemented by the Infernal software package.

The alignment begins on the next page.

alignment positions 1 · · · 107

```
Ga0247531_122047/236-134    CCUUUGAC.UAGGAUACGACCGUAACU..CUUCGUGAG.ACGGGCAAAUCCGAGGUCUCUCUGGAGUAAUUCAGAGUGUAGUUGAAGGUCCGAACAAUGAGGCCU
Ga0247536_100773/1002-899   CUCCGAGCCAUGGAUACAGCCAGAGACUGCAUAGCAGU.CUGGAUAAAUCCUGUAGGUGUCACUGGAGUAAUUCAGUG.GU.GCUCUGGAGUCGUACGACGACCUGC
Ga0184578_101068/115-218    CUCCGAGCCUUGGAUACAGCCAGAGACUGCAUAGCAGU.CUGGAUAAAUCCUGUAGGUGUCACUGGAGUAAUUCAGUG.GU.GCUCUGGAGUCGUACAACGACCUGC
Ga0265756_100592/1093-1196  CUCCGAGCCAUGGAUACGGCCAGAGACUGCAUAGCAGU.CUGGACAAAUCCUGUAGGUUUCACUGGAGUAAUUCAGUG.GU.GCUCUGGAGUCGUACAAAGACCUGC
Ga0184578_107237/66-169     CUCCGAGCCAUGGAUACGGCCAGAGACUGCAUAGCAGU.CUGGACAAAUCCUGUAGGUGACACUGGAGUAAUUCAGUG.GU.GCUUAGGAGUCGUACAACGACCUGC
Ga0247551_100191/543-646    CUCCGAGCCAUGGAUACGGCCAGAGACUGCAUAGCAGU.CUGGACAAAUCCUGUAGGUGACACUGGAGUAAUUCAGUG.GU.GCUUUGGAGUCGUACAACGACCUGC
Ga0247535_100426/388-286    CUCCGAGCCAUGGAUACGGCCAGAGACUGCAUAGCAGU.CUGGACAAAUCCUGUAGGUGACACUGGAGUAAUUCAGUG.GU.GCU.UGGAGUCGUACAACGACCUGC
Ga0247518_129144/190-87     CUCCGAGCCAUGGAUACGGCCAGAGACUGCAUAGCAGU.CUGGACAAAUCCUGUAGGUGUCACUGGAGUAAUUCAGUG.GU.GCUCUGGAGUCGUACAACGACCUGC
Ga0247517_137551/194-296    CUCCGAGCCAUGGAUACGGCCAGAGACUGCAUAGCAGU.CUGGACAAAUCCUGUAGGUGACACUGGAGUAAUUCAGUG.GU.GCUCU.GAGUCGUACAACGACCUGC
Ga0247530_109035/125-228    CUCCGAGCCAUGGAUACGGCCAGAGACUGCAUAGCAGU.CUGGACAAAUCCUGCAGGUGUCACUGGAGUAAUUCAGUG.GU.GCUCUGGAGUCGUACAACGACCUGU
                            ....................................................<<<<<<...........................................>>>>>>
                            ....................................................111110...........................................011111
                            <<<<<<<<...<<<<....<<<<..<<<<<...>>>>>.>>>>....>>>>.........<<<<<<<....>>>>>>>....>>>>.>>>>................
                            .1.11.10...0000....0011..11..0...0..11.1100....0000.........0100000....0000010....01.1.1.1.................
                            CYYY•RRC○••GGAUAC RCCR•AR•Y○○C•U•GYRR•-•YGGR AAAUCC•R•R••U• C•CUGGAGUAAUUCAG•G-GU-GYU••RGR•YCG•ACAAYGA••Y•Y
                            5′
```

8 HRIMA-5

8.1 Multiple-sequence alignment

For a description of this alignment, see Supplementary Table 2.

Each ribozyme in this alignment is identified by its genomic location in the form SEQID/START-END. SEQID (the sequence accession) is derived from sources such as IMG/M or GenBank. START is the coordinate of the 5′ nucleotide of the hairpin ribozyme, and END corresponds to the 3′ nucleotide. If START>END, then the ribozyme is on the reverse complement strand.

Nucleotides proposed to basepair as part of the consensus structure are shaded in color when they comprise Watson-Crick or G-U pairs. Otherwise they are shaded gray. Conserved stems are also indicated at the bottom of the alignment by angle brackets, where matching $<$ and $>$ denote base-paired columns. Below these angle brackets, the symbol "2" denotes base pairs exhibiting covariation according to the statistically well-founded R-scape method. "1" denotes base pairs exhibiting covariation according to R2R's simplistic method. "0" denotes base pairs that are not observed to mutate and "?" denotes base pairs that have a significant frequency of non-canonical nucleotides for Watson-Crick or G-U pairs ($> 5\%$). Below these base pair annotation is the consensus sequence: "R" = "A" or "G", "Y" = "C" or "U", red nucleotides: nucleotide identity conserved more than 97% of the time, black nucleotides: 90%, gray nucleotides: 75%, red circle (•): nucleotide is present 97% of the time, black circle (•): 90%, gray circle (•): 75%, white circle (○): 50%. All percentages of sequences just described (e.g. 97% conserved) assume that sequences have been weighted by the GSC algorithm implemented by the Infernal software package.

The alignment begins on the next page.

alignment positions 1 · · · 49

```
Ga0247546_116439/63-292      UCAACGGCAAAUUAAGAGGGAAACCUGAGGAGGCCUUAACUGUGCCACA
Ga0247538_100551/818-1047    UCAACGGCAAAUUAAGAGGGAAACCUGAGGAGGCCUUAACUGUGCCACA
Ga0247536_101401/424-653     UCAACGGCAAAUUAAGAGGGAAACCUGAGGAGGCCUUAACUGUGCCACA
Ga0194131_10516571/311-126   GGGCUUGUACCCUCUGCGAAUAUCAUGGGUCACCUUUUGAGGCACUGCA
Ga0247554_114792/1-149       ......................................ACUGUGCCACA
Ga0247538_100524/174-360     AGAAACUAUGUUGUGGGAACUAAAGUGAGGAGGCCUUAACUGUGCCACA
Ga0247520_105734/445-239     GGAAACUACGUUUAAGGAGUCAAAGUGAGGAGGCCUUUACUGUGCCACA
Ga0247542_100681/783-988     GGAAACUACGUUUAAGGAGUCAAAGUGAGGAGGCCUUUACUGUGCCACA
Ga0247515_107373/453-247     GGAAACUACGUUUAAGGAGUCAAAGUGAGGAGGCCUUUACUGUGCCACA
Ga0247554_100355/809-1015    GGAAACUACGUUUAAGGAGUCAAAGUGAGGAGGCCUUUACUGUGCCACA
Ga0247553_100761/463-253     CAAGUACCGUGUUCAGGUGUCAAAGUGAGGAGGCCUUAACUGUGCCACA
Ga0316053_101091/188-398     CAAGUACCGUGUUCAGAUGUCAAAGUGAGGAGGCCUUAACUGUGCCACA
Ga0247526_105246/780-990     CAAGUACCGUGUUCAGAUGUUAAAGUGAGGAGGCCUUAACUGUGCCACA
Ga0247551_100244/228-18      CAAGUACCGUGUUCAGAUGUUAAAGUGAGGAGGCCUUAACUGUGCCACA
Ga0316047_100743/1381-1182   ...........UUCAGAUGUUAAAGUGAGGAGGCCUUAACUGUGCCACA
Ga0247555_109146/1-161       .................................................
Ga0247543_100271/1-188       .......................AGUGAGGAGGCCUUAACUGUGCCACA
Ga0247545_100371/462-252     CAAGUACCGUGUUCAGGUGUCAAAGUGAGGAGGCCUUAACUGUGCCACA
Ga0247531_104937/459-250     CAAGUACCGUGUUCAGAUGUCAAAGUGAGGAGGCCUUAACUGUGCCACA
Ga0247539_104607/443-237     ....UACCGUGUUCAGAUGUCAAAGUGAGGAGGCCUUAACUGUGCCACA
Ga0184593_133703/447-237     CAAGUACCGUGUUCAGGUGUCAAAGUGAGGAGGCCUUAACUGUGCCACA
Ga0316052_100844/976-766     CAAGUACCGUGUUCAGAUGUCAAAGUGAGGAGGCCUUAACUGUGCCACA

                             .................................................
                             .................................................
                             ○○ ○○○○○○○○Y ●●G●●R●●A●●●UGRG●●R●CYUU●   G  C  CA
```

alignment positions 50 · · · 209

```
Ga0247546_116439/63-292      AGGUGCUUCAAUC.CGAGGAAC.UAACGCU.CCAAGGAGGGAAAG..UCCCGUUCUCGCGGUUCCGUACCGGCAAGAUCCGGGGGCCGAGGGCGGUUUGAAACCUGCACUCACGUGUCCAAAACGAAACUACCUCUGAAAGUUGUGUCAGUCCAGCGUGG
Ga0247538_100551/818-1047    AGGUGCUUCAAUC.CGAGGAAC.UAACGCU.CCUAGGAGGGAAAG..UCCCGUUCUCGCGGUUCCGUACCGGCAAGAUCCGGGGGCCGAGGGCGGUUUGAAACCUGCACUCACGUGUCCAAAAUGAAACUACCUCUGAAAGUUGUGUCAGUCCAGCGUGG
Ga0247536_101401/424-653     AGGUGCUUCAAUC.CGAGGAAC.UAACGCU.CCUAGGAGGGAAAG..UCCCGUUCUCGCGGUUCCGUACCGGCAAGAUCCGGGGGCCGAGGGCGGUUUGAAACCUGCACUCACGUGUCCAAAAAGAAAGUACCUUUGAAAGUUGUGUCAGUCCAGCGUGG
Ga0194131_10516571/311-126   AAAGGUUUCAAA.......C.................UGAGCAAG..GGCUACAUUGGUUUUAGG......................CAAUGUAGUAGUCGAACUGCCUAGACAAGUCCAAGAAAAAAUUC.UCUUAAUGUUCACCAUUUGAAUGAAAUU
Ga0247554_114792/1-149       AGAUGCUUCAACCCCCGGGAA.CUAAUGCA.CCCGGAGGAGUGU.UCUU........U.....................................UUUAAGACCCGCAUCAGCAUGUCCAAAAACAAGUAACUUCCACUUCACAAGCCUAAUCGUAGUGC
Ga0247538_100524/174-360     AGAUGCUUCAACCCCCGGGAA.CUAAUGCA.CCCGGAGGAGUGU.UCUU........U.....................................UUUAAGACCCGCAUCAGCAUGUCCAAAAACAAGUAACUUCCACUUCACAAGCCUAAUCGUAGUGC
Ga0247520_105734/445-239     AGACGCUUCAACC.UCUGGGGGUUAAUGCAACCGGAG.GAGUAAACCUGCUGCGGUGGAUG..........................CACUGUAGUUUCAGACCCGCGUCAGCAUGUCCAAAAACAAGUACCUUCUUAAUACGCAAGCCCGUGCGUUGAU
Ga0247542_100681/783-988     AGACGCUUCAACC.UCUGGGG.UUAAUGCAACCGGAG.GAGUAAACCUGCUGCGGUGGAUG..........................CACUGUAGUUUCAGACCCGCGUCAGCAUGUCCAAAAACAAGUACCUUCUUAAUACGCAAGCCCGUGCGUUGAU
Ga0247515_107373/453-247     AGACGCUUCAACC.UCUGGGGGUUAAUGCAACCGGAG.GAGUAAACCUGCUGCGGUGGAAG..........................CACUGUAGUUUCAGACCCGCGUCAGCAUGUCCAAAAACAAGUACCUUCUUUUAUACGCAAGCCCGUGCGUUGA
Ga0247554_100355/809-1015    AGACGCUUCAACC.UCUGGGGGUUAAUGCAACCGGAG.GAGUAAACCUGCUGCGGUGGAAG..........................CACUGUAGUUUCAGACCCGCGUCAGCAUGUCCAAAAACAAGUACCUUCUUAAUACGCAAGCCCGUGCGUUGAU
Ga0247553_100761/463-253     AGACGCUUCAACUUUCCGGAAG.UAAUGCA.CCGGAUUGAGUAAAGUUUCUGCGUUGGAUGUAAG......................CAAUGUAGUUUAAACCCCGCGUCAGCAUGUCCAAGAAACAAGUACCGUCUGUUUGGCUGUGCCCUGUAUCGCG
Ga0316053_101091/188-398     AGACGCUUCAACUUUCCGGAAG.UAAUGCA.CCGGAUUGAGUAAAGUUUCUGCGUUGGAUGUAAG......................CAAUGUAGUUUAAACCCCGCGUCAGCAUGUCCAAGAAACAAGUACCGUCUGUUUGGCUGUGCCCUGUAUCGCG
Ga0247526_105246/780-990     AGACGCUUCAACUUUCCGGAAG.UAAUGCA.CCGGAUUGAGUAAAGUUUCUGCGUUGGAUGUAAG......................CAAUGUAGUUUAAACCCCGCGUCAGCAUGUCCAAGAAACAAGUACCGUCUGUUUGGCUGUGCCCUGUAUCGCG
Ga0247551_100244/228-18      AGACGCUUCAACUUUCCGGAAG.UAAUGCA.CCGGAUUGAGUAAAGUUUCUGCGUUGGAUGUAAG......................CAAUGUAGUUUAAACCCCGCGUCAGCAUGUCCAAGAAACAAGUACCGUCUGUUUGGCUGUGCCCUGUAUCGCG
Ga0316047_100743/1381-1182   AGACGCUUCAACUUUCCGGAAG.UAAUGCA.CCGGAUUGAGUAAAGUUUCUGCGUUGGAUGUAAG......................CAAUGUAGUUUAAACCCCGCGUCAGCAUGUCCAAGAAACAAGUACCGUCUGUUUGGCUGUGCCCUGUAUUGCG
Ga0247555_109146/1-161       .GACGCUUCAACUUUCCGGAAG.UAAUGCA.CCGGAUUGAGUAAAGUUUCUGCGUUGGAUGUAAG......................CAAUGUAGUUUAAACCCCGCGUCAGCAUGUCCAAGAAACAAGUACCGUCUGUUUGGCUGUGCCCUGUAUCGCG
Ga0247543_100271/1-188       AGACGCUUCAACUUUCCGGAAG.UAAUGCA.CCGGAUUGAGUAAAGUUUCUGCGUUGUAUGUAAG......................CAAUGUAGUUUAAACCCCGCGUCAGCAUGUCCAAGAAACAAGUACCGUCUGUUUGGCUGUGCCCUGUAUCGCG
Ga0247545_100371/462-252     AGACGCUUCAACUUUCCGGAAG.UAAUGCA.CCGGAUUGAGUAAAGUUUCUGCGUUGUAUGUAAG......................CAAUGUAGUUUAAACCCCGCGUCAGCAUGUCCAAGAAACAAGUACCGUCUGUUUGGCUGUGCCCUGUAUCGCG
Ga0247531_104937/459-250     AGACGCUUCAACUUUCCGGAAG.UAAUGCG.CCGGAUUGAGUAAAGUUUCUGCGUUGUAUGUAAG......................CAAUGUAGUUUAAACCCCGCGUCAGCAUGUCUAAAAACAAGUAC.CGUCUGUUUAGCUGUGCCCUGUAUCGCG
Ga0247539_104607/443-237     AGACGCUUCAACUUUCCGGAAG.UAAUGCA.CCGGAUUGAGUAAAGUUUCUGCGUUGUAUGUAAG......................CAAUGUAGUUUAAACCCCGCGUCAGCAUGUCCAAAAACAAGUACUCAUCUGUUUGCUGUGCCCUGUAUCGCGC
Ga0184593_133703/447-237     AGACGCUUCAACUUUCCGGAAG.UAAUGCA.CCGGAUUGAGUAAAGUUUCUGCGUUGUAUGUAAG......................CAAUGUAGUUUAAACCCCGCGUCAGCAUGUCCAAAAACAAGUACUCAUCUGUUUGCUGUGCCCUGUAUCGCGC
Ga0316052_100844/976-766     AGACGCUUCAACUUUCCGGAAG.UAAUGCG.CCGGAUUGAGUAAAGUUUCUGCGUUGUAUGUAAG......................CAAUGUAGUUUAAACCCCGCGUCAGCAUGUCCAAAAACAAGUACUCAUCUGUUUGCUGUGCCCUGUAUCGCGC

                             .<<<<<.........<<<<<............>>>>>.........<<<<<<<<<<<<..............................>>>>>>>>...>>>>...>>>>>..................................................
                             ..110..........1.100............001.1.........  11011.1201..............................1021.110...11......011...................................................
                             A  G UUCAA  –  ○○    ○–      ○–    ○   ○○G  G    ○○  ●     ○○  ○○○○○○○○○––––––––––––––––––––○  ○○     U   ● R● CYGC    RC   GUCCAARA  ●  AR  ●●○Y●YY●  ●●●●●●●●●●  ●●●●●●●●●●●
                              5′                                                                                                                                                3′
```

alignment positions 210 · · · 235

```
Ga0247546_116439/63-292      AUAACCGUCGUCUUGGAGCCAGUCUU
Ga0247538_100551/818-1047    AUAACCGUCGUCUUGGAGCCAGUCUU
Ga0247536_101401/424-653     AUAACCUUCGUCUUGGAGCCAGUCUU
Ga0194131_10516571/311-126   GAAAAACGUUAAAUACUUUCCCCAAA
Ga0247554_114792/1-149       CUCGUCUUGGUACCGGUCACUUCUGG
Ga0247538_100524/174-360     CUCGUCUUGGUACCGGUCACUUCUGG
Ga0247520_105734/445-239     UCUCUCUCUUGGACUGGUCAUCUUCU
Ga0247542_100681/783-988     UCUCUCUCUUGGACUGGUCAUCUUCU
Ga0247515_107373/453-247     UUCUCUCUCUUGGACUGGUCAUCUUC
Ga0247554_100355/809-1015    UCUCUCUCUUGGACUGGUCAUCUUCU
Ga0247553_100761/463-253     CGCUAUCUCCUUUACACCGGUCACAU
Ga0316053_101091/188-398     CGCUAUCUCCUUUACACCGGUCACAU
Ga0247526_105246/780-990     CGCUAUCUCUUACACCGGUCACAUUC
Ga0247551_100244/228-18      CGCUAUCUCCUUUACACCGGUCACAU
Ga0316047_100743/1381-1182   CGCUAUCUCCUUUACACCGGUCACAU
Ga0247555_109146/1-161       CGCUAUCUCCUUUACACCGGUCACAU
Ga0247543_100271/1-188       CGCUAUCUCCUUUACACCGGUCACAU
Ga0247545_100371/462-252     CGCUAUCUCCUUUACACCGGUCACAU
Ga0247531_104937/459-250     CGCUAUCUCUUACACCGGUCACAUUC

                             ..........................
                             ..........................
                             ●●●●●    ●●●●●●●●● ●●●   ●●
```

```
Ga0247539__104607/443-237    GCUAUCUCUUACACCGGUCACAUUCG
Ga0184593__133703/447-237    GCUAUCUCUUACACCGGUCACAUUCG
Ga0316052__100844/976-766    GCUAUCUCUUACACCGGUCACAUUCG

                             ..........................
                             ..........................
```

9 HRIMA-6

9.1 Multiple-sequence alignment

For a description of this alignment, see Supplementary Table 2.

Each ribozyme in this alignment is identified by its genomic location in the form SEQID/START-END. SEQID (the sequence accession) is derived from sources such as IMG/M or GenBank. START is the coordinate of the 5′ nucleotide of the hairpin ribozyme, and END corresponds to the 3′ nucleotide. If START>END, then the ribozyme is on the reverse complement strand.

Nucleotides proposed to basepair as part of the consensus structure are shaded in color when they comprise Watson-Crick or G-U pairs. Otherwise they are shaded gray. Conserved stems are also indicated at the bottom of the alignment by angle brackets, where matching < and > denote base-paired columns. Below these angle brackets, the symbol "2" denotes base pairs exhibiting covariation according to the statistically well-founded R-scape method. "1" denotes base pairs exhibiting covariation according to R2R's simplistic method. "0" denotes base pairs that are not observed to mutate and "?" denotes base pairs that have a significant frequency of non-canonical nucleotides for Watson-Crick or G-U pairs (> 5%). Below these base pair annotation is the consensus sequence: "R" = "A" or "G", "Y" = "C" or "U", red nucleotides: nucleotide identity conserved more than 97% of the time, black nucleotides: 90%, gray nucleotides: 75%, red circle (•): nucleotide is present 97% of the time, black circle (•): 90%, gray circle (•): 75%, white circle (◦): 50%. All percentages of sequences just described (e.g. 97% conserved) assume that sequences have been weighted by the GSC algorithm implemented by the Infernal software package.

The alignment begins on the next page.

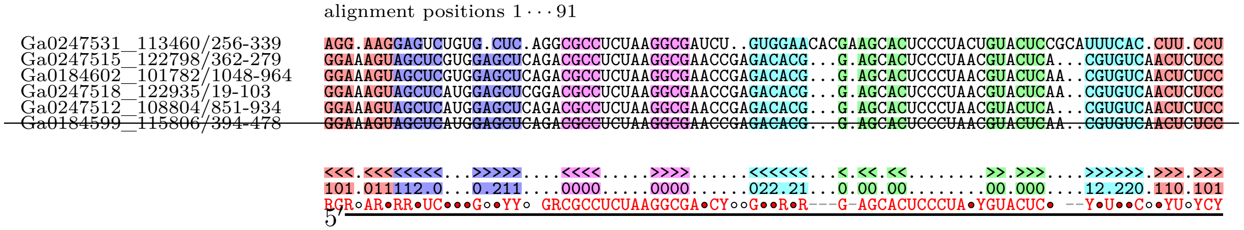

10 HRIMA-7

10.1 Multiple-sequence alignment

For a description of this alignment, see Supplementary Table 2.

Each ribozyme in this alignment is identified by its genomic location in the form SEQID/START-END. SEQID (the sequence accession) is derived from sources such as IMG/M or GenBank. START is the coordinate of the 5′ nucleotide of the hairpin ribozyme, and END corresponds to the 3′ nucleotide. If START>END, then the ribozyme is on the reverse complement strand.

Nucleotides proposed to basepair as part of the consensus structure are shaded in color when they comprise Watson-Crick or G-U pairs. Otherwise they are shaded gray. Conserved stems are also indicated at the bottom of the alignment by angle brackets, where matching < and > denote base-paired columns. Below these angle brackets, the symbol "2" denotes base pairs exhibiting covariation according to the statistically well-founded R-scape method. "1" denotes base pairs exhibiting covariation according to R2R's simplistic method. "0" denotes base pairs that are not observed to mutate and "?" denotes base pairs that have a significant frequency of non-canonical nucleotides for Watson-Crick or G-U pairs (> 5%). Below these base pair annotation is the consensus sequence: "R" = "A" or "G", "Y" = "C" or "U", red nucleotides: nucleotide identity conserved more than 97% of the time, black nucleotides: 90%, gray nucleotides: 75%, red circle (•): nucleotide is present 97% of the time, black circle (•): 90%, gray circle (•): 75%, white circle (◦): 50%. All percentages of sequences just described (e.g. 97% conserved) assume that sequences have been weighted by the GSC algorithm implemented by the Infernal software package.

The alignment begins on the next page.

alignment positions 1 · · · 70

```
Ga0265745__1000512/618-678     GCAACGAGUUGCGCGCCCCGAAAGGGUUUGUCGCGCACAGAA....GGC.GAAA..GCC..AUGAGUUGU
Ga0157371__10683492/53-113     GCAGCGCGCUGCGCGUCGAAUGUGUG.....CGCGCAAAGUUUG..AUC.GUCUU.GAUGGAUGAGCUGU
Ga0157371__12589929/303-363    GCAGCGCGGUGCGCGCCGAAUGUGUG.....CGCGCAAAGUUUG..AUC.GUCUG.GAUGGAUGAGCUGU
Ga0310117__104105/592-647      GCAGCGAGCUGCGCGCCCCAGUGAGC.....CGCGCACAGAU....UU..CUAA...AACUCUGAGCUGU
Ga0265745__1001084/437-492     ACAGCGAGCUGCGCGCCAAAGAUGC......CGCGCACAGAUU.ACC...GAAA....GGUUUGAGCUGU
Ga0265745__1000046/2471-2532   GUGGCGGGUUGCGCGCUCGUAUUGU......CGCGCACAGAUC.ACUUCUUUG.AGAAGUGUGAGCCAU
Ga0265757__100063/2261-2322    GCAGCGUGUUGCGCGCUCGUAAUGC......CGCGCACAGAUC.ACUUCUUUG.AGAAGUGUGAGCUGU
Ga0265745__1000697/1041-981    GCAGCGAGCUGCGCGCCAAAGAAGC......CGCGCACAGAUC.ACUUGUACU..ACAAGUGUGAGUUGU
Ga0265757__100361/1077-1137    GCAACGAGUUGCGCGCCAAAGUAGC......CGCGCACAGAUG.ACUGUGUAU..CACAGUUUGAGUUGU

                               <<<<<....<<<<<<...............>>>>>>.......<<<<<<.....>>>>>>....>>>>>
                               .1110....000000...............000000.........2221.....1222.......0111.
                            5′ GARCG•GYUGCGCGC ••  •• •------CGCGCA AG  ○-○○•••-••••--••••••UGAGYUGU
```

11 HRIMA-8

11.1 Multiple-sequence alignment

For a description of this alignment, see Supplementary Table 2.

Each ribozyme in this alignment is identified by its genomic location in the form SEQID/START-END. SEQID (the sequence accession) is derived from sources such as IMG/M or GenBank. START is the coordinate of the 5′ nucleotide of the hairpin ribozyme, and END corresponds to the 3′ nucleotide. If START>END, then the ribozyme is on the reverse complement strand.

Nucleotides proposed to basepair as part of the consensus structure are shaded in color when they comprise Watson-Crick or G-U pairs. Otherwise they are shaded gray. Conserved stems are also indicated at the bottom of the alignment by angle brackets, where matching $<$ and $>$ denote base-paired columns. Below these angle brackets, the symbol “2” denotes base pairs exhibiting covariation according to the statistically well-founded R-scape method. “1” denotes base pairs exhibiting covariation according to R2R’s simplistic method. “0” denotes base pairs that are not observed to mutate and “?” denotes base pairs that have a significant frequency of non-canonical nucleotides for Watson-Crick or G-U pairs ($> 5\%$). Below these base pair annotation is the consensus sequence: “R” = “A” or “G”, “Y” = “C” or “U”, red nucleotides: nucleotide identity conserved more than 97% of the time, black nucleotides: 90%, gray nucleotides: 75%, red circle (•): nucleotide is present 97% of the time, black circle (•): 90%, gray circle (•): 75%, white circle (○): 50%. All percentages of sequences just described (e.g. 97% conserved) assume that sequences have been weighted by the GSC algorithm implemented by the Infernal software package.

The alignment begins on the next page.

alignment positions 1 · · · 43

```
Ga0180107_1040049/955-919          AUCUUCCCAUCU.UAA.....GGAUGGUGUUUCACCGACAAGU
Ga0307484_100434/157-195           CCUCUCCCAUUU..ACUAA..GAAUGGUUUUCCAUCGGCGAGG
Ga0242646_1006563/621-659          AUCCUCCCACCUG.CA..U.UGGGUGGUUUCUCAUGGACGGAU
Ga0315809_1074825/154-116          CGUCUCCCAGCU..ACAAA..GGCUGGUUUCUCAUGGCCGACA
Ga0222729_1010500/640-600          CGACUCCCAGCU.UAACGAU.GGCUGGUUUCUCAUGGUCGUCA
Ga0307484_101172/1168-1208         CGGCUCCCAGCU.UAACAAU.GGCUGGUUUCUCAUGGACGCCA
Ga0242647_1002406/347-309          CGGCUCCCAGCUA.CUC...UGGCUGGUUUCUCAUGAUCGCCA
Ga0081246_1091038/55-92            UGGCUCCCACCU...GCAA..GGGUGGUUUCUCAUAGUCGCCA
Ga0242647_1000589/1781-1743        UCUCUCCCACCU..ACCAA..GGGUGGUUUUCCAUGGACGAGA
Ga0222729_1000922/327-364          UCUCUCCCACCU...ACAA..GGGUGGUUUUCCAUGGACGAGA
JGI12271J12027_1054298/1098-1058   UCUCUCCCACCU..CGCAAACGGGUGGUUUCUCAUGGACGAGA
Ga0222729_1001724/569-607          CGUCUCCCACCUA.CCC...UGGGUGGUUUCUCAUGGACGACA
Ga0307483_1135034/105-142          AGCCUCCCAACU...GCGA..GGUUGGUUUCUCAUGGACGGCA
Ga0307483_1000720/112-149          UGCCUCCCAACU.ACG..A..GGUUGGUUUCUCAUGGACGGCA
Ga0316034_101609/655-692           GACCUCCCACUU...GCAA..GAGUGGUUUUCCAUGGACGGUC
Ga0184582_120986/467-505           GACCUCCCACUU..ACAAA..GAGUGGUUUUCCAUGGACGGUC
Ga0265743_100264/1856-1819         GAUCUCCCAGUU...ACAA..GACUGGUUUUCCAUGGACGAUC
Ga0265758_100155/1682-1645         GAUCUCCCAGUU...ACAA..GACUGGUUUCCCAUGGACGAUC
Ga0184593_124150/206-166           GCCCUCCCAGUUA.CGCGC.UGACUGGUUUUCCAUAGGCGGGC
Ga0184587_121658/578-538           GCCCUCCCAGUUG.CGCGU.UGACUGGUUUUUCAUAGACGGGC
Ga0184570_111614/962-1002          GCCCUCCCAGUUA.CAUAU.UGACUGGUUUUUCAUAGGCGGGC
Ga0184596_108896/108-148           GCCCUCCCAGUUG.CGCGC.UGACUGGUUUUUCAUAGGCGGGC
Ga0184602_103723/118-78            GCCCUCCCAGUUG.CGCAU.UGACUGGUUUUUCAUAGGCGGGC
Ga0184572_101436/598-558           GCCCUCCCAGUUG.CGCAC.UGACUGGUUUUUCAUAGGCGGGC
Ga0247524_101842/1060-1100         GCCCUCCCAGUUA.CGCAU.UGACUGGUUUUUCAUAGGCGGGC
Ga0184585_110378/50-10             GCCCUCCCAGUUA.CGCUU.UGACUGGUUUUUCAUAGGCGGGC
Ga0184571_113647/73-113            GCCCUCCCAGUUA.CGCGU.UGACUGGUUUUUCAUAGGCGGGC
Ga0316047_100268/1785-1825         GCCCUCCCAGUUA.CGUGU.UGACUGGUUUUCCAUAGGCGGGC
Ga0247526_101949/1557-1517         GCCCUCCCAGUUA.CGUGU.UGACUGGUUUUUCAUAGGCGGGC
Ga0184602_104873/1759-1719         GUCCUCCCAGUUA.CGCAU.UGACUGGUUUCACAGGAUCGGAC
Ga0247547_100059/1080-1040         GUCCUCCCAGUUA.CGCAU.UGACUGGUUUCACAGGUUCGGAC
Ga0247551_103566/144-184           GUCCUCCCAGUUA.CGCAU.UGACUGGUUUUACAGGUUCGGAC
Ga0184574_100854/48-8              GUCCUCCCAGUUA.CGCAA.UGACUGGUUUUACAGGGUCGGAC
Ga0184597_103059/890-930           GUCCUCCCAGUUA.CGCAU.UGACUGGUUUUACAGGGUCGGAC
Ga0265756_100485/51-91             GAUCUCCCAACUG.CAAAU.CGGUUGGUUUCUCAUUGACGAUC

                                   <<<<..<<<<<<<.......>>>>>>>............>>>>
                                   2221..0002201.......1022000............1222
                                   •• CUCCCA•YU--∘ ∘∘∘--GR•UGGUUUYYCA  G CG ••
                                   5′
```

12 HRIMA-9

12.1 Multiple-sequence alignment

For a description of this alignment, see Supplementary Table 2.

Each ribozyme in this alignment is identified by its genomic location in the form SEQID/START-END. SEQID (the sequence accession) is derived from sources such as IMG/M or GenBank. START is the coordinate of the 5′ nucleotide of the hairpin ribozyme, and END corresponds to the 3′ nucleotide. If START>END, then the ribozyme is on the reverse complement strand.

Nucleotides proposed to basepair as part of the consensus structure are shaded in color when they comprise Watson-Crick or G-U pairs. Otherwise they are shaded gray. Conserved stems are also indicated at the bottom of the alignment by angle brackets, where matching < and > denote base-paired columns. Below these angle brackets, the symbol "2" denotes base pairs exhibiting covariation according to the statistically well-founded R-scape method. "1" denotes base pairs exhibiting covariation according to R2R's simplistic method. "0" denotes base pairs that are not observed to mutate and "?" denotes base pairs that have a significant frequency of non-canonical nucleotides for Watson-Crick or G-U pairs (> 5%). Below these base pair annotation is the consensus sequence: "R" = "A" or "G", "Y" = "C" or "U", red nucleotides: nucleotide identity conserved more than 97% of the time, black nucleotides: 90%, gray nucleotides: 75%, red circle (•): nucleotide is present 97% of the time, black circle (•): 90%, gray circle (•): 75%, white circle (∘): 50%. All percentages of sequences just described (e.g. 97% conserved) assume that sequences have been weighted by the GSC algorithm implemented by the Infernal software package.

The alignment begins on the next page.

alignment positions 1 · · · 95

```
Ga0265755_100088/2219-2128   GGUGUUUUCUUGCU.UUGUCCCCGGUCCUUGAAAGGGGAUGCAGG.GG.AAUGAGAUCUCACCUAGGAAUGGGUGGCGGUCAGCUGACUGCCAUC
Ga0265745_1000046/926-1015   GGUGUCUUCUUGUUCUUGUUCCCGGUCC..GAAA..GGAUGCAGG.GAGGAUGAGAUCUCACCUAGGAAUGGGUAGCGGUCAGCCGACUGCUAUC
Ga0265757_100063/721-810     GGUGUUUUCUUGUUCUUGUCUCCGGUCC..GUAA..GGAUGCAGG.AGGGAUGAGAUCUCACCUAGGAACAGGUUGCGGUCAGCUGACUGCAAUC
Ga0265750_1045939/108-19     GGUGUUUUCUUGUUCUUGUCUCCGGCUU..GAAA..GAGUGCAGG.GGGAAUGAGAUCUCACCUAGGAAAAGGUUGCGGUCAGCUGACUGCAAUC
Ga0265745_1003474/738-655    GGGUUUGUCGUGU.CGUGUUCCCGGUC...UUCG...GAUGCAGG.GAGUGUGCGGCUUACCU.AGAGAUGGGCGGGGGG.UUAG.CCUCCCGUC
Ga0265757_100085/737-654     GGGUUUGUCGUGU.UGUGUUCCCGAUC...UUCG...GAUGCAGG.GAGUGUGCGGCUUACCU.AGAGAUGGGCGGGGGG.UUAG.CCUCCCGUC
Ga0265750_1030526/692-608    AGGGUUUGCUGCGUGGAGUCCCCGGUC...UUAG...GACGCAGGAGGAGAGCGGCU.UACCU.AGAGAUGGGCGGGGGG.UUAG.CCUCCCGUC
Ga0310106_100179/2350-2434   GGGCUUUGUCGCGCGGUGUCCUCGGUC...UUCG...GAUGCAGAGGGAUAGCGGCU.UACCU.AGAAGAGGGCGGGGGG.UUAG.CCUCCCGUC
Ga0265755_102448/437-521     GGGCUUUGUCGCGCGGUGUCCUCUGUC...UUCG...GAUGCAGAGGGAUAGCGGCU.UACCU.AGAAGAGGGCGGGGGG.UUAG.CCUCCCGUC
Ga0265757_102423/793-709     AGGUUUUGUCGCGCGGAGUCCUCGGUC...UUUU...GAUGCAGAGGGAUUGCGGCU.UACCU.AGAGAUGGGCGGGGGG.UUAG.CCUCCCGUC

                             <<<<...<<<<<<<<....<<<<.<<<<<<....>>>>>>...>>.>>>>>>>>>>...>>>>........<<<<<<<<<<....>>>>>>>>>>
                             101....1.111.......2120.111.00....00.111...02.12...111.1...101........0012010010....0100102100
                              G••U  •  ••• ◦• GUY  CG    ---••• ---   GCAG - R • •• G• ◦U••CY-AGRR   GGY G•GG•-••••-•CU•C RUC
                             5′
```

13 HRIMA-10

13.1 Multiple-sequence alignment

For a description of this alignment, see Supplementary Table 2.

Each ribozyme in this alignment is identified by its genomic location in the form SEQID/START-END. SEQID (the sequence accession) is derived from sources such as IMG/M or GenBank. START is the coordinate of the 5′ nucleotide of the hairpin ribozyme, and END corresponds to the 3′ nucleotide. If START>END, then the ribozyme is on the reverse complement strand.

Nucleotides proposed to basepair as part of the consensus structure are shaded in color when they comprise Watson-Crick or G-U pairs. Otherwise they are shaded gray. Conserved stems are also indicated at the bottom of the alignment by angle brackets, where matching < and > denote base-paired columns. Below these angle brackets, the symbol "2" denotes base pairs exhibiting covariation according to the statistically well-founded R-scape method. "1" denotes base pairs exhibiting covariation according to R2R's simplistic method. "0" denotes base pairs that are not observed to mutate and "?" denotes base pairs that have a significant frequency of non-canonical nucleotides for Watson-Crick or G-U pairs (> 5%). Below these base pair annotation is the consensus sequence: "R" = "A" or "G", "Y" = "C" or "U", red nucleotides: nucleotide identity conserved more than 97% of the time, black nucleotides: 90%, gray nucleotides: 75%, red circle (•): nucleotide is present 97% of the time, black circle (•): 90%, gray circle (•): 75%, white circle (◦): 50%. All percentages of sequences just described (e.g. 97% conserved) assume that sequences have been weighted by the GSC algorithm implemented by the Infernal software package.

The alignment begins on the next page.

alignment positions 1 · · · 87

```
Ga0242641_1002366/1052-1123    CAGUG.UCGUCCCGCUUU......AGAC.GCUCUGUUU.CUCUCAGGUUCUGGACUUACAUACC.GGUGCCG..AUG..CGGCA.UC
Ga0242646_1001007/114-185      CCCCUGGUUUCCCUUU.........GAAUGCCA.GGGU.CGUAAUGUCACUGGACCGAGUUU.C.UGUGCGGAAUUC..CCGCCUUC
Ga0242652_1002960/985-916      CGAGCCU....ACCAGUAACAGAAA.....AGG.CUCUGCUUUCGGUU.CUGGACUGAGUUGAC.GGGACUG..UUG..CAGUC.UC
Ga0242646_1000880/1297-1228    AUCAGGAUUUCCCAAU.........GAAUGUUC.UGAUGUCUCCGGUU.CUGGACUGAGUUGUG.GGGGCUG..CCG..CAGCC.UC
Ga0247553_121301/414-345       CGACCUGUUUCCCAAU.........GAAUUCAG.GUUG.ACUAUCGUUUCUGGACUGAUAUGUG.GCCACUG..UUG..CAGUG.GC
Ga0265753_1014276/341-411      CUGUCCUG...ACUAUUUGCGAA......CGGG.GCAGUCCUCUUGUGUCUGGACUGACAUGAC.GGUGCUG..UUG..CAGCA.UC
Ga0184589_110648/454-524       CUAUUCCUUUCCCUUAU........GAAUGGGA.GUAG.ACCUCAGUUUCUGGACUGAUACGAC.GGCACUG..UUG..CAGUG.UC
Ga0184586_124000/210-279       CUGCUCCUUUCCCUAU.........GAAUGGGA.GUAG.ACCUCAGUUUCUGGACUGAUACGAC.GGCACUG..UUG..CAGUG.UC
Ga0184601_103200/159-90        CUGCUCCUUUCCCUAU.........GAAUGGGA.GUAG.ACCUCAGUUUCCGGACUGAUACGAC.GGCACUG..UUG..CAGUG.UC
Ga0247527_104639/551-480       CUGCCCUUUUCCCAUU.........GAAUAAGGAGCAGACCUCAAGUGUCUGGACUGACAUGAC.GGCGCUG..UUG..CAGCG.UC
Ga0184602_128425/1345-1274     CUGCCCUUUUCCCAUU.........GAAUAAGGAGCAGACCUCAAGUGUCUGGACUGACAUGAC.GGCGCUG..AUG..CAGCG.UC
Ga0265758_100254/942-872       CUGUUCCUUUCCCUAU.........GAAUGGGA.GCAGACCUCUUGUGUCUGGACUGAUAUGAC.GGCACUG..UUG..CAGUG.UC
Ga0184588_104808/764-834       CUGC.CCUUUCCCAUUU........GAAUAGGA.GCAGACCUCUUGUGUCUGGACUGACAUGAC.GGCACUG..UUG..CAGUG.UC
Ga0316052_100597/74-129        UCG........UUUUC...................CGACCAUCCAACGUCUGGACUCAUGUAUC.GCGAUCU..GAGAGAGAUC.GC
Ga0184596_111007/1129-1076     UGG........UUUUC...................CGACCAUCCAACGUCUGGACUCAUGUAUC.GCGGCUG..UCU..CAGUC.GC
Ga0247550_104012/240-170       CGUCGUUUUUCCCAAAU........GACUAGAC.GACG.CCUAUCGAAUCUGGACUCAUGUAUC.GCGGCUG..CCU..CAGUC.GC
Ga0184601_104622/961-891       CGUCGUUUUUCCCAAAU........GACUAGAC.GGCG.CCUAUCGAAUCUGGACUCAUGUAUC.GCGGCUG..UCG..CAGCC.GC
Ga0184600_100406/690-620       CGUCGUUUUUCCCAAAU........GACUAGAC.GACG.CCUAAUGAAUCUGGACUCAUGUAUC.GCGGCUG..UCU..CAGUC.GC
Ga0247524_104389/615-545       CGUCGUUUUUCCCAAAU........GACUAGAC.GACG.CCUAAUGAAUCUGGACUCAUGUAUC.GCGGCUG..UCU..CAGCC.GC
Ga0184597_106380/237-307       CGUCGUUUUUCCCAAAU........GACUAGAC.GACG.CCUAAUGAAUCUGGACUCAUGUAUC.GCGGCUG..CCU..CAGCC.GC
Ga0247529_103231/893-823       CGUCGUUUUUCCCAAAU........GACUAGAC.GGCG.CCUAAUGGAUCUGGACUCAUGUAUC.GCGGCUG..UCU..CAGUC.GC
Ga0184597_103142/318-248       CGUCGUUUUUCCCAAAU........GACUAGAC.GGCG.CCUAAUGAAUCUGGACUCAUGUAUC.GCGGCUG..UCU..CAGCC.GC
Ga0247519_107918/901-971       CGUCGUUUUUCCCAAAU........GACUAGAC.GGCG.CCUAAUGAAUCUGGACUCAUGUAUC.GCGGCUG..UCU..CAGUC.GC
Ga0184590_115609/423-493       CGUCAUUUUUCCCAAAU........GAGUUGAU.GGCG.CCUAACGAGUCUGGACUCAUGUAUC.GCGGCUU..ACG..CAGUC.GC
Ga0247551_100854/544-472       CUGCAUUUUUCCCAUUC........GAAUAGAU.GCAG.CCCUUCGAAUCUGGACUCAUGUAUC.GCGAUCU..GAGAGAGAUC.GC
Ga0184603_115994/649-720       CUGCAUCUUUCCCAAU.........GAAUAGAU.GUAG.CCCGUCGAAUCUGGACUCAUGUAUC.GCGAUCU..GAGAGAGAUC.GC
Ga0184584_129277/160-231       CUGCAUUUUUCCCAAU.........GAACAGAU.GCAG.CCCGUCGAGUCUGGACUCAUGUAUC.GCGAUCU..GAGAGAGAUC.GC
Ga0247547_100082/1185-1256     CUGCAUUUUUCCCAAU.........GAACAGAU.GCAG.CCCGUCGAAUCUGGACUCAUGUAUC.GCGAUCU..GAGAGAGAUC.GC
Ga0316051_1010660/179-251      CUGCAUUUUUCCCAACU........GAACAGAU.GCAG.CCCUUCGAAUCUGGACUCAUGUAUC.GCGAUCU..GAGAGAGAUC.GC
Ga0184603_106387/123-194       CUACAUCUUUCCCAAU.........GAAUAGAU.GUAG.CCCAUCGAGUCUGGACUCAUGUAUC.GCGAUCU..GAGAGAGAUC.GC
Ga0184597_124012/148-76        CUACAUCUUUCCCAAAU........GAAUAGAU.GUAG.CCCAUCGAGUCUGGACUCAUGUAUC.GCGAUCU..GAGAGAGAUC.GC
Ga0247546_107817/147-75        CUACAUCUUUCCCAUUC........GAAUAGGU.GUAG.CCCAUCGAGUCUGGACUCAUGUAUC.GCGAUCU..GAGAGAGAUC.GC
Ga0184600_108003/252-324       CUACAUCUUUCCCAAU.........GAAUAAGGUGUAG.CCCAUCGAGUCUGGACUCAUGUAUC.GCGAUCU..GAGAGAGAUC.GC

                               <<<<<<<<<<<..............>>>.>>>>.>>>>..........................<<<<<<<.......>>>>>.>>
                               .2212....00..............00.....2.122...........................0221221.......12212.20
```

14 HRIMA-11

14.1 Multiple-sequence alignment

For a description of this alignment, see Supplementary Table 2.

Each ribozyme in this alignment is identified by its genomic location in the form SEQID/START-END. SEQID (the sequence accession) is derived from sources such as IMG/M or GenBank. START is the coordinate of the 5′ nucleotide of the hairpin ribozyme, and END corresponds to the 3′ nucleotide. If START>END, then the ribozyme is on the reverse complement strand.

Nucleotides proposed to basepair as part of the consensus structure are shaded in color when they comprise Watson-Crick or G-U pairs. Otherwise they are shaded gray. Conserved stems are also indicated at the bottom of the alignment by angle brackets, where matching < and > denote base-paired columns. Below these angle brackets, the symbol "2" denotes base pairs exhibiting covariation according to the statistically well-founded R-scape method. "1" denotes base pairs exhibiting covariation according to R2R's simplistic method. "0" denotes base pairs that are not observed to mutate and "?" denotes base pairs that have a significant frequency of non-canonical nucleotides for Watson-Crick or G-U pairs ($> 5\%$). Below these base pair annotation is the consensus sequence: "R" = "A" or "G", "Y" = "C" or "U", red nucleotides: nucleotide identity conserved more than 97% of the time, black nucleotides: 90%, gray nucleotides: 75%, red circle (•): nucleotide is present 97% of the time, black circle (•): 90%, gray circle (•): 75%, white circle (◦): 50%. All percentages of sequences just described (e.g. 97% conserved) assume that sequences have been weighted by the GSC algorithm implemented by the Infernal software package.

The alignment begins on the next page.

alignment positions 1 · · · 86

```
Ga0184570__115412/234-152   GU.UGGAGAGCGCAGAUAUGUACGUCUGGAGUUUCCUCCCCUGGUGAUAUGCCAGGAUAGACCAUCU.GCGCCCCGGGA.UCCAAC
Ga0184582__100657/349-267   GU.UGGAAGGCGCAGAUAUGUACGUCUGGAGUUUCCUCCCCCGGUGAUAUGCCGGGAUAGACCAUCU.GCGCCUCGGGA.UCCAAC
Ga0265757__100423/743-824   CA.UGGAGAUAACUAGCUCUAC.GUCUGGAGUUACCUCCCCGAGUUGUAUGCUCGGAUAGACCGCUA.GUUAUCCUGGG.UCCAUG
Ga0184582__110195/163-244   CA.UGGAGAUGACUAGCUAUAC.GUCUGGAGUUACCUCCCCGAGUUGUAUGCUCGGAUAGACCGCUA.GUCAUCCUGGG.UCCAUG
Ga0316037__173552/225-144   CA.UGGAAGCGACUAGCUCUAC.GUCUGGAGUUACCUCCCCGAGUGAUAUGCUCGGAUAGACCGCUA.GUCGCUCUGGG.UCCAUG
Ga0247514__107497/575-656   CA.UGGAAGCGACUAGCUAUAC.GUCUGGAGUUACCUCCCCGAGUGAUAUGCUCGGAUAGACCGCUA.GUCGCUCUGGG.UCCAUG
Ga0184597__118518/152-233   CA.UGGAAGCGACUAGCUAUAC.GUCUGGAGUUACCUCCCCGAGUGAUAUGCUCGGAUAGACCGCUA.GUCAUCCUGGG.UCCAUG
Ga0184583__122578/285-203   GA.GACAUGUCGACAAGGUUCACGUGCGGAGUUUCCUCCCCAGGUUGUAUGCCUGGUAGCACCCUUG.UUGACGUGGUA.UGUCAC
Ga0247514__114593/670-587   GG.UUGAAAUGUACUCUACCAUGGUGUGGAGUUUCCUCCCCAGGUGGUAUGCCUGGUUGCACAAGGG.UACAUUUGGUUAUCAACC
Ga0184582__132568/349-266   GG.UUGAAAUGUACCCUACCAUGGUGUGGAGUUUCCUCCCCAGGUGGUAUGCCUGGUUGCACAAGGG.UACAUUUGGUUAUCAACC
Ga0265758__114319/310-227   GG.UUGAAAUGUACCCUACCAUGGUGUGGAGUUUCCUCCCCAGGUGGUAUGCCUGGUUGCACAAAGG.UACAUUUGGUUAUCAACC
Ga0184595__111629/288-205   GG.UUGAAAUGUACCCUACCAUGGUGCGGAGUUUCCUCCCCAGGUGGAAUGCCUGGCUGCACAAAGG.UACAUUUGGUUAUCAACC
Ga0310118__102883/498-582   GUACUGUAAACAACACGAUCACAGUCUGGAGUUUCCUCCCCUGGUUGUAUGCCAAGAUAGACCCGUG.UUGUUUAGGUUAACAGAC
Ga0265750__1142740/126-210  GUACUGUAAACAACACGAUCACAGUCUGGAGUUUCCUCCCUUGGUUAUAUGCCAAGAUAGACCCGUG.UUGUUUAGGUUAACAGAC
Ga0265745__1099723/231-147  GUACUGUAAACAACACGAUCACAGUCUGGAGUUUCCUCCCUUGGUUGUAUGCCAAGAUAGACCCGUG.UUGUUUAGGUUAACAGAC
Ga0184588__145412/985-901   GUACUGUAAACGACACGAUCACAGUCUGGAGUUUCCUCCCUUGGUUGUAUACCAAGAUAGACCCGUG.UUGUUUAGGUUAACAGAC

                            <<.<<<<<<<<<<<<<<......<<<<<<<<....>>>><<<<<<......>>>>>>..>>>>.>>>>.>>>>>>......>>>>>>
                            1..211121221212.2......00210000....000001210.......01210..1200.2.21.212212......1112.1
                         5′ •–  •    R•• •   ••    •••GU•YGGAGUU CCUCCC • GU•RUAUGC • G• R•AC ••  –• ••Y   G••–  • •
```

15 HRIMA-12

15.1 Multiple-sequence alignment

For a description of this alignment, see Supplementary Table 2.

Each ribozyme in this alignment is identified by its genomic location in the form SEQID/START-END. SEQID (the sequence accession) is derived from sources such as IMG/M or GenBank. START is the coordinate of the 5′ nucleotide of the hairpin ribozyme, and END corresponds to the 3′ nucleotide. If START>END, then the ribozyme is on the reverse complement strand.

Nucleotides proposed to basepair as part of the consensus structure are shaded in color when they comprise Watson-Crick or G-U pairs. Otherwise they are shaded gray. Conserved stems are also indicated at the bottom of the alignment by angle brackets, where matching $<$ and $>$ denote base-paired columns. Below these angle brackets, the symbol "2" denotes base pairs exhibiting covariation according to the statistically well-founded R-scape method. "1" denotes base pairs exhibiting covariation according to R2R's simplistic method. "0" denotes base pairs that are not observed to mutate and "?" denotes base pairs that have a significant frequency of non-canonical nucleotides for Watson-Crick or G-U pairs ($> 5\%$). Below these base pair annotation is the consensus sequence: "R" = "A" or "G", "Y" = "C" or "U", red nucleotides: nucleotide identity conserved more than 97% of the time, black nucleotides: 90%, gray nucleotides: 75%, red circle (•): nucleotide is present 97% of the time, black circle (•): 90%, gray circle (•): 75%, white circle (◦): 50%. All percentages of sequences just described (e.g. 97% conserved) assume that sequences have been weighted by the GSC algorithm implemented by the Infernal software package.

The alignment begins on the next page.

alignment positions 1 · · · 109

```
Ga0247526_101949/44-154      AAUGUGUACUUUGAUGUUGACGGGAGAUGCGAGCUCAGUUCAUU...CUG.ACCGUAUCUUGGGGAGAGUCCAUCUACUUAC..GUCGAAUGUCGCGCUUGACCUGGAG
Ga0316046_102147/791-901     AAUGUGUACUUUGAUGUUGACGGGGGAUGCGAGCUCAGUUCAUU...CUG.ACCGUAUCCUGGGGAGAGUCCAUCUACUUAC..GUCGAAUGUCGCGCUUGACCUGGAG
Ga0247537_100205/932-1042    AAUGUGUACUUUGAUGUUGACGGGGGAUGCGAGCUCAGUUCAUU...CUG.ACCGUAUCUUGGGGAGAGUCCAUCUACUUAC..GUCGAAUGUCGCGCUUGACCUGGAG
Ga0247524_101842/775-665     AAUGUGUACUUUGAUGUCGACGGAGGAUGCGAGCUCAGUUCAUU...CUG.ACCGUAUCUUAGGGAGAGUCCAUCUACUUAC..GUCGAAUGUCGCGUUCGACCUGGAG
Ga0184597_124069/84-194      AAUGUGUGCUUUGAUGUCGACGGAAGAUGCGAGCUCAGUUCAUU...CUG.ACCGUAUCUUAGGGAGAGUCCAUCUACUUAC..GUCGAAUGUCGCGUUCGACCUGGAG
Ga0247529_101980/1065-1175   AAUGUGUGCUUUGAUGUCGACGGAGGAUGCGAGCUCAGUUCAUU...CUG.ACCGUAUCUUAGGGAGAGUCCAUCUACUUAC..GUCGAAUGUCGCGUUCGACCUGGAG
Ga0184570_111614/676-566     AAUGUGUACUUUGAUGUCGACGGAGGAUGCGAGCUCAGUUCAUU...CUG.ACCGUAUCUUAGGGAGAGUCCAUCUACUUAC..GUCGAAUGUCGCGUUUGACCUGGAG
Ga0184572_101436/882-992     AAUGUGUGCUUUGAUGUCGACGGAGGAUGCGAGCUCAGUUCAUU...CUG.ACCGUAUCUUAGGGAGAGUCCAUCUACUUAC..GUCGAAUGUCGUGUUUGACCUGGAG
Ga0184573_109710/230-340     AAUGUGUGCUUUGAUGUCGACGGAAGAUGCGAGCUCAGUUCAUU...CUG.ACCGUAUCUUAGGGAGAGUCCAUCUACUUAC..GUCGAAUGUCGCGUUUGACCUGGAG
Ga0184602_102112/249-359     AAUGUGUGCUUUGAUGUCGACGGAGGAUGCGAGCUCAGUUCAUU...CUG.ACCGUAUCUUAGGGAGAGUCCAUCUACUUAC..GUCGAAUGUCGCGUUUGACCUGGAG
Ga0247512_126637/248-140     AGGGUCGUUAAAGAUAAUCGUCGGAGGUGAGAGCUCAGUGAAAACUGCUGUA.CUCAUCUUCCUCUAGUUCAUUGAACU...AUGUUGU..AUAAC..ACGACAUAAAG
Ga0247525_119251/328-220     AGGGUCGUUAAAGAUAAUCGUCGGAGGUGAGAGCUCAGUUAAUUCUGCUGUA.CUCAUCUUCCUCUAGUUUAUUAAUAAACUACGUUGU..AUAAC..ACGAC...UUA
Ga0184595_131794/77-185      AGGGUCGUUAAAGAUAAUCGUCGGAGGUGAGAGCUCAGUUAAUUCUGCUGUA.CUCAUCUUCCUCUAGUUAAUUCAUUAACUACGUUGU..AUAAC..ACGAC...UUA
Ga0184594_117256/385-277     AGGGUCGUUAAAGAUAAUCGUCGGAGGUGGGAGCUCAGUUAAUUCUGCUGUA.CUCAUCUUCCUCUAGUUAAUUAAUUAACUACGUUGU..AUAAC..ACGAC...UUA
Ga0184595_104471/44-151      AGGGUCGUUAAAGAUAAUCGUCGGAGGUGAGAGCUCAGUUAAUUCUGCUGUA.CUCAUCUUCCUCUAGUUAA.UAAUUAACUACGUUGU..AUAAC..ACGAC...UUA
Ga0184600_130729/43-151      AGGGUCGUUAAAGAUAAUCGUCGGAGGUGAGAGCUCAGUUAAUUCUGCUGUA.CUCAUCUUCCUCUAGUUAAUUUAUUAACUACGUUGU..AUAAC..ACGAC...UUA
Ga0184568_112547/160-268     AGGGUCGUUAAAGAUAAUCGUCGGAGGUGAGAGCUCAGUUAAUUCUGCUGUA.CUCAUCUUCCUCUAGUUAAUUAAUUAACUACGUUGU..AUAAC..ACGAC...UUA
Ga0184571_109616/346-239     AGGGUCGUUAAAGAUAAUCGUCGGAGGUGAGAGCUCAGUUAAUUCUGCUGUA.CUCAUCUUCCUCUAGUUAAUU.AUUAACUACGUUGU..AUAAC..ACGAC...UUA
                             ..<<<..<<......>>..>>>.<<<<<<<<...<<<<.........>>>.>.>>>>>>>>.......................<<<<<<<.....>>>>>>>......
                             ..1....11......11....1..10.0.10...0000.........000.0.01.0.01........................00..1.0.....0.1..00......
                             AR•GU•••Y•••GAUR•Y•RY•GRRGRUG•GAGCUCAGU •A   ○○○CUG○A-C•YAUCUU•••••RR•U ••U••• ••• ○○GUYG•--RU•RC--•YGAC○○○••R
                           5′
```

16 HRIMA-13

16.1 Multiple-sequence alignment

For a description of this alignment, see Supplementary Table 2.

Each ribozyme in this alignment is identified by its genomic location in the form SEQID/START-END. SEQID (the sequence accession) is derived from sources such as IMG/M or GenBank. START is the coordinate of the 5′ nucleotide of the hairpin ribozyme, and END corresponds to the 3′ nucleotide. If START>END, then the ribozyme is on the reverse complement strand.

Nucleotides proposed to basepair as part of the consensus structure are shaded in color when they comprise Watson-Crick or G-U pairs. Otherwise they are shaded gray. Conserved stems are also indicated at the bottom of the alignment by angle brackets, where matching $<$ and $>$ denote base-paired columns. Below these angle brackets, the symbol "2" denotes base pairs exhibiting covariation according to the statistically well-founded R-scape method. "1" denotes base pairs exhibiting covariation according to R2R's simplistic method. "0" denotes base pairs that are not observed to mutate and "?" denotes base pairs that have a significant frequency of non-canonical nucleotides for Watson-Crick or G-U pairs ($> 5\%$). Below these base pair annotation is the consensus sequence: "R" = "A" or "G", "Y" = "C" or "U", red nucleotides: nucleotide identity conserved more than 97% of the time, black nucleotides: 90%, gray nucleotides: 75%, red circle (•): nucleotide is present 97% of the time, black circle (•): 90%, gray circle (•): 75%, white circle (○): 50%. All percentages of sequences just described (e.g. 97% conserved) assume that sequences have been weighted by the GSC algorithm implemented by the Infernal software package.

The alignment begins on the next page.

alignment positions 1 · · · 116

```
Ga0265756__100592/233-318     GUGACUAGCAAUCACUGCGCCAUCUUCGGGUGGACAACACACGGAUAU...........A...................CCGAGCGUACCCCCAGGAAGCGAAGCUAUGCUCUGGG
Ga0247543__100289/621-524     GCAUCUAGCAAAUGC.CCCUCUACCUCGGUAGAA.AACACACGG..........UCGCCUAAC...GGCA...AUAAUUCCGCACGUACUUCUAGGAAGCGAAGCUAUGCUCUAGA
Ga0247551__100191/1076-1173   GCAUCUAGCAAAUGC.CCCUCUACCUCGGUAGAA.AACACACGG..........UCGCCUGAC...GGCA...AUAAUUCCGCACGUACUUCUAGGAAGCGAAGCUAUGCUCUAGA
Ga0184599__115806/205-309     GCGAGUAGCGACCGC.CCCUCUACUUCGGUAGAA.AACACACGGUUCCUAAAUAC.....CUAUGG...GUAUAAA.GACCGCACGUACUUCUAGGAAGCGAAGCUAGGCUCUAGA
Ga0184602__101782/1237-1133   GCGAGUAGCGACCGC.CCCUCUACUUCGGUAGAA.AACACACGGUUCCUAAAUAC...CAAUG...G..GUAUAAA.GACCGCACGUACUUCUAGGAAGCGAAGCUAGGCUCUAGA
Ga0247536__100773/469-362     GCGAUUAGCAAUCGC.CCCUCUACCUAGGUAGAA.AACACACGGAUGU...CAG.CGGGACUAAAGCCCCCUGAAAA..CCGCUCGUACUUCUAGGAAGCGAAGCUAUGCUCUAGA
Ga0184573__112336/491-597     GCGAUUAGCAACCGC.CCCUCUACCUCGGUAGAA.AACACACGGAUG....UUGGCGGGACUUAAGCCCCCAAAUA...CCGCACGUACUUCUAGGAAGCGAAGCUAGGCUCUAGA
Ga0247530__109035/657-763     GCGAUUAGCAACCGC.CCCUCCACUUCGGUGGAA.AACACACGGAUG....UUGGCGGGACUAAAGCCCACAAUUA...CCGCGCGUACUUCUAGGAAGCGAAGCUAGGCUCUAGA
Ga0247525__113793/5-111       GCGAUUAGCAACCGC.CCCUCCACUUCGGUGGAA.AACACACGGAUG....UUGGCGGGACUAAAGCCCCCAAUUA...CCGCACGUACUUCUAGGAAGCGAAGCUAGGCUCUAGA
Ga0247538__100338/52-158      GCGAUUAGCAACCGC.CCCUCCACUUCGGUGGAA.AACACACGGAUG....UUGGCGGGACUAAAGCCCCCAAUUA...CCGCGCGUACUUCUAGGAAGCGAAGCUAGGCUCUAGA
Ga0184602__101499/638-532     GCGAUUAGCAACCGC.CCCUCCACUUCGGUGGAA.AACACACGGAUG....UCAGCGGGACUUAAGCCCCUGAAAA...CCGCUCGUACUUCUAGGAAGCGAAGCUAGGCUCUAGA
Ga0265759__100473/651-545     GCGAUUAGCAACCGC.CCCUCUACUUCGGUAGAA.AACACACGGAUG....UCAGCGGGACUAAAGCCCCUGAAAA...CCGCUCGUACCUCUAGGAAGCGAAGCUAGGCUCUAGA
Ga0247512__108804/659-765     GCGAUUAGCAACCGC.CCCUCUACUUCGGUAGAA.AACACACGGAUG....UCAGAGGGAGUAAAGCCCCUGAAAA...CCGCGCGUACCUCUAGGAAGCGAAGCUAGGCUCUAGA

                              <<<.........>>>....<<<<<....>>>>>........<<<.......<<<<.<<<.......>>>>>>>......>>>........<<<<<..<<<........>>>>>>>>
                              011.........110....10210....01201........000.......221..011.......110.122......000........10100..000........00000101
                              G    UAGC A   C- C  C  CYU GG  G A-AACACACGG• ••---ooooooooooooooooooooooo• ---CCG  CGUACY C AGGAAGCGAAGCUA•GCUCU G
                              5′
```

17 HRIMA-14

17.1 Multiple-sequence alignment

For a description of this alignment, see Supplementary Table 2.

Each ribozyme in this alignment is identified by its genomic location in the form SEQID/START-END. SEQID (the sequence accession) is derived from sources such as IMG/M or GenBank. START is the coordinate of the 5′ nucleotide of the hairpin ribozyme, and END corresponds to the 3′ nucleotide. If START>END, then the ribozyme is on the reverse complement strand.

Nucleotides proposed to basepair as part of the consensus structure are shaded in color when they comprise Watson-Crick or G-U pairs. Otherwise they are shaded gray. Conserved stems are also indicated at the bottom of the alignment by angle brackets, where matching < and > denote base-paired columns. Below these angle brackets, the symbol "2" denotes base pairs exhibiting covariation according to the statistically well-founded R-scape method. "1" denotes base pairs exhibiting covariation according to R2R's simplistic method. "0" denotes base pairs that are not observed to mutate and "?" denotes base pairs that have a significant frequency of non-canonical nucleotides for Watson-Crick or G-U pairs (> 5%). Below these base pair annotation is the consensus sequence: "R" = "A" or "G", "Y" = "C" or "U", red nucleotides: nucleotide identity conserved more than 97% of the time, black nucleotides: 90%, gray nucleotides: 75%, red circle (•): nucleotide is present 97% of the time, black circle (•): 90%, gray circle (•): 75%, white circle (o): 50%. All percentages of sequences just described (e.g. 97% conserved) assume that sequences have been weighted by the GSC algorithm implemented by the Infernal software package.

The alignment begins on the next page.

alignment positions 1 · · · 49

```
Ga0265745_1003474/129-340     AUCCGCAAGUCCCCUCUUCGUUAACCCGAUUCGACCGACGCCGAACAAC
Ga0310106_100179/2926-2750    ....................................GACGCCGAACAAC
Ga0310115_101079/245-457      AUCCUCAAAUCCCCUCUUCAAUAACCCGAUUCGACCGACGCCGAACAAC
Ga0265755_100106/171-382      AUCCUCAAAUCCCCUCUUCAAUAACCCGAUUCGACCGACGCCGAACAAC
Ga0310106_103451/1-176        ....................................GACGCCGAACAAC
Ga0265755_103597/828-616      AUCUUCAAGUCCCCUCUUCGCUAACCCGUUUUGACCGACGCCGAACAAC
Ga0310106_100179/223-11       AUCCUCAAGUCCCCUCUUCGUAAACCCGAUUCGACCGACGCCGAACAAC
Ga0265750_1030526/79-291      AUCCUCAAGUCCCCUCUUCGUAAACCCGAUUCGACCGACGCCGAACAAC
Ga0265757_102423/182-394      AUCCUCAAGUCCCCUCUUCGCUAACCCGUUUUGACCGACGCCGAACAAC
Ga0265757_100085/127-339      AUCCGCAAGUCCCCUCUUCGUUAACCCGAUUCGACCGACGCCGAACAAC
Ga0265740_1197481/141-312     GGAGAGGAACUCAUGCCCUUCUUCGUUACAGAAUUCGACAAACACCGAA
Ga0265755_101715/840-628      GGAGAGGAACUCAUGCCCUUCUUCGUUACAGAAUUCGACAAACGCCGAA
Ga0265745_1111090/67-279      GGAGAGGAACUCAUGCCCUUCUUCGUUACAGAAUUCGACAAACACCGAA
Ga0265745_1000046/1508-1297   GGAGGAAAACUUAUGCCCUUCUUCGUUACAGAAUUAGACGAACACCGAC
Ga0265757_100063/1301-1090    GGAGAACCUCUCAUGCCCUUCUUCGCAAACAAUUUGGACGAACACCGAC
Ga0310115_100964/2708-2893    AGGAGAACAGAUCAUGCCCUUCUUCGCAACGAAUUCGACGAACACCGAC
Ga0265745_1034509/411-200     AGGAGAACAGAUCAUGCCCUUCUUCGCAACGAAUUCGACGAACACCGAC

                              .................................................
                              .................................................
                              R●●●●● ●   Y● ● YYY●  ●●●  R●●●● ●Y●GAC ●●●A●CRA
```

alignment positions 50 · · · 209

```
Ga0265745_1003474/129-340     CUGCAA.GAGGCCCUGGU.GAAUCAG.ACCAGGUGCAGGCCUCAGCACAUCUGC.CUUCA.GUGGUAUCCCCUGGAACUGGUACUUGCAC.UACCAGAACCUCAGGAUU..GUGCU.UUGCAUGGUCAAGAAACACGGCUUGAGACGAUGAAUCCCAAGG
Ga0310106_100179/2926-2750    CUGCAA.GAGGCCCUGGU.GAAUCAA.ACCAGGUGCAGGCCUCAGCACAUCUGC.CUUCA.GUGGUAUCCCCUGGAACUGGUACUUGCAC.UACCAGAACCUCAGGAUU..GUGCUUUUGCAUGGUCAAGGAACAUCCCUUGAGACGAUGAAGCCCAAGG
Ga0310115_101079/245-457      CUGCAA.GAGGCCCUGGU.GAAUCAA.ACCAGGUGCAGGCCUCAGCACAUCUGC.CUUCA.GUGGUAUCCCCUGGAACUGGUACUUGCAC.UACCAGAACCUCAGGAUU..GUGCUUUUGCAUGGUCAAGGAACAUCCCUUGAGACGAUGAAGCCCAAGG
Ga0265755_100106/171-382      CUGCAA.GAGGCCCUGGU.GAAUCAA.ACCAGGUGCAGGCCUCAGCACAUCUGC.CUUCA.GUGGUAUCCCCUGGAACUGGUACUUGCAC.UACCAGAACCUCAGGAUU..GUGCU.UUGCAUGGUCAAGAAACACGGCUUGAGACGAUGAAUCCCAAGG
Ga0310106_103451/1-176        CUGCAA.GAGGCCCUGGU.GAAUCAA.ACCAGGUGCAGGCCUCAGCACAUCUGC.CUUCA.GUGGUAUCCCCUGGAACUGGUACUUGCAC.UACCAGAACCUCAGGAUU..GUGCU.UUGCAUGGUCAAGAAACACGGCUUGAGACGAUGAAUCCCAAGG
Ga0265755_103597/828-616      CUGCAA.GAGGCCCUGGU.GAAUCAA.ACCAGGUGCAGGCCUCAGCACAUCAGC.CUUCA.GUGGUAUCCCCUGGAACUGGUACUUGCAC.UACCAGAACCUUAGGAUU..GUGCUUUUGCAUGGUCAAGGAACAUCCCUUGAGUCGAUGAAGCCCAAGG
Ga0310106_100179/223-11       CUGCAA.GAGGCCCUGGU.GAAUCAA.ACCAGGUGCAGGCCUCAGCACAUCAGC.CUUCA.GUGGUAUCCCCUGGAACUGGUACUUGCAC.UACCAGAACCUUAGGAUU..GUGCUUUUGCAUGGUCAAGGAACAUCCCUUGAGUCGAUGAAGCCCAAGG
Ga0265750_1030526/79-291      CUGCAA.GAGGCCCUGGU.GAAUCAA.ACCAGGUGCAGGCCUCAGCACAUCAGC.CUUCA.GUGGUAUCCCCUGGAACUGGUACUUGCAC.UACCAGAACCUUAGGAUU..GUGCUUUUGCAUGGUCAAGGAACAUCCCUAGAGACGAUGAAGCCCAAGG
Ga0265757_102423/182-394      CUGCAA.GAGGCCCUGGU.GAAUCAA.ACCAGGUGCAGGCCUCAGCACAUCAGC.CUUCA.GUGGUAUCCCCUGGAACUGGUACUUGCAC.UACCAGAACCUUAGGAUU..GUGCUUUUGCAUGGUCAAGGAACAUCCCUUGAGUCGAUGAAGCCCAAGG
Ga0265757_100085/127-339      CUGCAA.GAGGCCCUGGU.GAAUCAA.ACCAGGUGCAGGCCUCAGCACAUCUGC.CUUCA.GUGGUAUCCCCUGGAACUGGUACUUGCAC.UACCAGAACCUUAGGAUU..GUGCUUUUGCAUGGUCAAGAAACACGGCUUGAGACGAUGAAUCCCAAGG
Ga0265740_1197481/141-312     AAGGCUUUUGGGCCCGGAU.GACCA.AUCCGGAUGCAGCCCUCUGCGUGCCG.UC.UCU.GACGGUAAGGCUGUGGUCGGUG..CUGCACA.CACCUUCCACUACCUGUUAGCGCAUAGCUAUCCUUUGAA............................
Ga0265755_101715/840-628      AAGGCUUUUGGGCCCGGAU.GACCA.AUCCGGAUGCAGCCCUCUGCGUGCCG.UC.UCU.GACGGUAAGGCUGUGGUCGGUG..CUGCACA.CACCUUCCACUACCUGUUAGCGCAUAGCUAUCCUUUGAAACAACCCCUGAGACGAUGAAGAGCGUGGU
Ga0265745_1111090/67-279      AAGGCUUUUGGGCCCGGAU.GACCA.AUCCGGAUGCAGCCCUCUGCGUGCCG.UC.UCU.GACGGUAAGGCUGUGGUCGGUG..CUGCACA.CACCUUCCACUACCUGUUAGCGCAUAGCUAUCCUUUGAAACAACCCCUGAGACGAUGAAGAGCGUGGU
Ga0265745_1000046/1508-1297   AAAGCUUUGGGGCCCGGAU.GAUCA.AUCCGGUUGCAGCCCUCUGCGUGCCG.UC.UCU.GACGGUAAGGCUGUGGUUGGUGU..UGCA..GCACCUUCCACUACCUGUUAGCGCAUAGCUAUCCUUUACGAAACACCCCUGAGACGAUGAAAAGCGUGG
Ga0265757_100063/1301-1090    AAAGCUUUAGGGCCCGUAU.GACCA.AUACGGUUGCAGCCCUCUGCGUGCCG.UC.UUC.GACGGUAAGGCUGUGGUUGGUGU..UGCA..GCACCUUCCACUACCUGUUAGCGCAUAGCUAUCCCUUUCGAAACACCCCUUGAGACGAUGAAAAGCGUG
Ga0310115_100964/2708-2893    AAAGCUUGGGGGCCCGGAU.GACCA.AUCCGGUUGCAGCCCUCUGCGUGCCG.UC.UUC.GACGGUAAGGCUGUGGUUGGUGU..UGCA..GCACCUUCCACUACCUGUUAGCGCAUAGCUACCCUUUCGAAACAACUGCUGAGAC.............
Ga0265745_1034509/411-200     AAAGCUUGGGGGCCCGGAU.GACCA.AUCCGGUUGCAGCCCUCUGCGUGCCG.UC.UUC.GACGGUAAGGCUGUGGUUGGUGU..UGCA..GCACCUUCCACUACCUGUUAGCGCAUAGCUACCCUUUCGAAACAACUGCUGAGACGAUGAAAAGCGUGG

                              .<<<<<.<<<<<<<<<<<<.......>>>>>>.....>>>>>><<<<<<<<<..<<.....>>.>>>.<<<..<<<<.<<<<<<.......>>>>>>.>>>>..>>>....>>>>>.>>>>>.....................................
                              ..1111...0010010110.......011010.....0100..1001...0..10.....01.0...111...10...10111........11101...01...111.....1001.1111......................................
                              ●●R●●●●○○ GG●CCYG ●○-RAYCA-○● CRG●UGCAG●CCUC●GCRYRYC -Y○-UYY-○RYGGUA●●●CY●●GR●Y●G●●●-○UGCA○-●●●C●●●●C●Y A●●●●U○○GYGC● ●●●YA ●● Y●●●RA ●●●   Y  RR●●● ○○  ○○○○○○○
                               5′                                                                                                                    3′
```

alignment positions 210 · · · 221

```
Ga0265745_1003474/129-340     AUCACCCGAAUU
Ga0310106_100179/2926-2750    AUCACGGACGCC
Ga0310115_101079/245-457      AUCACGGACGCC
Ga0265755_100106/171-382      AUCACCCGAAUU
Ga0310106_103451/1-176        AUCACCCGAAUU
Ga0265755_103597/828-616      AUCACGGACGCC
Ga0310106_100179/223-11       AUCACGGACGCC
Ga0265750_1030526/79-291      AUCACGGACGCC
Ga0265757_102423/182-394      AUCACGGACGCU
Ga0265757_100085/127-339      AUCACCCGAAUU
Ga0265740_1197481/141-312     ............
Ga0265755_101715/840-628      ACGCAUCUCAUG
Ga0265745_1111090/67-279      ACGCAUCUCAUG
Ga0265745_1000046/1508-1297   UACGCAUCUCAU
Ga0265757_100063/1301-1090    GUACGCAUCUCA
Ga0310115_100964/2708-2893    ............
Ga0265745_1034509/411-200     UACGCAUCUCAU

                              ............
                              ............
                              ○○○○○○○○○○○○
```

18 HRIMA-15

18.1 Multiple-sequence alignment

For a description of this alignment, see Supplementary Table 2.

Each ribozyme in this alignment is identified by its genomic location in the form SEQID/START-END. SEQID (the sequence accession) is derived from sources such as IMG/M or GenBank. START is the coordinate of the 5′ nucleotide of the hairpin ribozyme, and END corresponds to the 3′ nucleotide. If START>END, then the ribozyme is on the reverse complement strand.

Nucleotides proposed to basepair as part of the consensus structure are shaded in color when they comprise Watson-Crick or G-U pairs. Otherwise they are shaded gray. Conserved stems are also indicated at the bottom of the alignment by angle brackets, where matching $<$ and $>$ denote base-paired columns. Below these angle brackets, the symbol "2" denotes base pairs exhibiting covariation according to the statistically well-founded R-scape method. "1" denotes base pairs exhibiting covariation according to R2R's simplistic method. "0" denotes base pairs that are not observed to mutate and "?" denotes base pairs that have a significant frequency of non-canonical nucleotides for Watson-Crick or G-U pairs ($> 5\%$). Below these base pair annotation is the consensus sequence: "R" = "A" or "G", "Y" = "C" or "U", red nucleotides: nucleotide identity conserved more than 97% of the time, black nucleotides: 90%, gray nucleotides: 75%, red circle (•): nucleotide is present 97% of the time, black circle (•): 90%, gray circle (•): 75%, white circle (◦): 50%. All percentages of sequences just described (e.g. 97% conserved) assume that sequences have been weighted by the GSC algorithm implemented by the Infernal software package.

The alignment begins on the next page.

alignment positions 1 · · · 67

```
Ga0257136_1059838/145-211   UAAACCUGCAUGCUUAAGUGCAUCACGUUCUCGAACGUUCUACGCCGUAUAUGUGCGGGCGGGUGGC
Ga0153880_1327030/152-217   UCAACGCGAGUGCUUAAGUGCACCACGC.UUCGAGCGUUCUACGUCGUAUUUGUACGAUCGCGGACU
Ga0153880_1079145/630-565   UCUAGACGAGUGCUUAAGUGCACCACGC.UUCGAGCGUUCUACGUCGUAUUUGUACGAUCGUCGACA
Ga0257141_1100114/138-73    UGAAACUGAGUGCUUAAGUGCACCACGU.CUCGAACGUUCUACGUCAUAUUUUUAUGAUCGGUUGGA
Ga0257142_1121341/229-164   UGAAACCUAGUGCUUAAAUGCACCACGU.CUCGAACGUUCUACGUCGUAUUUGUACGAUCGGCUGGA
Ga0257141_1118781/292-357   UGAAACCGAGUGCUUAAAUGCACCACGU.CUCGAACGUUCUACGUCGUAUUUGUACGAUCGGCUGGA
                            .....<<<<<<<<......>>>>.<<<<......>>>>......<<<<<....>>>>>>>>>.....
                            .....2..21000......0001.0001......1000......101.0....0.1012..2.....
                            U• A••Y •RUGCUUAA UGCAYCACGY-YUCGARCGUUCUACGYC UAU•U UR GR•CG•••R••
                            5'
```

19 HRIMA-16

19.1 Multiple-sequence alignment

For a description of this alignment, see Supplementary Table 2.

Each ribozyme in this alignment is identified by its genomic location in the form SEQID/START-END. SEQID (the sequence accession) is derived from sources such as IMG/M or GenBank. START is the coordinate of the 5′ nucleotide of the hairpin ribozyme, and END corresponds to the 3′ nucleotide. If START>END, then the ribozyme is on the reverse complement strand.

Nucleotides proposed to basepair as part of the consensus structure are shaded in color when they comprise Watson-Crick or G-U pairs. Otherwise they are shaded gray. Conserved stems are also indicated at the bottom of the alignment by angle brackets, where matching $<$ and $>$ denote base-paired columns. Below these angle brackets, the symbol "2" denotes base pairs exhibiting covariation according to the statistically well-founded R-scape method. "1" denotes base pairs exhibiting covariation according to R2R's simplistic method. "0" denotes base pairs that are not observed to mutate and "?" denotes base pairs that have a significant frequency of non-canonical nucleotides for Watson-Crick or G-U pairs ($> 5\%$). Below these base pair annotation is the consensus sequence: "R" = "A" or "G", "Y" = "C" or "U", red nucleotides: nucleotide identity conserved more than 97% of the time, black nucleotides: 90%, gray nucleotides: 75%, red circle (•): nucleotide is present 97% of the time, black circle (•): 90%, gray circle (•): 75%, white circle (◦): 50%. All percentages of sequences just described (e.g. 97% conserved) assume that sequences have been weighted by the GSC algorithm implemented by the Infernal software package.

The alignment begins on the next page.

alignment positions 1 · · · 60

```
Ga0265757__100063/2700-2820   GUGGUGAGACUGACCGGUGAUGCGGCGGUCUUGCUACGAUUACACACGUGACC..AACUC
Ga0265745__1006538/31-152     GUGAUGGGACUGACCGGUGACGCGGCGGUCCUGUCACGAUUACACAUGUGACC.AAAUUC
Ga0265755__101873/252-130     GUGAUGGGACUGACCGGUGAUGCGGCGGUCCUGUCACGAUUACACAUGUGACCAAAACUC
Ga0265745__1000046/31-151     GUGAUGGGACUGACCGGUGAUGCGGCGGUCUUGUCACGCUUACACAUGUGAUC..AACUC

                              <<<<<.<<<<<<.<<<......>>>>>>>>>.>>>>>..<<<<....>>>>.........
                              00.10.100000.000......000000001.01.00..0000....0000.........
                              GUGRUGRGACUGACCGGUGA GCGGCGGUCYUGYYACG UUACACAYGUGA C--AA UC
                              5′
```

20 HRIMA-17

20.1 Multiple-sequence alignment

For a description of this alignment, see Supplementary Table 2.

Each ribozyme in this alignment is identified by its genomic location in the form SEQID/START-END. SEQID (the sequence accession) is derived from sources such as IMG/M or GenBank. START is the coordinate of the 5′ nucleotide of the hairpin ribozyme, and END corresponds to the 3′ nucleotide. If START>END, then the ribozyme is on the reverse complement strand.

Nucleotides proposed to basepair as part of the consensus structure are shaded in color when they comprise Watson-Crick or G-U pairs. Otherwise they are shaded gray. Conserved stems are also indicated at the bottom of the alignment by angle brackets, where matching < and > denote base-paired columns. Below these angle brackets, the symbol “2” denotes base pairs exhibiting covariation according to the statistically well-founded R-scape method. “1” denotes base pairs exhibiting covariation according to R2R’s simplistic method. “0” denotes base pairs that are not observed to mutate and “?” denotes base pairs that have a significant frequency of non-canonical nucleotides for Watson-Crick or G-U pairs (> 5%). Below these base pair annotation is the consensus sequence: “R” = “A” or “G”, “Y” = “C” or “U”, red nucleotides: nucleotide identity conserved more than 97% of the time, black nucleotides: 90%, gray nucleotides: 75%, red circle (•): nucleotide is present 97% of the time, black circle (•): 90%, gray circle (•): 75%, white circle (◦): 50%. All percentages of sequences just described (e.g. 97% conserved) assume that sequences have been weighted by the GSC algorithm implemented by the Infernal software package.

The alignment begins on the next page.

alignment positions 1 · · · 99

```
Ga0179943_1102267/365-445  AGGCACAGCCACUGUCUUGUCGACCCC..UGA.......GU.A......A..GGGGCGGUGGCUGAGUCUGUUGAGGGUGACGCGUUUACGCGUGUUGC
Ga0179944_1005311/107-26   AGGCACAGCCACUGUCAUGUCGACC..........CCCUGCGAAGGG.......GGCGGUGGCUGAGUCUGGUGAGUGUGGCGCGUUUACGCGCGUCAC
Ga0179943_1150468/393-491  AGGCACUGCCACUGUCUUGUCGACCACGGCUUCCACCCUGUGAAGGGCUGCCGUGGCGGUGGUAGAGUCUGUUGAGGGUGACGCGUUUACGCGUGUCGC
Ga0179944_1191552/120-22   AGGCACAACCACUGUCUUGUCGACCACGGCUUCCACCCUGCGAAGGGCUGCCGUGGCGGUGGCUGAGUCUGUUGAGGGUGACGCGUUUACGCGUGUUGC

                           .......................................<<<<....................................................>>>>
                           .......................................0..0....................................................0..0
                           <<<<.<<<<<<<<<<<<.....>><<<<<<<.....<<<<....>>>>..>>>>>>>>>>>>>>>>>.>>>>..........<<<<<....>>>>>.....
                           0000.02.0000000000.....000010001.....0000....0000..1000100000000.20.0000..........10000....00001.....
                           AGGCAC  CCACUGUC•UGUCGACC∘∘--∘∘∘---∘∘∘∘GY∘A∘∘∘∘--∘--∘∘GGCGGUGG  GAGUCUG•UGAG•GUGRCGCGUUUACGCGYGUYRC
                           5′
```

21 HRIMA-18

21.1 Multiple-sequence alignment

For a description of this alignment, see Supplementary Table 2.

Each ribozyme in this alignment is identified by its genomic location in the form SEQID/START-END. SEQID (the sequence accession) is derived from sources such as IMG/M or GenBank. START is the coordinate of the 5′ nucleotide of the hairpin ribozyme, and END corresponds to the 3′ nucleotide. If START>END, then the ribozyme is on the reverse complement strand.

Nucleotides proposed to basepair as part of the consensus structure are shaded in color when they comprise Watson-Crick or G-U pairs. Otherwise they are shaded gray. Conserved stems are also indicated at the bottom of the alignment by angle brackets, where matching $<$ and $>$ denote base-paired columns. Below these angle brackets, the symbol "2" denotes base pairs exhibiting covariation according to the statistically well-founded R-scape method. "1" denotes base pairs exhibiting covariation according to R2R's simplistic method. "0" denotes base pairs that are not observed to mutate and "?" denotes base pairs that have a significant frequency of non-canonical nucleotides for Watson-Crick or G-U pairs ($> 5\%$). Below these base pair annotation is the consensus sequence: "R" = "A" or "G", "Y" = "C" or "U", red nucleotides: nucleotide identity conserved more than 97% of the time, black nucleotides: 90%, gray nucleotides: 75%, red circle (•): nucleotide is present 97% of the time, black circle (•): 90%, gray circle (∘): 75%, white circle (∘): 50%. All percentages of sequences just described (e.g. 97% conserved) assume that sequences have been weighted by the GSC algorithm implemented by the Infernal software package.

The alignment begins on the next page.

alignment positions 1 · · · 65

```
Ga0242646_1000880/533-483     GUUCCCUCUAUU....GAGGAAGGAUGUGAC........GACAGCUGCU.GAGUAACGAUC.AGC
Ga0247554_107848/330-277      AUCCCCCCUCUCUUU.AGGGAAG.....UAU....GAAUGACAGCCGAGAGCCUAACGAGC.CUC
Ga0184573_100965/270-217      CUCCCCCCCCCACUU.AAGGAAA.....GAG....GAAUGAUAGCUGAGAGCCUAACGAGC.CUC
Ga0316034_100770/487-434      CUCCCCCCCCCACUU.AAGGAAA.....GAG....GAAUGAUAGCUGGGAGCCUAACGAGC.CUC
Ga0184589_133493/559-506      CUCCCCCCCCAACUA.AGGGAAA.....GAG....GAAUGAUAGCUGAGAGCCUAACGAGC.CUC
Ga0184580_114961/492-546      CUCCACCCCCCACUUAAGGGAAA.....GAG....GAAUGAUAGCUGAGAGCCUAACGAGC.CUC
Ga0247524_104389/1120-1066    CUCCCCCCCCCACUCAAGGGAAA.....GAG....GAAUGAUAGCUGAGAGCCUAACGAGC.CUC
Ga0184600_141078/63-117       CUCCACCCCCCACUCAAGGGAAA.....GAG....GAAUGAUAGCUGAGAGCCUAACGAGC.CUC
Ga0247529_103231/1397-1344    CUCCCCCCCCCACUC.UGGGAAA.....GAG....GAAUGAUAGCUGAGAGCCUAACGAGC.CUC
Ga0247527_115808/116-63       GUCCCCCCUCUACUU.GGGGAAA.....GAC....UAAUGACAGCCGAGAGCCCAACGAGC.CUC
Ga0265756_101568/819-766      GUCCCCCCUCUACUU.GGGGAAA.....GAC....CAAUGACAGCCGAGAGCCUAACGAGC.CUC
Ga0184596_109261/814-761      GUCCCCCCUCUACUU.GGGGAAA.....GAC....UAAUGACAGCCGAGAGCCUAACGAGC.CUC
Ga0184602_128425/595-542      GUCCCCCCUCAACUU.GGGGAAA.....GAC....UAAUGACAGCCGAGAGCCUAACGAGC.CUC
Ga0242646_1001007/964-1017    UCUAUCCCUCUAUUG.GGGAAAG.....AGA...UUGAUGACAGCCGGA.GCCUAACGAGC.UUC
Ga0247551_100861/371-417      CCCCCCAUUU......GUGCAAA.....GGG....UAGUGACAGC.CACAGCCCAACGAGC..UG
Ga0247545_103817/459-507      .CCCCCUCUUUU....GUGCAAA.....GGG....UAGUGACAGCUGUU.GCCUAACGAGC.AAC
Ga0247538_113886/218-266      CCCCCCAUUUU.....GUGCACA.....GGG....UAGUGACAGCUGUU.GCCUAACGAGC.AGC
Ga0247551_101323/727-679      CCCCCCAUUUU.....GUGCAAA.....GGG....UAGUGACAGCUGUU.GCUUAACGGGC.AAC
Ga0247526_138727/153-105      CCCCCCAUUUU.....GUGCAAA.....GGG....UAGUGACAGCUGUU.GCCUAACGAGC.AAC
Ga0247553_127775/240-288      CCCCCCAUUUU.....GUGCAAC.....GGG....UAGUGACAGCUGUU.GCCUAACGAGC.AAC
Ga0247543_100559/281-329      CCCCCCAUUUU.....GUGCAAA.....GGG....UAGUGACAGCCACA.GCCCAACGAGC.UGU
Ga0247553_101128/821-774      CCCCCCAUUU......GUGCAAA.....GGG....UAGUGACAGCCACA.GCCGAACGAGC.UGU
Ga0184585_152466/918-867      CCCUCCACUA......GUGCAAA.....GGGAUUCUAAUGACAGCUGCA.GCCUAACGAGC.UGC
Ga0184600_123604/122-173      CCCUCCACUA......GUGCAAG.....GGGAUUCUAAUGACAGCUGCA.GCCUAACGAGC.UGC
Ga0184600_136984/107-55       CCCCCCAUCUU.....GUGCAAG.....GGGAUUUUAAUGACAGCUGCA.GCCUAACGAGC.UGC
Ga0316040_137777/9-57         CCCCCCACUUU.....GUGCAAA.....GGG....UAGUGACAGCUGCA.GCCUAAUGAGC.UGC
Ga0184600_135664/109-157      CCCCCCACUUU.....GUGCAAA.....GGG....UAGUGACAGCUGCA.GCCUAACGAGC.UGC
Ga0184603_124502/130-178      CCCCCCACUUU.....GUGCAGA.....GGG....UAGUGACAGCUGCA.GCCUAACGAGC.UGC
Ga0247524_108630/500-452      CCCCCCACUUU.....GUGCAAA.....GGG....UGAUGACAGCUGCA.GCCUAACGAGC.UGC
Ga0184589_102696/357-309      CCCCCCACUGU.....GUGCAGA.....GGG....UAAUGACAGCCGCA.GCCUAACGAGC.UGC
Ga0316050_120273/381-329      CCCCCCAUUUU.....GUGCAGA.....GGGAUUCUAAUGACAGCCGCA.GCCUAACGAGC.UGC
Ga0184600_118738/115-163      CCCCCCACUGU.....GUGCAGA.....GGG....UGAUGACAGCUGCA.GCCUAACGAGC.UGC
Ga0184593_101068/155-107      CCCCCCACUUU.....GUGCAAA.....GGG....AAGUGACAGCCGCA.GCCUAACUAGU.UGC
Ga0184590_112828/724-676      CCCCCCACUUU.....GUGCAAA.....GGG....AAGUGACAGCCGCA.GCCUAACCAGU.UGC
Ga0184596_102115/88-40        CCCCCCACUUU.....GUGCAAG.....GGG....AAGUGACAGCCGCA.GCCUAACGAGU.UGC
Ga0184602_117226/565-517      CCCCCCACUUU.....GUGCAAG.....GGG....AAGUGACAGCCGCA.GCCUAACUAGC.UGC
Ga0184578_125145/766-718      CCCACCACUU......GUGCAAC.....GGG...UUAAUGACAGCCGCA.GCCUAACGAGC.UGC
Ga0247542_103320/528-476      CCCCCCACUUU.....GUGCAAG.....GGG.AUUUAAUGACAGCCGCA.GCCUAACGAGCAUGC

                              <<<.<<<<........>>>>........>>>...............<<<.<<.......>>.>>>
                              221..01.........10.........122...............222..1.......1..222
                              •Y  C•    ◦---- •G•AAR----- R•----◦  GA AGCY ••-G   AACGA C-••
                              5′
```

22 HRIMA-19

22.1 Multiple-sequence alignment

For a description of this alignment, see Supplementary Table 2.

Each ribozyme in this alignment is identified by its genomic location in the form SEQID/START-END. SEQID (the sequence accession) is derived from sources such as IMG/M or GenBank. START is the coordinate of the 5′ nucleotide of the hairpin ribozyme, and END corresponds to the 3′ nucleotide. If START>END, then the ribozyme is on the reverse complement strand.

Nucleotides proposed to basepair as part of the consensus structure are shaded in color when they comprise Watson-Crick or G-U pairs. Otherwise they are shaded gray. Conserved stems are also indicated at the bottom of the alignment by angle brackets, where matching < and > denote base-paired columns. Below these angle brackets, the symbol "2" denotes base pairs exhibiting covariation according to the statistically well-founded R-scape method. "1" denotes base pairs exhibiting covariation according to R2R's simplistic method. "0" denotes base pairs that are not observed to mutate and "?" denotes base pairs that have a significant frequency of non-canonical nucleotides for Watson-Crick or G-U pairs (> 5%). Below these base pair annotation is the consensus sequence: "R" = "A" or "G", "Y" = "C" or "U", red nucleotides: nucleotide identity conserved more than 97% of the time, black nucleotides: 90%, gray nucleotides: 75%, red circle (•): nucleotide is present 97% of the time, black circle (•): 90%, gray circle (•): 75%, white circle (◦): 50%. All percentages of sequences just described (e.g. 97% conserved) assume that sequences have been weighted by the GSC algorithm implemented by the Infernal software package.

The alignment begins on the next page.

```
                                   alignment positions 1···81

Ga0157560_1137762/17-91       UGUAGAACUAAUGAGAGAAAUAACGUAGAGCAUCUAGUAUAACAAAGAUGA.UA.CGAAUUCCAAAGACAC....UCUACA
Ga0213850_1460186/273-348     UACACA....GUGAGCAAUUCACCGGUAAGUCACCCCCCUGAGG.GGUGAACAUCCGAAAGCUUUCAAAACGAGCUGUGUA
Ga0242654_10185985/310-237    GUUAAA..CUAAUGAGAAGAUAACGUGGAGUGAGUAGUAUAACAAACUCAA.CA.CGACAAUUCCAAAGACA...UUUAAU
Ga0153880_1457361/523-452     UCCCCG....GGGAAUGAGUCAACGGCAAGUCCCAUCUA...GUAUGGGUA.GUACGAUACUUUUUGAAAGAAG.CGGGGA
Ga0255280_1275301/282-209     CUUUGG....CUGGUGCAGUAACAGGCUAGUCCCUAGUAAAUCAAAGGGAA.GU.CUAAAUCUUUUU.AACGAGGUCAAAG
Ga0179944_1264236/356-430     GUGCUA....AGUAAUCAACGGACGUUGAGUCCAUAGUAAAGCAAAUGGAG.AA.CGAAAGUCUCCAAAAAGAGAUAGCAC
Ga0307324_151217/67-141       GUGCUA....AGUAAUCAACGGACGUUGAGUCCAUAGUAAAGCAAAUGGAG.AA.CGAAAGUCUCCAAGAAGAGAUAGCAC
Ga0079075_1228828/378-304     GUGCUA....AGUAAUCAACGGACGUUGAGUCCAUAGUAAAGCAAAUGGAG.AA.CGAAAGUCUCCUAGAAGAGAUAGCAC
Ga0307357_127728/259-185      GUGCUA....AGUAAUCAACGGACGUUGAGUCCAUAGUAAAGCAAAUGGAG.AA.CGAAAGUCUCCCAGAAGAGAUAGCAC
Ga0179955_1195610/558-484     CUUGAA....AGUAAUCAAAGGACGUUGAGUCCAUAGUAGAUCAAAUGGUC.GA.CGAAAUCAACCAAAAAGAGAUUCAAG
Ga0157560_1113202/506-579     ACUUGU....UGGUCUGAUACAGCGUGAAGUCUAUACUAUAUGAAAUAGAG.CG.CGUUAAUUUUUUGAACAAG.AUAAGU
Ga0157570_1052522/769-695     UGCUCG....AGUAUUGAGUCAGCGUGGAGUCCUUAGCAAAACAAAAGGAA.CA.CGAAAUUAUUCAGAAAGAGACGAGCA
Ga0157560_1137762/284-210     UGCUCG....AGUAUUGAGUCAGCGUGGAGUCCUUAGUAAAACAAAAGGAA.CA.CGAAAUUAUUCAGAAAGAGACGAGCA
Ga0115595_1188237/17-90       UCAUGU....UUGAAAACAUAACAGUUGAAUCAGUAGUAAAUCAAACUGAC.AA.CUAAAAUUUUUGAAAAAAG.ACAUGA
Ga0256350_1225191/229-302     UUAGGA....GGGGAAGACUAACAGUGGAGUCACUACUAAAAGAAAGUGAA.CA.CUAAAUCUUCAAAAACUUC.UCCUAA
Ga0210323_1078340/140-69      AGUUAU....GGGGCAAACAAACAGUGGAGUC.CUAGUAAAACAAAG.GAG.CA.CUAUAUUCUUCAAAACGAA.AUAAUU
Ga0179943_1073922/112-41      AGUUAU....GGGGCAGACAAACAGUGGAGUC.CUAGUAAAACAAAG.GAG.CA.CUAAAUUCUUCAAAACGAA.AUAAUU
Ga0179940_1085837/372-443     AGUUAU....GGGGCAGACAAACAGUGGAGUC.CUAGUAAAACAAAG.GAG.CA.CUAUAUUCUUCAAAACGAA.AUAAUU
Ga0210324_1316800/430-503     GAAUUG....UGGGAAAAAUAGCAGUGGAAUGCGUAGUAAAGCAAACGCAA.CA.CUAAAAUCCUCGAAACGAG.CAAUUC
Ga0228709_1019412/682-755     GAAUUA....UGGGAAAAAUGACAGUGGAGUGCGUAGUAAAACAAACGCAA.CA.CUAUAUUCUUCAAAACGAG.UAAUUC

                              <<<<<<.................<<<<...<<<<<..........>>>>>..>>.>>..................>>>>>>
                              222222.................2022....2222..........2222...22.02..................222222
                              5′
```

23 HRIMA-20

23.1 Multiple-sequence alignment

For a description of this alignment, see Supplementary Table 2.

Each ribozyme in this alignment is identified by its genomic location in the form SEQID/START-END. SEQID (the sequence accession) is derived from sources such as IMG/M or GenBank. START is the coordinate of the 5′ nucleotide of the hairpin ribozyme, and END corresponds to the 3′ nucleotide. If START>END, then the ribozyme is on the reverse complement strand.

Nucleotides proposed to basepair as part of the consensus structure are shaded in color when they comprise Watson-Crick or G-U pairs. Otherwise they are shaded gray. Conserved stems are also indicated at the bottom of the alignment by angle brackets, where matching < and > denote base-paired columns. Below these angle brackets, the symbol "2" denotes base pairs exhibiting covariation according to the statistically well-founded R-scape method. "1" denotes base pairs exhibiting covariation according to R2R's simplistic method. "0" denotes base pairs that are not observed to mutate and "?" denotes base pairs that have a significant frequency of non-canonical nucleotides for Watson-Crick or G-U pairs (> 5%). Below these base pair annotation is the consensus sequence: "R" = "A" or "G", "Y" = "C" or "U", red nucleotides: nucleotide identity conserved more than 97% of the time, black nucleotides: 90%, gray nucleotides: 75%, red circle (•): nucleotide is present 97% of the time, black circle (•): 90%, gray circle (•): 75%, white circle (◦): 50%. All percentages of sequences just described (e.g. 97% conserved) assume that sequences have been weighted by the GSC algorithm implemented by the Infernal software package.

The alignment begins on the next page.

alignment positions 1 · · · 115

```
Ga0184577__120131/573-465  GCCACCUAUUGUGGCUUGUGUACUUCCCUAUCGAUUCCAAAGGAGUCUUUGACUGUACGGUAGGACAGUACUGGGUUU.UA.GGU.AUCGCUAAAA..AGGAUACUAU.AAAUCU
Ga0247517__121471/36-144   GCCACCUAUUGUGGCUUGUGUACUUCCCUAUCGAUUCCAAAGGAGUCUUUGACUCUACGGUAGGACAGUACUGGGUUU.UA.GGU.AUCGCUAAAA..AGGAUACUAU.AAAUCU
Ga0247539__101233/58-169   CAGCCCAA.UGGCUGUUGUGUACUUCCCUAGCGAUUCCAAAGGAGUCUUUGACUCUACGCUAGGACAGUACUGGGUUU.AUUGGAUACU.CUAUCACUAGAGUUCCGAUUGAUCC
Ga0247551__102202/290-400  CAGCCCAA.UGGCUGUUGUGUACUUCCCUAGCGAUUCCAAAGGAGUCUUUGACUCUA.GCUAGGACAGUACUGGGUUU.GUUGGAUACU.CUAUCACUAGAGUUCCGAUUGAUCC
Ga0247521__112559/673-562  CAGCCCAA.UGGCUGUUGUGUACUUCCCUAGCGAUUCCAAAGGAGUCUUUGACUCUACGCUAGGACAGUACUGGGUUU.GUUGGAUACU.CUAUCACUAGAGUUCCGAUUGAUCC
Ga0247533__102552/547-658  CAGCCCAA.UGGCUGUUGUGUACUUCCCUAGCGAUUCCAAAGGAGUCUUUGACUCUACGCUAGGACAGUACUGGGUUUGUUGGACUUCUCUCACGA..GAAGGGUCCUCAGAUCC

                           <<<<<.....>>>>>....<<<<<..<<<<<<<........<<<<<<...>>>>>>.>>>>>>>..>>>>>.<<<<<..<..<<<.<<<.<<......>>>>>>>>...>>>>>>
                           11110.....01111....00000..0000100........0.0000...0000.0.0010000..00000..000...2...12.211.11......1111221....2.000.
                           ●●●●CC●A-UG●●●●UUGUGUACUUCCCUA●CGAUUCCAAAGGAGUCUUUGACU UACG●UAGGACAGUACUGGGUUU-●●○GR●○●YY○YYA●●A--RRRR●●YY●●○●RAUCY
                           5′
```

24 HRIMA-21

24.1 Multiple-sequence alignment

For a description of this alignment, see Supplementary Table 2.

Each ribozyme in this alignment is identified by its genomic location in the form SEQID/START-END. SEQID (the sequence accession) is derived from sources such as IMG/M or GenBank. START is the coordinate of the 5′ nucleotide of the hairpin ribozyme, and END corresponds to the 3′ nucleotide. If START>END, then the ribozyme is on the reverse complement strand.

Nucleotides proposed to basepair as part of the consensus structure are shaded in color when they comprise Watson-Crick or G-U pairs. Otherwise they are shaded gray. Conserved stems are also indicated at the bottom of the alignment by angle brackets, where matching $<$ and $>$ denote base-paired columns. Below these angle brackets, the symbol "2" denotes base pairs exhibiting covariation according to the statistically well-founded R-scape method. "1" denotes base pairs exhibiting covariation according to R2R's simplistic method. "0" denotes base pairs that are not observed to mutate and "?" denotes base pairs that have a significant frequency of non-canonical nucleotides for Watson-Crick or G-U pairs ($> 5\%$). Below these base pair annotation is the consensus sequence: "R" = "A" or "G", "Y" = "C" or "U", red nucleotides: nucleotide identity conserved more than 97% of the time, black nucleotides: 90%, gray nucleotides: 75%, red circle (●): nucleotide is present 97% of the time, black circle (●): 90%, gray circle (●): 75%, white circle (○): 50%. All percentages of sequences just described (e.g. 97% conserved) assume that sequences have been weighted by the GSC algorithm implemented by the Infernal software package.

The alignment begins on the next page.

alignment positions 1 · · · 63

```
Ga0247537__109516/182-241     GAUCAUGAGUGA.AUGGCAUGCCCAU.AAAAGUCAUGUAGCUGG.GAAUAACAGGAAUGUAUA
Ga0310106__102264/996-1056    GAUCAUCAGUGAUAUCUGUAA.GGAUAAUAGCUGAUGUAUC.GGUCAACCACGGGAAUGUGUA
Ga0316047__100629/652-592     GAUCAUCAGUGAUAUCUGCAA.GGAUAAUAGCUGAUGUAAC.GGUCAACAACGGGAAUGUGUA
Ga0265755__100704/1160-1220   GAUCAUCAGUGAUAUCUGCAA.GGAUAAUAGCUGAUGUAUC.GGUCAACAACGGGAAUGUGUA
Ga0310106__108714/726-666     GAUCAUCAGUGAUAUCUGCAA.GGAUAAUAGCUGAUGUAUC.GGUCAACAACAGGAAUGUGUA

                              ...<<<<<....<<<<<.....>>>>>.....>>>>>..........................
                              ...00010....00011.....11000.....01000..........................
                              GAUCAU•AGUGA∘AU•••••R-••AU∘A•AR•U•AUGUA•C-GG∘•AAY ACRGGAAUGURUA
                              5′
```

25 HRIMA-22

25.1 Multiple-sequence alignment

For a description of this alignment, see Supplementary Table 2.

Each ribozyme in this alignment is identified by its genomic location in the form SEQID/START-END. SEQID (the sequence accession) is derived from sources such as IMG/M or GenBank. START is the coordinate of the 5′ nucleotide of the hairpin ribozyme, and END corresponds to the 3′ nucleotide. If START>END, then the ribozyme is on the reverse complement strand.

Nucleotides proposed to basepair as part of the consensus structure are shaded in color when they comprise Watson-Crick or G-U pairs. Otherwise they are shaded gray. Conserved stems are also indicated at the bottom of the alignment by angle brackets, where matching $<$ and $>$ denote base-paired columns. Below these angle brackets, the symbol "2" denotes base pairs exhibiting covariation according to the statistically well-founded R-scape method. "1" denotes base pairs exhibiting covariation according to R2R's simplistic method. "0" denotes base pairs that are not observed to mutate and "?" denotes base pairs that have a significant frequency of non-canonical nucleotides for Watson-Crick or G-U pairs ($> 5\%$). Below these base pair annotation is the consensus sequence: "R" = "A" or "G", "Y" = "C" or "U", red nucleotides: nucleotide identity conserved more than 97% of the time, black nucleotides: 90%, gray nucleotides: 75%, red circle (•): nucleotide is present 97% of the time, black circle (•): 90%, gray circle (•): 75%, white circle (∘): 50%. All percentages of sequences just described (e.g. 97% conserved) assume that sequences have been weighted by the GSC algorithm implemented by the Infernal software package.

The alignment begins on the next page.

alignment positions 1 · · · 77

```
Ga0247524_101842/588-514   GUGCUUUAUGAGGCACGAAUCCGAGGUGU.GGCAGCACCAAGGAAAUCCCUA.ACCAGUGUGAAAAAGAUUCACUGG
Ga0247526_101949/231-305   GUGCUUUAUGAGGCACGAAUCCGAGGUGU.GGCAGCACCAAGGAAAUCCUUA.ACCAGUGUGAAAAAGAUUCACUGG
Ga0184589_132372/1055-981  UCAGUUAUAACACUGAAAACCAAAGGUGUUGGCGGCACCAAUGGAAUCCCCA.ACUCGUGUGAAAA.GAUUCACGAG
Ga0184595_105555/300-374   UCAGCUAUAACGCUGAAAACCAAAGGUGUUGGCGGCACCAAUGGAAUCCCCA.ACUCGUGUGAAAA.GAUUCACGAG
Ga0316036_128041/172-98    UCAGCUAUACAGCCGAGAACCAAAGGUGUUGGCGGCACCAAUGGAAUCCCCA.ACUCGUGUGAAAA.GAUUCACGAG
Ga0316048_100452/228-153   UCAGCUAUAAAGCCGAGAACCAAAGGUGUUGGCGGCACCAAUGGAAUCCCCAAACUCGUGUGAAAA.GAUUCACGAG
Ga0184602_104873/444-518   UCAGCUAUAAAGCCGAGAACCAAAGGUGUUGGCGGCACCAAUGGAAUCCCCA.ACUCGUGUGAAAA.GAUUCACGAG
Ga0184576_114641/178-104   UCAGCUAUAAAGCCGAGAAUCAAAGGUGUUGGCGGCACCAAUGAAAUCCCCA.ACUCGUGUGAAAA.GAUUCACGAG
Ga0184570_119653/69-143    UCAGCUAUAAAGCCGAGAAUCAAAGGUGUUGGAGGCACCAAUGAAAUCCCCA.ACUCGUGUGAAAA.GAUUCACGAG
Ga0316031_154348/247-173   UCAGCUAUAAAGCCGAGAAUCAAAGGUGUUGGAGGCACCGAUGAAAUCCCCA.ACUCGUGUGAAAA.GAUUCACGAG

                           <<<<<......>>>>>...<<<..<<<<<.....>>>>>..>>>..........<<<<<<...........>>>>>>
                           11.11......11.11...201..00000.....00000..102..........011000...........000110
                           •YR•YU•••R  • R• AAYC•RAGGUGU∘GG RGCACCAA•GRAAUCC YA-ACY•GUGUGAAAA-GAUUCAC•RG
                           5′
```

26 HRIMA-23

26.1 Multiple-sequence alignment

For a description of this alignment, see Supplementary Table 2.

Each ribozyme in this alignment is identified by its genomic location in the form SEQID/START-END. SEQID (the sequence accession) is derived from sources such as IMG/M or GenBank. START is the coordinate of the 5′ nucleotide of the hairpin ribozyme, and END corresponds to the 3′ nucleotide. If START>END, then the ribozyme is on the reverse complement strand.

Nucleotides proposed to basepair as part of the consensus structure are shaded in color when they comprise Watson-Crick or G-U pairs. Otherwise they are shaded gray. Conserved stems are also indicated at the bottom of the alignment by angle brackets, where matching < and > denote base-paired columns. Below these angle brackets, the symbol "2" denotes base pairs exhibiting covariation according to the statistically well-founded R-scape method. "1" denotes base pairs exhibiting covariation according to R2R's simplistic method. "0" denotes base pairs that are not observed to mutate and "?" denotes base pairs that have a significant frequency of non-canonical nucleotides for Watson-Crick or G-U pairs (> 5%). Below these base pair annotation is the consensus sequence: "R" = "A" or "G", "Y" = "C" or "U", red nucleotides: nucleotide identity conserved more than 97% of the time, black nucleotides: 90%, gray nucleotides: 75%, red circle (•): nucleotide is present 97% of the time, black circle (•): 90%, gray circle (•): 75%, white circle (∘): 50%. All percentages of sequences just described (e.g. 97% conserved) assume that sequences have been weighted by the GSC algorithm implemented by the Infernal software package.

The alignment begins on the next page.

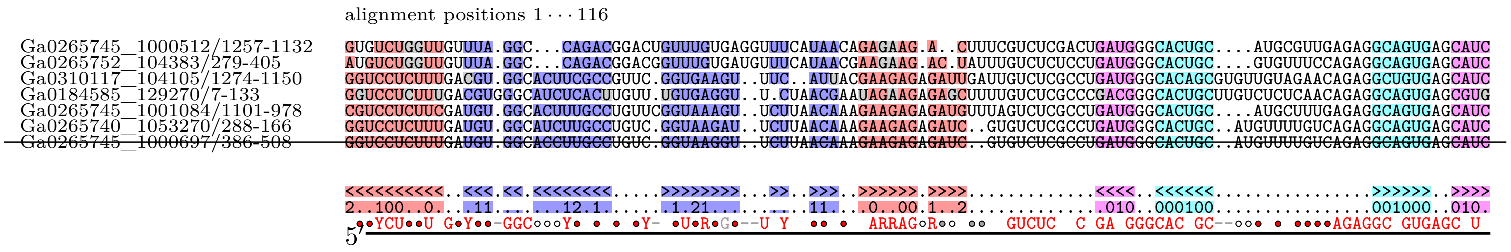

27 HRIMA-24

27.1 Multiple-sequence alignment

For a description of this alignment, see Supplementary Table 2.

Each ribozyme in this alignment is identified by its genomic location in the form SEQID/START-END. SEQID (the sequence accession) is derived from sources such as IMG/M or GenBank. START is the coordinate of the 5′ nucleotide of the hairpin ribozyme, and END corresponds to the 3′ nucleotide. If START>END, then the ribozyme is on the reverse complement strand.

Nucleotides proposed to basepair as part of the consensus structure are shaded in color when they comprise Watson-Crick or G-U pairs. Otherwise they are shaded gray. Conserved stems are also indicated at the bottom of the alignment by angle brackets, where matching < and > denote base-paired columns. Below these angle brackets, the symbol "2" denotes base pairs exhibiting covariation according to the statistically well-founded R-scape method. "1" denotes base pairs exhibiting covariation according to R2R's simplistic method. "0" denotes base pairs that are not observed to mutate and "?" denotes base pairs that have a significant frequency of non-canonical nucleotides for Watson-Crick or G-U pairs ($> 5\%$). Below these base pair annotation is the consensus sequence: "R" = "A" or "G", "Y" = "C" or "U", red nucleotides: nucleotide identity conserved more than 97% of the time, black nucleotides: 90%, gray nucleotides: 75%, red circle (•): nucleotide is present 97% of the time, black circle (•): 90%, gray circle (•): 75%, white circle (◦): 50%. All percentages of sequences just described (e.g. 97% conserved) assume that sequences have been weighted by the GSC algorithm implemented by the Infernal software package.

The alignment begins on the next page.

alignment positions 1 · · · 88

```
Ga0157565_1136042/791-704  AGAACCAAGUGGAUAACACUUAAUUUUCCUGUAAAGCAUGCAUGUUGCCAUGCGAUUCUACUGUUAGCUUCAACAAAGAGAAAGGGUA
Ga0157559_1053445/836-749  AGAAUCAAGUGGAUAACACUUAAUUUUCCUGUAAAGCAUGCAUGUUGCCAUGCGAUUCUACUGUUAGCUUCAACAAAGAGAAAGGGUA
Ga0157560_1098488/66-153   AGAAUCAAGUGGAUAACACUUAAUUUUCCUAUAAACCACAUAUGUGUCUGUGGGAUUCUACUCUUAGCGUCAAUGAUGAGAAAAGCUG
Ga0157565_1096503/733-820  AGAAACGAGUGGAUAACACUCAAUUUUCCUUUAAACCACAUAUGUGUCUGUGGGAUUCUACUGUUAGCGUCAAUACUGAGAAAUGCUA

                           <<<<<<<<<<<.....>>>>>..............<<<<<........>>>>>>>>>>>.............................
                           0000.010000.....00001..............10022........220010.0000.............................
                           AGAA•CRAGUGGAUAACACUYAAUUUUCCU•UAAA•CAYRYAUGU••CYRUG•GAUUCUACU•UUAGC•UCAAYR••GAGAAA•G•UR
                           5′
```

28 HRIMA-25

28.1 Multiple-sequence alignment

For a description of this alignment, see Supplementary Table 2.

Each ribozyme in this alignment is identified by its genomic location in the form SEQID/START-END. SEQID (the sequence accession) is derived from sources such as IMG/M or GenBank. START is the coordinate of the 5′ nucleotide of the hairpin ribozyme, and END corresponds to the 3′ nucleotide. If START>END, then the ribozyme is on the reverse complement strand.

Nucleotides proposed to basepair as part of the consensus structure are shaded in color when they comprise Watson-Crick or G-U pairs. Otherwise they are shaded gray. Conserved stems are also indicated at the bottom of the alignment by angle brackets, where matching $<$ and $>$ denote base-paired columns. Below these angle brackets, the symbol "2" denotes base pairs exhibiting covariation according to the statistically well-founded R-scape method. "1" denotes base pairs exhibiting covariation according to R2R's simplistic method. "0" denotes base pairs that are not observed to mutate and "?" denotes base pairs that have a significant frequency of non-canonical nucleotides for Watson-Crick or G-U pairs ($> 5\%$). Below these base pair annotation is the consensus sequence: "R" = "A" or "G", "Y" = "C" or "U", red nucleotides: nucleotide identity conserved more than 97% of the time, black nucleotides: 90%, gray nucleotides: 75%, red circle (•): nucleotide is present 97% of the time, black circle (•): 90%, gray circle (•): 75%, white circle (◦): 50%. All percentages of sequences just described (e.g. 97% conserved) assume that sequences have been weighted by the GSC algorithm implemented by the Infernal software package.

The alignment begins on the next page.

alignment positions 1 · · · 81

```
Ga0265742_1038020/247-327  ACACCUGUGGGUGUCGCGUGAGCGGACCAAGUUUGGACAAAUCUUGGAUGACCACACUUAACAGAGUGUUAAACAUGCACG
Ga0265747_117353/238-158   ACACCUGUGGGUGUCGCGUGAGCGGACCAAGUUUGGACAAAUCUUGGAUGACCACACUUAAUAGAGUGUUAAACAUGCACG
Ga0265747_148734/229-158   UGACUUCAAAGUCACAUGCGUGU......UGUUUGUGCA...GUUGGAUGACCACUCUUACUGGAGUGUUAAACAUGCGCG
Ga0265756_129985/305-376   UGACUUCAAAGUCACAUGCGUGU......UGUUUGAGCA...GUUGGAUGACCACUCUUACUGGAGUGUUAAACAUGCGCG
Ga0265750_1085374/227-156  UGACUUCAAAGUCAUAUGCGUGU......UGUUUGAGCA...GUUGGAUGACCACUCUUACUGGAGUGUUAAACAUGCGCG
Ga0184583_122581/231-160   UGACUUCAAAGUCAUAUGCGUGU......UGUUUGAUCA...GUUGGAUGACCACUCUUAACGGAGUGUUAAACAUGCGCG
Ga0247552_104528/303-374   UGACUUCAAAGUCAUAUGCGUGU......UGUUUGAACA...GUUGGAUGACCACUCUUAACGGAGUGUUAAACAUGCGCG
Ga0184570_113789/228-157   UGACUUCAAAGUCACAUGCGUGU......UGUUUGUUCA...GUUGGAUGACCACUCUUAACGGAGUGUUAAACAUGCGCG
Ga0184576_109112/146-75    UGACUUCAAAGUCACAUGCGUGU......UGUUUGAUCA...GUUGGAUGACCACUCUUAACGGAGUGUUAAACAUGCGCA
Ga0184568_114501/300-371   UGACUUCAAAGUCACAUGCGUGU......UGUUUGAUCA...GUUGGAUGACCACUCUUAACGGAGUGUUAAACAUGCGCG
Ga0184601_130012/219-148   UGACUUCAAAGUCACAUGUGUGU......UGUUUGAUCA...GUUGGAUGACCACUCUUAACGGAGUGUUAAACAUGCGCG
Ga0316031_121954/225-154   UGACUUCAAAGUCACAUGUGUGU......UGUUUGAUCA...AUUGGAUGACCACUCUUAACGGAGUGUUAAACAUGCGCG

                           <<<<<....>>>>>..<<<<<<.......<<<<<<<................<<<<<<....>>>>>>>>>>>>>>>>>>>
                           11001....10011..1010.0........00000.................000.0......0.000.00000.0.0101
                           ••ACYU••RRGU•• RYGYG•GY------•GUUUG •CA---•UUGGAUGACCAC•CUUA YRGAGUGUUAAACAUGCRCG
                           5′
```

29 HRIMA-26

29.1 Multiple-sequence alignment

For a description of this alignment, see Supplementary Table 2.

Each ribozyme in this alignment is identified by its genomic location in the form SEQID/START-END. SEQID (the sequence accession) is derived from sources such as IMG/M or GenBank. START is the coordinate of the 5′ nucleotide of the hairpin ribozyme, and END corresponds to the 3′ nucleotide. If START>END, then the ribozyme is on the reverse complement strand.

Nucleotides proposed to basepair as part of the consensus structure are shaded in color when they comprise Watson-Crick or G-U pairs. Otherwise they are shaded gray. Conserved stems are also indicated at the bottom of the alignment by angle brackets, where matching $<$ and $>$ denote base-paired columns. Below these angle brackets, the symbol "2" denotes base pairs exhibiting covariation according to the statistically well-founded R-scape method. "1" denotes base pairs exhibiting covariation according to R2R's simplistic method. "0" denotes base pairs that are not observed to mutate and "?" denotes base pairs that have a significant frequency of non-canonical nucleotides for Watson-Crick or G-U pairs ($> 5\%$). Below these base pair annotation is the consensus sequence: "R" = "A" or "G", "Y" = "C" or "U", red nucleotides: nucleotide identity conserved more than 97% of the time, black nucleotides: 90%, gray nucleotides: 75%, red circle (•): nucleotide is present 97% of the time, black circle (•): 90%, gray circle (•): 75%, white circle (◦): 50%. All percentages of sequences just described (e.g. 97% conserved) assume that sequences have been weighted by the GSC algorithm implemented by the Infernal software package.

The alignment begins on the next page.

alignment positions 1 · · · 118

```
Ga0184602_108590/219-333     AGGCGGUUCGGUAA.CCCGAGGGCCAACCGAAUAACC.AAAAAGAGGGUGCAAGGGGCUCCUUUCUAGGGAGCG.GUGCACUAACCUCGCCGUCCGUGAUACAGAGCGAAGAUUCUC
Ga0184590_123576/87-201      AGGCGGUUCGGUAA.CCCGAGGGCCAACCGAAUAACA.AAAAAGAGGGUGCAAGGGGCUCCUUUCUAGGGAGCG.GUGCACUAACCUCGCCGUCCGUGAUACAGAGCGAAGAUUCUC
Ga0184593_101068/838-725     AGGCGGUUCGGUAA.CCCGAGGGCCAACCGAAUAACC.AAAAAGAGGGUGCA.GGGACUCCUUUCUAGGGAGUG.GUGCACUAACCUCGCCGUCAGUGAUACAGAGCGAAGAUUCUC
Ga0184598_145930/451-338     AGGCGGUUCGGUAA.CCCAAGGGCCAACCGAAUAAAC.AAAAAGGGGGUGCACGGGGCUCUU.ACUAGAGGAGCG.AUGCACUAACCCCGCCGUCCGUGAUACAGAGCGAAGAUUCUC
Ga0247526_110221/93-206      AGGCGGUUCGGUAA.CCCAAGGGCCAACCGAAUAACC.AAAAAGGGGGUGCAAGGGGCUCCU.ACUGGAGGAGCG.GUGCACUGACCCCGCCGUCCGUGAUACAGAGCGAAGAUUCUC
Ga0316049_158533/101-214     AGGCGGUUCGGUAA.CCUAAGGGCCAACCGAAUAACC.AAAAAGGGGGUGCAAGGGACUCCU.ACUGGAGGAGUG.GUGCACUGACCCCGCCGUCCGUGAUACAGAGCGAAGAUUCUC
Ga0316051_1010660/329-442    AGGCGGUUCGGUAA.CCUAAGGGCCAACCGAAUAACC.AAAAAGGGGGUGCAAGGGACUCCU.ACUCGAGGAGUG.GUGCACUGACCCCGCCGUCCGUGAUACAGAGCGAAGAUUCUC
Ga0184582_117955/412-306     AGGCGGUUCGGU.....GCCA.....ACCGAAUAACC.AAAAAGGGGGUGCACGGGGCUCCUUUCUAGAGGAGCGGGUGCACUGACCCCGCCGUCCGUGAUACAGAGCGAAGAUUCUC
Ga0184600_142231/408-302     AGGCGGUUCGGU.....GUCA.....ACCGAAUAACC.AAAAAGGGGGUGCACGGGGCUCCUUCCUAGGGGAGCGGAUGCACUGACCCCGCCGUCCGUGAUACAGAGCGAAGAUUCUC
Ga0247529_116375/97-203      AGGCGGUUCGGU.....GCCA.....ACCGAAUAACC.AAAAAGGGGGUGCACGGGGCUCCUUUCUAGGGGAGCGGAUGCACUGACCCCGCCGUCCGUGAUACAGAGCGAAGAUUCUC
Ga0184594_110819/94-198      AGGCGGUUCGGU.....GCCA.....ACCGAAUAACC.AAAAAGGGGGUGCACGGGACUCCU.ACCAGAGGAGUG.AUGCACUGACCCCGCCGUCCGUGAUACAGAGCGAAGAUUCUC
Ga0316031_104979/656-552     AGGCGGUUCGGU.....GCCA.....ACCGAAUAACC.AAAAAGGGGGUGCACGGGACUCCU.UCCAGAGGAGUG.AUGCACUGACCCCGCCGUCCGUGAUACAGAGCGAAGAUUCUC
Ga0247534_102125/646-533     AGGCGGUUCGGUAA.CCCAAGGGUCAACCGAAUAACC.GAAAAGGGGGUGCAUGGGGCUCCU.UCUGGAGGAGCG.AGGCACGAACCCCGCCGUCCGUGAUACAGAGCGAAGAUUCUC
Ga0247519_110701/96-209      UGGCGGUUCGGUAA.CCUAAGGGCCAACCGAAUAACC.AAAAAGGGGGUGUAUGGGGCUCCU.ACCAGAGGAGCG.AGACACUAACCCCGCCGUCCGUGAUACAGAGCGAAGAUUCUC
Ga0184602_114416/523-410     AGGCGGUUCGGUAA.CCCAAGGGCCAACCGAAUAACC.AAAAAGGGGGUGUAUGGGGCUCCU.ACCAGAGGAGCG.AGACACUAACCCCGCCGUCCGUGAUACAGAGCGAAGAUUCUC
Ga0184590_128302/99-212      AGGCGGUUCGGUAA.CCCAAGGGCCAACCGAAUAACC.AGAAAGGGGGUGUAUGGGGCUCCU.ACCAGAGGAGCG.AGACACUAACCCCGCCGUCCGUGAUACAGAGCGAAGAUUCUC
Ga0247520_118066/452-339     AGGCGGUUCGGUAA.CCCAAGGGCCAACCGAAUAACC.AAAAAGGGGGUGUAUGGGGCUCCU.UCUAGAGGAGCG.AGACACUAACCCCGCCGUCCGUGAUACAGAGCGAAGAUUCUC
Ga0184597_119632/82-195      AGGCGGUUCGGUAA.CCUAAGGGCCAACCGAAUAACC.AAAAAGGGGGUGUAUGGGCUCCU.UCUGGAGGAGCG.AGACACUAACCCCGCCGUCCGUGAUACAGAGCGAAGAUUCUC
Ga0184571_105046/77-181      AGGCGGUUCGGU.....GCCA.....ACCGAAUAACC.AAAAAGGGGGUGUAUGGGGCUCCU.UCUGGAGGAGCG.AGACACUAACCCCGCCGUCCGUGAUACAGAGCGAAGAUUCUC
Ga0184570_105326/850-737     AGGCGGUUCGGUAA.CCUAAGGGCCAACCGAAUAACC.AAAAAGGGGGUGUAUGGGGCUCCU.GCUGGAGGAGCG.AGACACUAACCCCGCCGUCCGUGAUACAGAGCGAAGAUUCUC
Ga0184603_106387/271-384     AGGCGGUUCGGUAA.CCUAAGGGCCAACCGAAUAACC.AAAAAGGGGGUGUAUGGGGCUCCU.ACUGGAGGAGCG.AGACACUAACCCCGCCGUCCGUGAUACAGAGCGAAGAUUCUC
Ga0184600_145899/51-164      UGGCGGUUCGGUAA.CCUAAGGGCCAACCGAAUAACC.AAAAAGGGGGUGUAUGGGGCUCCU.ACUGGAGGAGCG.AGACACUAACCCCGCCGUCCGUGAUACAGAGCGAAGAUUCUC
Ga0247524_111427/561-448     UGGCGGUUCGGUAA.CCUAAGGGCCAACCGAAUAACC.AAAAAGGGGGUGUAUGGGGCUCCU.GCUGGAGGAGCG.AGACACUAACCCCGCCGUCCGUGAUACAGAGCGAAGAUUCUC
Ga0247529_134331/174-60      AGGCGGUUCGGUAA.CCCUAGGGCCAACCGAAUAACCAAAAAAGGGGGUGUAUGGGCUCCU.GCUGGAGGAGCG.AGACACUAACCCCGCCGUCCGUGAUACAGAGCGAAGAUUCUC
Ga0184589_129705/103-207     AGGCGGUUCGGU.....GUCA.....ACCGAAUAACC.AAAAAGGGGGUGCACGGGGCUCCU.UCUAGAGGAGCG.AUGCACUAACCCCGCCGUCCGUGAUACAGAGCGAAGAUUCUC
Ga0184578_107475/709-596     AGGCGGUUCGGUAA.CCCAAGGGCCAACCGAAUAACC.AAAAAGGGGGUGCACGGGACUCCU.UCUAGAGGAGUG.AGGCACUAACCCCGCCGUCCGAGAUACAGAGCGAAGAUUCUC
Ga0184580_104559/307-194     AGGCGGUUCGGUAA.CCCAAGGGCCAACCGAAUAACC.AAAAAGGGGGUGCACGGGGCUCCU.UCUAGAGGAGCG.AGGCACUAACCCCGCCGUCCGUGAUACAGAGCGAAGAUUCUC
Ga0184600_142794/372-268     AGGCGGUUCGGU.....GCCA.....ACCGAAUAACC.AAAAAGGGGGUGCACGGGGCUCCU.UCUAGAGGAGCG.AGGCACUAACCCCGCCGUCCGUGAUACAGAGCGAAGAUUCUC
Ga0316052_100392/1524-1637   AGGCGGUUCGGUAA.CCCAAGGGCCAACCGAAUAACC.AAAAAGGGGGUGCACGGGGCUCCA.UCUGGAGGAGCG.AUGCACUAACCCCGCCGUCCGUGAUACAGAGCGAAGAUUCUC
Ga0316053_106722/719-606     AGGCGGUUCGGUAA.CCCAAGGGCCAACCGAAUAAUC.AAAAAGGGGGUGCACGGGGCUCCU.UCUGGAGGAGCG.AUGCACUAACCCCGCCGUCCGUGAUACAGAGCGAAGAUUCUC
Ga0316049_100659/1541-1428   AGGCGGUUCGGUAA.CCCAAGGGCCAACCGAAUAACC.AAAAAGGGGGUGCACGGGGCUCCU.UCUGGAGGAGCG.AUGCACUAACCCCGCCGUCCGUGAUACAGAGCGAAGAUUCUC
Ga0316051_1008702/78-191     AGGCGGUUCGGUAA.CCCAAGGGCCAACCGAAUAAUC.AAAAAGGGGGUGCACGGGGCUCCA.UCUGGAGGAGCG.AUGCACUAACCCCGCCGUCCGUGAUACAGAGCGAAGAUUCUC
Ga0247552_101742/103-216     AGGCGGUUCGGUAA.CCCAAGGGUCAACCGAAUAACC.AAAAAGGGGGUGCACGGGGCUCCU.UCUGGAGGAGCG.AGGCACUAACCCCGCCGUCCGUGAUACAGAGCGAAGAUUCUC
Ga0247536_108342/379-266     AGGCGGUUCGGUAA.CCCAAGGGCCAACCGAAUAACC.GAAAAGGGGGUGCGUGGGGCUCCU.UCUGGAGGAGCG.AUGCACUAACCCCGCCGUCCGUGAUACAGAGCGAAGAUUCUC
Ga0184597_106380/388-479     GGGCGGAUCUCAAAGCUUACGAGCAGAGAGAAUAAUC.AAAAAGGAGGA..........CC..GUAAA.GG............AUCCUUGCCGUCUGUGAUACAGAGCGAAGAUUCUC
Ga0184598_107106/77-168      GGGCGGAUCUCAAAGCUUACGAGCAGAGAGAAUAAUU.AAAAAGAAGGA..........CC..GUAAA.GG............AUCCUUGCCGUCUGUGAUACAGAGCGAAGAUUCUC
Ga0316034_100770/1144-1053   GGGCGGAUCUCAAAGCUUACGAGCAGAGAGAAUAACC.AAAAAGAAGGA..........CC..GUAAA.GG............AUCCUUGCCGUCUGUGAUACAGAGCGAAGAUUCUC
Ga0247524_104389/465-374     GGGCGGAUCUCAAAGCUUACGAGCAGAGAGAAUAAUC.AAAAAGAAGGA..........CC..GUAAA.GG............AUCCUUGCCGUCUGUGAUACAGAGCGAAGAUUCUC
Ga0184596_111007/997-906     GGGCGGACCUCAAAGCUUACGAGCAGAGAGGAUAAUC.AAAAAGUCGGG..........CC..GUAAA.GG............ACCCGUGCCGUCUGUGAUACAGAGCGAAGACUCUC
                             ....<<<<<<<<...<<....>>...>>>>>...>>>.......<<<<<.......<<<<<<......>>>>>>..........>>>>>.............................
                             .......1011.....01....10....1101.............1001.......2000..........0002..........1001..............................
                             RGGCGG• C•••  -    •    ∘ A••G AUAAYC-AAAAAG  GG•∘∘∘∘∘∘∘∘∘∘CC∘-•Y•RR∘GG∘∘∘∘-∘∘∘∘∘∘∘ •CC YGCCGUCYGUGAUACAGAGCGAAGA UCUC
                             5′
```
